# Peripheral insulin resistance attenuates cerebral glucose metabolism and impairs working memory in healthy adults

**DOI:** 10.1038/s44324-024-00019-0

**Published:** 2024-08-02

**Authors:** Hamish A. Deery, Emma Liang, Robert Di Paolo, Katharina Voigt, Gerard Murray, M. Navyaan Siddiqui, Gary F. Egan, Chris Moran, Sharna D. Jamadar

**Affiliations:** 1 School of Psychological Sciences, Monash University, Wellington Rd, Melbourne, VIC, Australia; 2 Monash Biomedical Imaging, Monash University, 770 Blackburn Rd, Melbourne, VIC, Australia; 3 School of Public Health and Preventive Medicine, Monash University, 553 St Kilda Rd, Melbourne, VIC, Australia

**Keywords:** Pre-diabetes, Metabolic diseases

## Abstract

People with insulin resistance are at increased risk for cognitive decline. Insulin resistance has previously been considered primarily a condition of ageing but it is increasingly seen in younger adults. It is possible that impaired insulin function in early adulthood has both proximal effects and moderates or even accelerates changes in cerebral metabolism in ageing. Thirty-six younger (mean 27.8 years) and 43 older (mean 75.5) participants completed a battery of tests, including blood sampling, cognitive assessment and a simultaneous PET/MR scan. Cortical thickness and cerebral metabolic rates of glucose were derived for 100 regions and 17 functional networks. Older adults had lower rates of regional cerebral glucose metabolism than younger adults across the brain even after adjusting for lower cortical thickness in older adults. Higher fasting blood glucose was also associated with lower regional cerebral glucose metabolism in older adults. In younger adults, higher insulin resistance was associated with lower rates of regional cerebral glucose metabolism but this was not seen in older adults. The largest effects of insulin resistance in younger adults were in prefrontal, parietal and temporal regions; and in the control, salience ventral attention, default and somatomotor networks. Higher rates of network glucose metabolism were associated with lower reaction time and psychomotor speed. Higher levels of insulin resistance were associated with lower working memory. Our results underscore the importance of insulin sensitivity and glycaemic control to brain health and cognitive function across the adult lifespan, even in early adulthood.

## Introduction

Insulin is central to the uptake of glucose into cells and the regulation of systemic blood glucose concentrations^1^. Insulin enters the brain via insulin-independent mechanisms, where it used for a range of functions, such as cerebral glucose metabolism, the production of neurotransmitters, growth and regeneration of axons and neurons, circuit development, as well as the regulation of mood, behaviour and cognition^1,2^.

*Insulin resistance* describes how sensitive cells are to the effects of insulin. Greater insulin resistance (i.e., lower insulin sensitivity) results in greater amounts of insulin being required to transport glucose into cells. The underlying aetiology of insulin resistance has been well described (see Ref. ^3^ for a review). As the number of people with insulin resistance has increased^4,5^, so too has research on the pathophysiology of insulin in the brain. Much of this research has focussed on conditions in which insulin resistance is a hallmark characteristic, such as type 2 diabetes, Alzheimer’s disease and ageing^6–8^. This research suggests that peripheral and central insulin resistance are linked^9,10^ and that insulin resistance is an important contributor to age-related diseases and cognitive decline^11–13^.

Insulin resistance, even in those without type 2 diabetes, has been associated with grey matter atrophy and cognitive decline^14,15^. Longitudinal studies have linked peripheral insulin resistance in otherwise metabolically healthy people with subsequent declines in cognitive function^16,17^. Variations in glycaemia in the absence of a clinical diagnoses of prediabetes or insulin resistance can cause grey matter atrophy^18,19^, reduced white matter integrity and impaired cognition in people in their late 20 s, 30 s and 40 s^18–20^.

Although the causes of cognitive decline in insulin resistance are multifaceted, altered cerebral glucose metabolism is considered a major factor^2,8^, with changes in insulin resistance in midlife or earlier becoming increasingly of interest^21^. Although the impact of insulin resistance on whole-brain glucose metabolism is equivocal in healthy older adults^6,22–24^, insulin resistance has been associated with lower glucose uptake regionally, including in the thalamus and caudate^23^, regions of the prefrontal and temporal cortices, cingulate and insula^22^ and the medial orbital frontal cortex^23^. Age-related reductions in cerebral metabolism occur independently of cortical atrophy^25^, indicating that metabolic reductions are not simply a result of loss of tissue or cell bodies in ageing but also occur from a loss of metabolic efficiency or function.

To date, most research exploring the links between insulin resistance and brain glucose metabolism has been with people in mid-to-late life. With greater recognition of the contribution of early adult factors to later life brain health, it is important to better understand whether there are associations between insulin resistance, cerebral glucose metabolism and cognition in younger people with few cardiometabolic risk factors. Understanding these associations can help guide the need for further research or health interventions and is especially important as rates of insulin resistance are growing in younger people^26^.

Here we investigated the associations between age, insulin resistance, cerebral glucose metabolism, and cognition. We hypothesised that: (1) older people would have greater insulin resistance and lower cortical thickness than younger people; (2) older people would have lower regional cerebral metabolic rates of glucose than younger people, even after adjusting for lower cortical thickness in older people; (3) greater insulin resistance would be associated with lower cerebral metabolic rates of glucose and that this association would be moderated by age, with the effect being stronger in older adults; and (4) greater cerebral metabolic rates of glucose and lower insulin resistance would be associated with better cognitive test performance.

## Results

### Sample characteristics

The characteristics of the whole sample (*N* = 79), as well as the younger (*N* = 36) and older (*N* = 43) participants, are shown in Table 1. The mean age of the whole sample was 53.8 years (SD = 24.6). The proportion of women was 52%. The average years of education was 17.5; BMI was 25.0 kg/m^2^, resting heart rate was 78 BPM, systolic and diastolic blood pressure were 136 and 82 mmHg; and cortical thickness was 2.43 mm. Mean fasting blood glucose was 4.99 mmol/L, insulin 4.36 mIU/L, HOMA-IR 1.00 and HOMA-IR2 0.57.

**Table 1:** Demographics for the whole sample and comparison of older and younger groups

	Whole Sample (*N* = 79)		Younger (*N* = 36)		Older (*N* = 43)		Younger vs Older *p* value^a^
	Mean	SD	Mean	SD	Mean	SD	
Age (years)	53.8	24.6	27.9	6.3	75.5	5.8	<0.001
Sex (number and % female)	41 (52%)		21 (58%)		20 (47%)		0.295
Country of birth (% not born in Australia^b^)	36 (51%)		23 (64%)		13 (36%)		0.008
Education (years)	17.5	3.3	18.1	2.9	16.9	3.6	0.167
Body Mass Index (kg/m^2^)	25.0	4.1	24.2	4.7	25.6	3.5	0.133
Resting Heart Rate (bpm)	77.9	15.7	82.7	17.4	73.9	13.0	0.013
Systolic Blood Pressure (mmHg)	135.5	26.0	119.6	17.7	148.8	24.4	<0.001
Diastolic Blood Pressure (mmHg)	81.7	12.7	79.4	13.4	83.6	11.9	0.144
Cortical Thickness (mm)	2.43	0.12	2.52	0.07	2.36	0.11	<0.001
Fasting blood glucose (mmol/L)	4.99	0.54	4.77	0.40	5.17	0.58	<0.001
Fasting insulin (mIU/L)	4.36	2.93	4.76	3.12	4.03	2.74	0.268
HOMA-IR	1.00	0.73	1.02	0.71	0.97	0.75	0.783
HOMA-IR2	0.57	0.39	0.61	0.40	0.53	0.38	0.410
Number of medications in last 24 hours^c^	0.84	1.44	0.28	0.57	1.30	1.8	<0.001
Cigarette smoking in last 6 weeks							0.127
Never	65 (93%)		29 (85%)		36 (100%)		
On one day	1 (1%)		1 (3%)		0 (0%)		
1–2 days in the last 6 weeks	2 (3%)		2 (6%)		0 (0%)		
1–2 days every week	2 (3%)		2 (6%)		0 (0%)		
	2 (3%)		2 (6%)		0 (%)		
Alcohol use in last 6 weeks							0.032
Never	17 (24%)		9 (27%)		8 (22%)		
On one day	9 (13%)		4 (12%)		5 (13%)		
1–2 days in the last 6 weeks	16 (23%)		10 (29%)		6 (17%)		
1–2 days every week	19 (27%)		11 (32%)		8 (22%)		
Everyday	9 (12%)		0 (0%)		9 (25%)		
Illicit drug use in last 6 weeks							0.300
Never	69 (99%)		33 (97%)		36 (100%)		
On one day	1 (1%)		1 (3%)		0 (0%)		
1–2 days in the last 6 weeks	0 (0%)		0 (0%)		0 (0%)		
1–2 days every week	0 (0%)		0 (0%)		0 (0%)		
Everyday	0 (0%)		0 (0%)		0 (0%)		
HVLT: Delayed recall (total)	8.29	2.66	9.39	2.49	7.37	2.47	<0.001
HVLT: Recognition discrimination index	9.83	2.00	10.63	1.70	9.19	2.02	<0.001
Digit Span: Forward (longest)	6.75	1.30	6.64	1.53	6.84	1.07	0.502
Digit Span: Backwards (longest)	5.28	1.28	5.47	1.34	5.12	1.22	0.221
Category Switch: RT in switch trials (sec)	1.80	0.64	1.42	0.40	2.12	0.63	<0.001
Category Switch: SSRT	394.6	354.2	284.1	244.8	487.1	404.7	0.010
Digit Substitution: Correct count	44.5	22.0	64.0	12.2	28.6	13.8	<0.001
Digit Substitution: Sec per correct count	4.37	6.95	3.55	9.68	5.08	2.95	0.017
Stop Signal: Mean stop signal delays (sec)	0.33	0.16	0.28	0.15	0.38	0.16	0.009
Stop Signal: Stop signal reaction time (sec)	0.56	0.12	0.52	0.11	0.59	0.12	0.004
WASI FSIQ2 T-score	116.8	15.3	109.0	12.1	123.2	14.8	<0.001

Continuous variables are mean (standard deviation); categorical variables are number and %.

^a^*P* values are based on T-test for continuous and Ch-square for categorical variables.

^b^Six participants did not provide data. Self-reported cultural background is provided in the Supplementary Table S1.

^c^See Supplementary Table 2 for medication classes.

The mean age of the younger group was 27.8 years (SD = 6.2) and the older group 75.5 years (SD = 5.8). The proportion of women was higher in the younger group (58%) than the older group (47%) but this difference was not statistically significant (*p* = 0.295). The average years of education was similar in the younger group (18.1) and the older group (17.1). These average years of education are slightly higher than the adult population in Australia (50% have at least a Bachelor’s degree, equivalent to at least 15 years of education)^27^. A higher proportion of younger (64%) than older (36%) adults was born outside of Australia (*p* = 0.008). The percentage of adults who were born overseas in the older group is on par with the percentage in the Australia adult population^28^. However, the younger adult group percentage in our sample born overseas was lower than that in the Australian adult population. Our younger adult sample included a high percentage of participants from south-east and central and north Asia (see Supplementary Table 1, for details of the cultural background of participants).

Fasting insulin concentration were not significantly different between the two groups. Mean fasting blood glucose was greater in the older (5.17 mmol/L) than the younger (4.77 mmol/L) group (*p* < 0.001). The older group had higher mean blood pressure than the younger group but this was only statistically significant for systolic blood pressure (149 mmHg vs 120 mmHg, *p* < 0.001). A total of 29 people in the older group and nine in the younger group met guideline criteria for the diagnosis of hypertension from the measurements taken. These rates are on par with the levels in the Australian adult population^29^. Older people had a greater mean BMI than younger people (25.6 vs 24.2 kg/m^2^) but this difference was not statistically significant (*p* = 0.133). Seven participants (9%) would meet the definition for obesity with a BMI above 30, including two younger and four older adults. This 9% in our sample is below the 32% of the Australian adult population with a BMI in the obese range^29^.

The average number of medications taken in the last 24 hours was higher in the older (1.3) than the younger (0.28) group (*p* < 0.001; see Supplementary Table 2 for medication classes). There were no significant differences between the age groups in terms of smoking and illicit drug use. However, a higher percentage of older (25%) than younger (9%) adults reported drinking alcohol everyday (*p* = 0.032).

In the following sections, we report the analyses for our primary hypotheses. Given the potential impact of the demographic variables on brain function, we also report general liner models (GLMs) in the Supplement predicting CMR_GLC_, and cognition with the other demographics included as covariates. We also report results of analyses using fasting blood glucose rather than HOMA-IR as a predictor of regional CMR_GLC_ and cognition in the Supplement.

#### Age and insulin resistance and cortical thickness

Two participants in the older group (3% of the whole sample) would meet the Australia Diabetes Society criteria for prediabetes, i.e., a fasting blood glucose of 6.1–6.9. Three percent in our sample is on par with data reported for the adult Australian population^30^. Measures of insulin resistance (HOMA-IR and HOMA-IR2) were greater in the younger group than the older group but these differences were not statistically significant (Table 1). Four people in the younger age group and five in the older age group had HOMA-IR levels greater than 1.8. One person in the younger group and four people in the older group had HOMA-IR levels greater than 2.7. These thresholds of 1.8 and 2.7 have previously been correlated with clinical levels of insulin resistance and other cardiometabolic risk factors^31^. At a HOMA-IR threshold of 2.7, the incidence of insulin resistance in our sample (6%) is consistent with the 5% of adults in the Australian adult population diagnosed with type 2 diabetes^29^.

The older adult group had lower whole brain cortical thickness than the younger group (*p* < 0.001). Older people also had lower cortical thickness than those in the younger group in 94 of 100 regions (Supplementary Tables 3 and 4).

#### Age, cerebral cortical thickness and metabolic rate of glucose

**Fig. 1:**
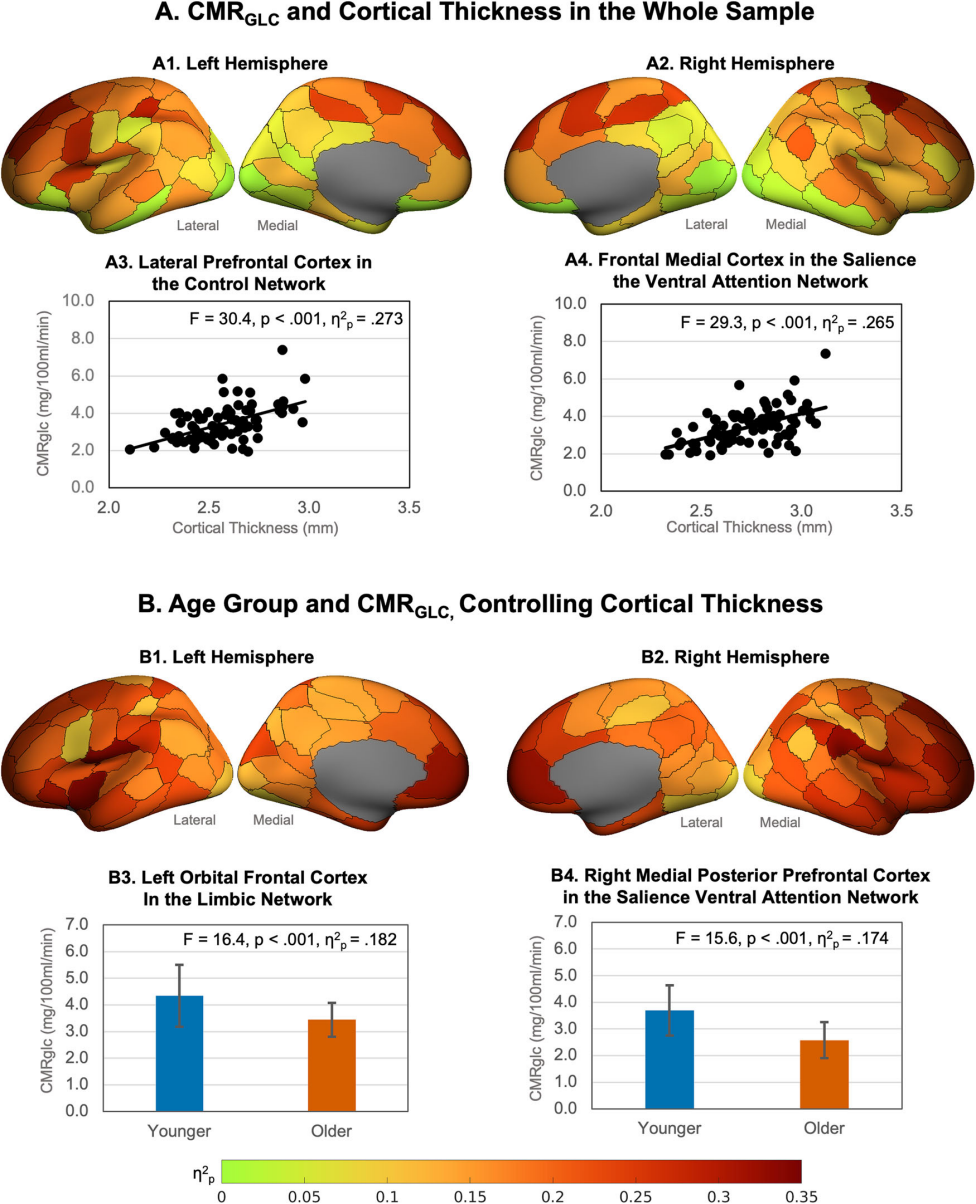
Association between age, cortical thickness and CMR_GLC_. **A** Effect sizes (η^2^_p_) of higher regional CMR_GLC_ associations with higher cortical thickness in the whole sample in left (A1) and right (A2) hemispheres (data is from Supplementary Table 1). Example associations in (A3) the lateral prefrontal cortex in the control network, and (A4) the frontal medial cortex in the salience ventral attention network. **B** Association between age group and CMR_GLC_ controlling for cortical thickness in the left (B1) and right (B2) hemispheres. Example age group and CMR_GLC_ relationships in (B3) the left orbital frontal cortex in the limbic network, and (B4) the right medial posterior prefrontal cortex in the salience ventral attention network (bars are group means and error bars are standard deviations; data is from Supplementary Tables 1 and S2). Figures were produced using the toolbox at: https://github.com/StuartJO/plotSurfaceROIBoundary.

In the whole sample, greater cortical thickness was associated with greater regional CMR_GLC_ in 83 of the 100 regions with the largest effect sizes in the superior, middle and medial frontoparietal cortices (Fig. 1A, and Supplementary Table 4). Taking the association between age and cortical thickness into account, those in the older group had lower CMR_GLC_ than those in the younger group in all regions (Fig. 1B and Supplementary Table 5). The effect sizes ranged from 0.20 to 0.24 in regions in the somatomotor, salience ventral attention, control and default networks, to less than 0.10 mostly in regions in the visual network and sub-cortical structures. The largest effects were in the medial, ventral and dorsal prefrontal cortices in the default network; the lateral prefrontal cortex, insula and the parietal operculum and lobule in the salience ventral attention network; the lateral prefrontal and temporal cortices in the control network; and regions of the somatomotor network.

### Age, Insulin resistance and cerebral metabolic rate of glucose

**Fig. 2:**
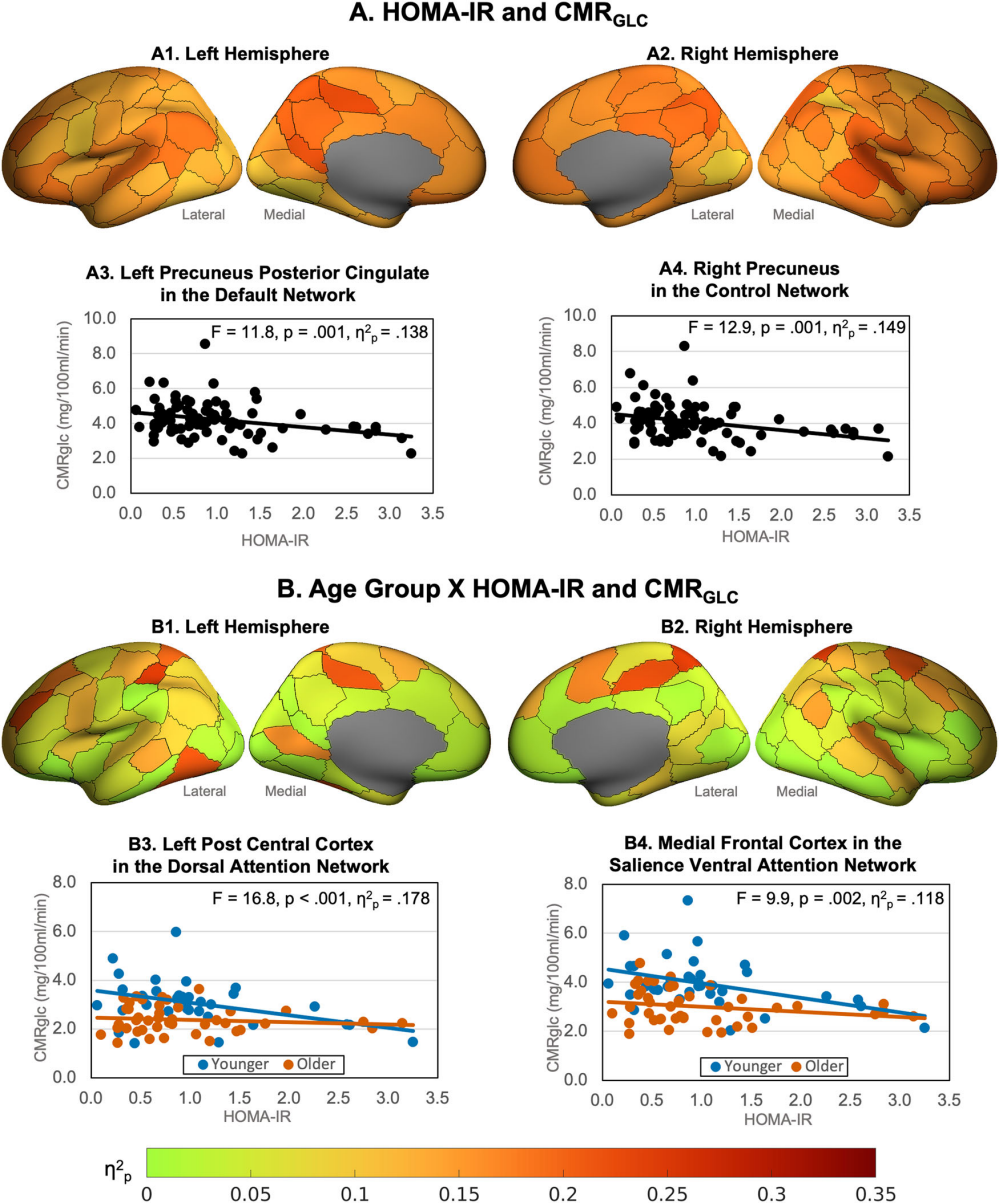
Association of HOMA-IR and HOMA-IR and age interaction with CMR _GLC_. Effect sizes (η^2^_p_) of regional CMR_GLC_ associations with (**A**) HOMA-IR and (**B**) age group x HOMA-IR, controlling for cortical thickness (data in Supplementary Table 3). Example HOMA-IR effects in (A3) the left precuneus posterior cingulate in the default network and (A4) right precuneus In the control network. Example age group X HOMA-IR interactions in (B3) left post central cortex in the dorsal attention network and (B4) right medial frontal cortex in the salience ventral attention network.

Greater HOMA-IR was associated with lower CMR_GLC_ across all regions (Fig. 2A and Supplementary Table 5). The effect sizes ranged from 0.14 to 0.16 in regions in the salience ventral attention, somatomotor, default and control networks, to less than .08 in regions in the visual and control networks and the sub-cortical structures. The largest effects of HOMA-IR were in the lateral prefrontal, medial parietal and parietal operculum in the salience ventral attention network; the ventral and dorsal prefrontal cortices, parietal lobule and posterior cingulate in the default network; the lateral prefrontal cortex, precuneus and cingulate in the control network; and several somatomotor regions.

We found a statistically significant interaction between the older and younger groups and HOMA-IR levels on CMR_GLC_ in 41 regions (Fig. 2B; Supplementary Table 6). In post-hoc tests we found that greater insulin resistance was associated with lower CMR_GLC_ in the younger group but not the older group. The nature of this interaction was such that a 10% increase in HOMA-IR in the younger group was associated with a lower CMR_GLC_ across regions of between 3.3% and 7.3%. In the older group a 10% increase in HOMA-IR was not statistically associated with a change in CMR_GLC_ (zero to −2.2%). The largest reductions for younger adults were in the dorsal prefrontal cortex and parietal medial regions of the default network; the lateral prefrontal cortices in the control and salience ventral attention networks; the medial frontal cortex in the default network; the superior parietal and post central regions of the dorsal attention network; the temporal parietal cortex and insula; and regions of the somatomotor network.

We also found statistically significant age group x HOMA-IR interactions in all networks (Supplementary Table 7). The nature of these interactions was similar to those at the regional level in that greater HOMA-IR was associated with lower network CMR_GLC_ in the younger but not the older group. Large effect sizes (η^2^_p_ > 0.20) of HOMA-IR were found for younger adults in the control, salience ventral attention, somatomotor, default and visual networks.

### Cerebral metabolic rates of glucose and cognition

Five principal components (PCs) of cognition with eigenvalues greater than one were identified across the six cognitive tests and eleven measures, explaining 81% of the variance. The varimax rotation converged in five iterations (see Supplementary Table 8). Cognitive control (task-switching and WASI FSIQ) loaded most strongly on the first principal component. Visuospatial processing speed (digit substitution) loaded most strongly on the second principal component; response inhibition (stop-signal) on the third component; working memory (digit span) on the fourth component; and verbal learning and memory (HVLT) on and the fifth component.

**Fig. 3:**
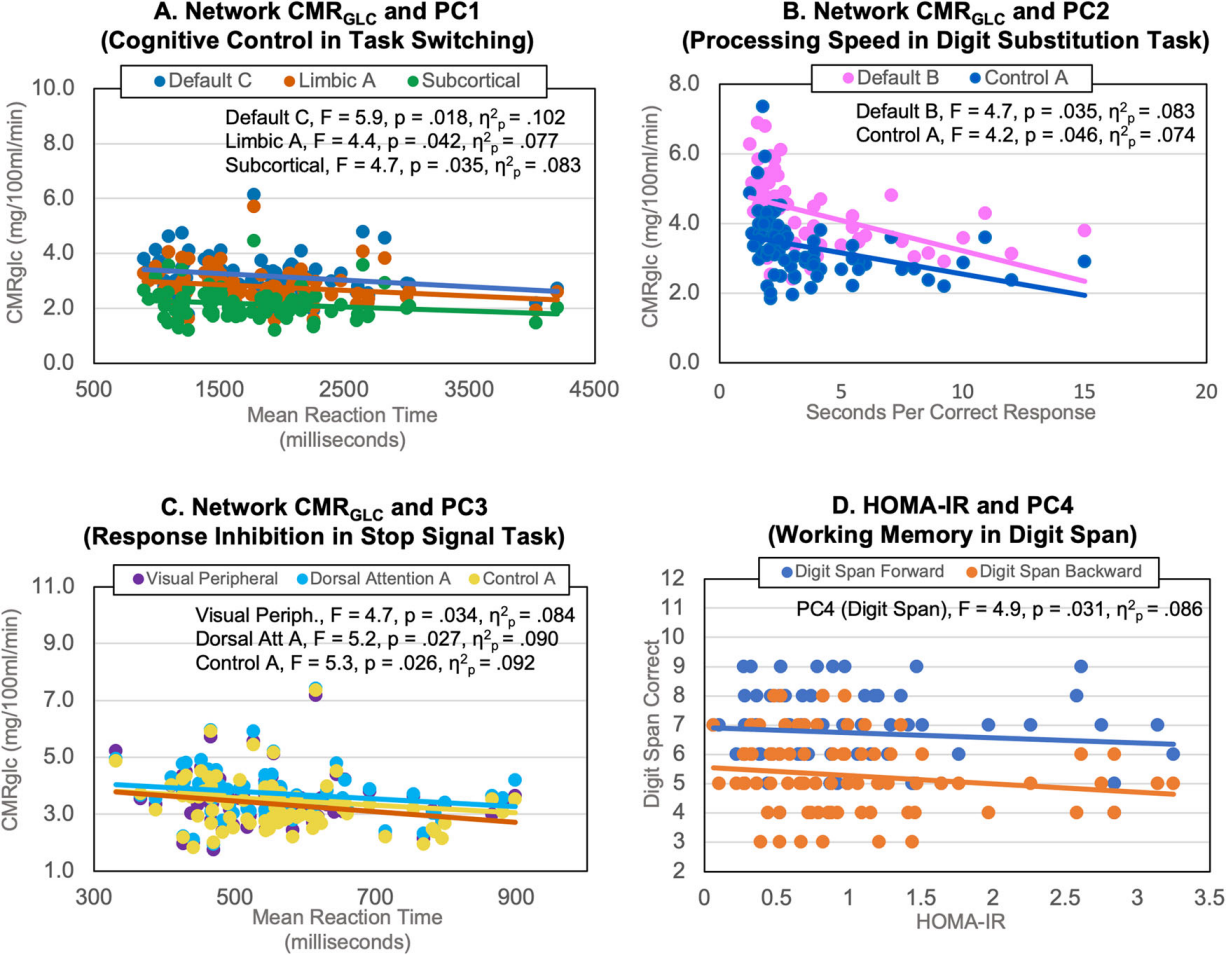
Relationship between network CMR_GLC_ and principal components (PC) of cognition to illustrate significant effects from the GLMs (Table S6). Significant negative associations of higher network CMR_GLC_ and: **A** shorter mean reaction time in task switching trials (loading on PC1), **B** lower processing speed in the digit substitution task (PC2) and **C** shorter mean reaction time in stop signal trials measuring response inhibition (PC3). **D** Significant negative association of higher HOMA-IR and lower longest forward and backward digit span performance (PC4).

Higher CMR_GLC_ in the limbic, default and subcortical networks was associated with better performance on cognitive control (PC1, see Supplementary Table 9). In particular, higher network CMR_GLC_ was associated with a faster reaction time in task-switching (see Fig. 3A). Higher CMR_GLC_ in the control and default networks was associated with faster visuospatial processing speed (PC2, digit substitution; Fig. 3B). Higher CMR_GLC_ in the dorsal attention and control networks was associated with better response inhibition (PC3, stop signal; Fig. 3C). Interestingly, lower CMR_GLC_ was associated with higher WASI FSIQ2 and number of correct responses in the digit substitution task.

Older adults had slower reaction time than younger adults in task switching but higher WASI FSIQ (PC1; see Supplementary Table 9). Older adults also had slower visuospatial processing speed in the digit substitution task (PC2). Higher HOMA-IR had a direct effect on worse working memory (PC 4, digit span, Fig. 3D). However, none of the interaction terms of HOMA-IR and network CMR_GLC_ were significant in the stepwise regression models, indicating that levels of HOMA-IR did not moderate the relationship between CMR_GLC_ and cognition.

## Discussion

### The association between CMR_GLC_ and age, HOMA-IR and cortical thickness

As expected, we found that older adults had lower cortical thickness than younger adults. However, unexpectedly, older adults in our sample were not more insulin resistant than younger adults. We also found that greater age and greater insulin resistance were associated with lower cerebral glucose metabolism across the brain, particularly in prefrontal and temporal cortices. Although we found that greater age was associated with lower cortical thickness and that lower cortical thickness was associated with lower cerebral glucose metabolism, the association between older age and lower cerebral glucose metabolism remained after adjusting for differences in cortical thickness. These results are consistent with previous primary research and a recently published systematic review and meta-analysis^25^. They suggest that older adults have less metabolic active cortical tissue than younger adults but they also have less efficient metabolism of glucose on a per gram tissue basis. Our results are also consistent with research in older adults in which higher HOMA-IR was associated with lower glucose metabolism in regions of the medial orbital, prefrontal and temporal cortices and the cingulate and insula^22,32^.

Consistent with our hypothesis, we found that the association between insulin resistance and cerebral glucose metabolism varied by age group in 41 regions, particularly in regions in the prefrontal, parietal, temporal and somatomotor cortices. However, in an unexpected finding, the association between greater insulin resistance and lower cerebral glucose metabolism was seen in the younger but not older adults. Rates of cerebral metabolism in insulin resistant younger adults were also lower in all networks than in their more insulin sensitive counterparts, particularly in the control, salience ventral attention, somatomotor and default mode networks. Previous research has also shown a negative effect from metabolic dysfunction on grey matter volume, white matter integrity and cognition in people in their late 20 s, 30 s and 40 s^18–20^. Our results add to this research by suggesting that cerebral metabolism is also attenuated by insulin resistance in otherwise healthy younger adults.

It is striking that insulin resistance was associated with significant reductions in regional CMR_GLC_ in younger but not older adults. This is somewhat counterintuitive, as we had expected that insulin resistance would be related to CMR_GLC_ reductions more so in older adults. The lack of an association of insulin resistance and CMR_GLC_ in our older adult group may reflect insulin resistance already having impacted metabolic brain function. In other words, the lower absolute rates of CMR_GLC_ and lack of a HOMA-IR association in the older adult group could reflect accumulated effects of insulin resistance that are not yet occurring in younger adults.

Our older adult results are also different to the one other study by Nugent et al.^32^ that also included younger adults, in which HOMA-IR did not correlate with CMR_GLC_ in any brain region. One possible reason for these different results is that our younger sample had a broader age range by 12 years and slightly higher HOMA-IR2 than Nugent et al. (20–42 years vs 18–30 years; HOMA-IR2 0.6 vs 0.5). We also note that our older adult group had slightly lower mean and standard deviation fasting insulin and HOMA-IR levels than our younger adult group (see Table 1). The lower HOMA-IR in our older adult group is in contrast to the association between ageing and increasing insulin resistance shown in studies of the general population^33,34^. We do not rule out the possibility of an association between greater insulin resistance and lower cerebral glucose metabolism in other adult samples. Such patterns have been previously reported in other studies of older adults and we add to this work by suggesting that greater insulin resistance and lower cerebral glucose metabolism is seen in younger samples than previously examined. Replicating and extending our findings, including a mid-life group, and older adults with a wider range of insulin resistance levels, is warranted.

We also explored the association between fasting blood glucose and regional CMR_GLC_ (see Supplementary Section 7). Whereas higher HOMA-IR was associated with lower CMR_GLC_ for younger but not older adults, higher fasting blood glucose was associated with lower CMR_GLC_ for older adults only. These results suggest that insulin resistance and fasting blood glucose have unique associations with cerebral metabolism in ageing. We note that fasting blood glucose levels were also significantly higher for older than younger adults. Our results extend research showing that the degree of hyperglycaemia is correlated with the risk of neurological complications in people with diabetes^35,36^ by indicating that glycaemia is also associated with changes in cerebral metabolism in otherwise healthy older adults. Hyperglycaemia and glucose intolerance in older adults have been linked to reduced β-cell insulin secretory capacity and loss of peripheral tissue sensitivity to insulin and have been attributed to lifestyle (e.g., physical inactivity) and comorbidity-related risk factors (e.g., adiposity)^37^. Further research is needed to examine these mechanisms in normal ageing and their associations with brain health.

### CMR_GLC_ primarily in ‘higher order’ brain networks is associated with cognition

Consistent with our hypothesis, higher regional CMR_GLC_ in the default, control, dorsal attention, limbic and subcortical networks was associated with faster visuospatial processing speed and reaction time in the digit substitution, task switching and stop signal tasks (see Fig. 3). However, these associations were not moderated by levels of peripheral insulin resistance. The networks in which higher CMR_GLC_ was associated with faster visuospatial processing speed and reaction time reflect mostly ‘higher order’ (e.g., control and attention) as opposed to primary sensory networks (e.g., visual). These results suggest that higher rates of glucose metabolism in the ‘higher order’ networks supports flexible, adaptive responses in goal-directed behaviour, as well as inhibition of inappropriate actions. These results also likely reflect the fact that the cognitive battery primarily indexed memory, visuospatial processing and attentional control.

Unexpectedly, higher CMR_GLC_ was associated with lower WASI scores. WASI, which is an estimate of full scale IQ (FSIQ), was acquired as a more robust estimate of cognitive reserve, which is often measured using proxy indices such as reading tests or educational attainment^38^. Our sample had a relatively high mean WASI FSIQ2 of 116 and seven participants scored above 140, all in the older age group, indicating high levels of cognitive reserve. Two younger participants scored above 130. Together these results suggests that reduced rates of cerebral glucose in the brain of adults primarily attenuate processing speed rather than task accuracy, even in older adults who retain relatively high cognitive reserve.

We did not find significant network CMR_GLC_ associations with episodic and working memory (PC4 and PC5). However, we did find higher HOMA-IR to be associated with worse working memory, an effect also found in previous research^39,40^, suggesting that insulin resistance is a risk factor for working memory impairment. Unexpectedly, we did not find age group differences for episodic and working memory. A large body of research has shown that older adults typically show a decline in episodic and working memory compared with younger adults (see Refs. ^41,42^), further highlighting that our sample of older adults were particularly cognitively-healthy with high cognitive reserve. Additional research is warranted to investigate how closely cerebral glucose metabolism and insulin are coupled with cognition across the adult lifespan.

### Possible mechanisms and future directions

The mechanisms through which insulin resistance leads to changes in brain function remain to be fully understood. However, a growing body of research suggests that the brain is sensitive to the levels of peripheral insulin and uses insulin in a range of functions^43–45^. Research also suggesting shared pathways or mechanisms driving changes to the brain in ageing and insulin resistance^8^. The mechanism include metabolic disturbances from an increase in neuronal insulin resistance, decreased brain insulin receptor number and function, impaired insulin signalling, a pro-inflammatory state and mitochondrial dysfunction^46,47^. Prolonged peripheral hyperinsulinemia can also decrease insulin receptors at the blood-brain barrier, thereby reducing insulin transport into the brain^46^. Neurons in the hypothalamus and brainstem that are responsible for energy homoestatis and feeding are also impaired in insulin resistance^2^. Much of the research in this area has been in people with a diagnosis of insulin resistance or type 2 diabetes^7^ or has been limited to older participants^6,22,24^. However, our results indicate that variability in peripheral insulin resistance within a healthy range is also associated with changes in cerebral metabolism, particularly in younger adults.

Our study sample had some similar health and demographic characteristics to the Australian adult population, including similar rates of high blood pressure and fasting glucose and HOMA-IR in the prediabetic and diabetic range. However, our sample also had a lower rate of BMI in the obesity range, slightly more years of education, and the older adult group had high cognitive reserve, as indexed by IQ. A high proportion of the younger adult group was born overseas, particularly from Asian backgrounds. Although we assessed the effects of the demographic variables on CMR_GLC_ and cognition and found them to add minimal predictive power beyond age and HOMA-IR, additional research is needed to replicate our findings in other populations across a range of education and cardiometabolic health levels, and cultural backgrounds.

The current study also used a cross-sectional design, limiting conclusions about any causal relationships. The differences we found between groups may also reflect underlying cohort differences rather than age-related changes. Longitudinal research could test whether changes in insulin function in early adulthood not only have a proximal effect, such as those reported here, but also moderate or even accelerate cerebral metabolic changes in ageing. Our study is also limited by the absence of middle aged adults. Research on structural and functional brain networks have reported quadratic trajectories of age differences, with an inflection point somewhere in the third to fifth decade of life (see Ref. ^48^ for review). However, the lack of middle aged adults precludes the identification and quantification of ageing trajectories across the full adult lifespan. Additional research is needed to elucidate these patterns.

HOMA-IR is considered a reliable clinical and research tool for the assessment of levels of insulin resistance. Nevertheless, a limitation of HOMA-IR is that it represents a single snapshot of the complex glucose-insulin system^49^. Research using dynamic measures (e.g., hyperinsulinemic-euglycemic clamp or 2-hour glucose tolerance tests) could improve our understanding of the complexity of insulin signalling and metabolism in the periphery and the brain in ageing and across the spectrum of health and metabolic-related diseases.

### Implications for the maintenance of brain health across the adult lifespan

The results of the current study suggest that insulin resistance may contribute to brain health even in younger adults. Diet, lifestyle (e.g., sleep, exercise, stress) and genetics are risk factors for an increase in insulin resistance and would appear to be targets for public health interventions and clinical application to optimise both peripheral and central glucose metabolism^2,50^. Pharmaceutical treatments for diabetes that target peripheral glucose and bodyweight reductions may reduce the risk for cognitive decline^51,52^. Medications that increase sensitivity to insulin have been used for years in people with diabetes and are now being considered in people without diabetes to improve brain health. Our work suggests that future research should consider including people in early adulthood given the signals between insulin resistance and cerebral glucose metabolism we report here.

## Methods

This study design, hypotheses and analyses were preregistered at OSF registrations (https://osf.io/93mnd).

### Ethical considerations

The study protocol was reviewed and approved by the Monash University Human Research Ethics Committee in accordance with Australian Code for the Responsible Conduct of Research (2007) and the Australian National Statement on Ethical Conduct in Human Research (2007). Administration of ionizing radiation was approved by the Monash Health Principal Medical Physicist, following the Australian Radiation Protection and Nuclear Safety Agency Code of Practice (2005). For participants older than 18 years, the annual radiation exposure limit of 5 mSv applies. The effective dose in this study was 4.9 mSv. Participants provided informed consent to participate in the study.

### Participants

Ninety participants were recruited from the general community via local advertising. An initial screening interview ensured that participants had the capacity to provide informed consent, did not have a diagnosis of diabetes, neurological or psychiatric illness. Participants were also screened for claustrophobia, non-MR compatible implants, and clinical or research PET scan in the past 12 months. Women were screened for current or suspected pregnancy. Participants received a $100 voucher for participating in the study. Eleven participants were excluded from further analyses due to blood haemolysis or well counter issues preventing insulin measurement or kinetic modelling (*N* = 7), excessive head motion (*N* = 2) or incomplete PET scan or image reconstruction (*N* = 2). The final sample included 79 individuals, 36 younger (mean 27.8; SD 6.2; range 20–42 years) and 43 older (mean 75.5; SD 5.8; range 66–86 years) adults (see Table 1). Exclusion criteria included a known diagnosis or history of diabetes reported by participants at the time of recruitment to the study.

### Data acquisition

#### Cognitive battery

Participants completed an online demographic and lifestyle questionnaire and a cognitive test battery. The following cognitive measure were used:

##### Wechsler abbreviated scale of intelligence (WASI-IQ)

An assessment of intelligence suitable for ages 6–90 years^53^. There are 4 subtests: block design, vocabulary, matrix reasoning and similarities. WASI-IQ was scored by converting raw scores into a scale score, which were transformed into a composite score reflecting verbal comprehension and perceptual reasoning abilities (FSIQ2). This score was converted to an age-based T scores established in a normal population.

##### Hopkins verbal learning test (HVLT)

A three-trial list learning and free recall task comprising 12 words, four words from each of three semantic categories^54^. Approximately 20–25 minutes later, a delayed recall trial and a recognition trial was completed. The delayed recall required free recall of any words remembered. The recognition trial comprised 24 words, including the 12 target words and 12 false-positives, six semantically related, and six semantically unrelated. Delayed recall (total words recalled) and a recognition discrimination index (number of correct minus number of false positives in the recognition task) were calculated.

##### Digit span

A measure of verbal short term and working memory used in two formats: Forward and backward digit span^55^. Participants were presented with a series of digits, and are asked to repeat them in either the order presented (forward span) or in reverse order (backwards span). After two consecutive failures of the same length, the test was stopped. Scores were derived as the length of longest correct series for both forward and backward recall.

##### Task switching

A computer-based test in which participants were given a word and had to perform one of two simple categorisation tasks, depending on the cue that appeared with the word: (1) ‘living’ task. If the cue was a heart, participants were asked to categorise the word via a key press based on whether it represents a LIVING versus a NON-LIVING object; and (2) ‘size’ task. If the cue was an arrow-cross, participants were asked to categorise the word via a key press based on whether it represents an object that is BIGGER or SMALLER than a basketball. The cue selection for each new trial was randomised. Half the test trials were switch trials; half non-switch trials. Half the switch and non-switch trials was congruent in the key presses for either task, half was incongruent. The measures used included the percentage of correct switch trials and mean latency of correctly responding to a switch trial^56^.

##### Stop signal

A computer-based test in which participants were presented an arrow that pointed either right or left^57^. The task was to press the left response key if the arrow pointed to the left and press the right response key if the arrow pointed to the right, unless a signal beep was played after the presentation of the arrow. In this case the response should be stopped before execution. The delay between presentation of arrow and signal beep (starting at 250 ms) was adjusted up or down (by 50 ms) depending on performance. The delay increased if the previous signal stop was successful (up to 1150 ms) and decreased if the previous signal stop was not successful (down to 50 ms). The stimulus onset asynchrony between the start of each trial (onset of fixation circles) was 2000 ms. Variables were the mean reaction time in stop signal trials and stop signal reaction time. Stop signal reaction time is an estimate of inhibition ability, that is, the time required to stop the initiated go-process. The slower the stop signal reaction time, the more difficult to stop the go-process.

##### Digit symbol substitution

A computer-based task in which participant were presented with an 18 column × 16 row matrix^58^. The task was to translate symbols shown above the matrix (key) into digits in the matrix within a two minute period. Total count of correct responses and seconds per correct response were recorded.

#### MR-PET data acquisition

Participants underwent a 90-minute simultaneous MR-PET scan in a Siemens (Erlangen) Biograph 3-Tesla molecular MR scanner. Participants were directed to consume a high-protein/low-sugar diet for the 24 hours prior to the scan. They were also instructed to fast for six hours and to drink 2–6 glasses of water. Prior to FDG infusion, participants were cannulated in the vein in each forearm and a 10 ml baseline blood sample taken. At the beginning of the scan, half of the 260 MBq FDG tracer was administered via the left forearm as a bolus, providing a strong PET signal from the beginning of the scan. The remaining 130 MBq of the FDG tracer dose was infused at a rate of 36 ml/hour over 50 minutes, minimising the amount of signal decay over the course of the data acquisition. We have previously demonstrated that this protocol provides a good balance between a fast increase in signal-to-noise ratio at the start of the scan, and maintenance of signal-to-noise ratio over the duration of the scan^59^.

Participants were positioned supine in the scanner bore with their head in a 32-channel radiofrequency head coil and were instructed to lie as still as possible. The scan sequence was as follows. Non-functional MRI scans were acquired during the first 12 minutes, including a T1 3DMPRAGE (TA = 3.49 min, TR = 1640 ms, TE = 234 ms, flip angle = 8°, field of view = 256 × 256 mm^2^, voxel size = 1.0 × 1.0 × 1.0 mm^3^, 176 slices, sagittal acquisition) and T2 FLAIR (TA = 5.52 min, TR = 5000 ms, TE = 396 ms, field of view = 250 × 250 mm^2^, voxel size = 0.5 × 0.5 × 1 mm^3^, 160 slices) to image the anatomical grey and white matter structures, respectively. Thirteen minutes into the scan, list-mode PET (voxel size = 1.39 × 1.39 × 5.0 mm^3^) and T2* EPI BOLD-fMRI (TA = 40 minutes; TR = 1000 ms, TE = 39 ms, FOV = 210 mm2, 2.4 × 2.4 × 2.4 mm^3^ voxels, 64 slices, ascending axial acquisition) sequences were initiated. A 40-minute resting-state scan was undertaken in naturalistic viewing conditions watching a movie of a drone flying over the Hawaii Islands. At 53 minutes, pseudo-continuous arterial spin labelling (pc-ASL) began, and at 58 minutes, diffusion-weighted imaging (DWI) was acquired with 71 directions to index white matter connectivity. pcASL, DWI and fMRI results are not reported here.

Plasma radioactivity levels were measured throughout the scan. Beginning at 10-minutes post infusion onset, 5 ml blood samples were taken from the right forearm using a vacutainer at 10-minute intervals for a total of nine samples. The blood sample were immediately placed in a Heraeus Megafuge 16 centrifuge (ThermoFisher Scientific, Osterode, Germany) and spun at 2000 rpm (RCF ~ 515 g) for 5 minutes. 1000-μL plasma was pipetted, transferred to a counting tube, and placed in a well counter for four minutes. The count start time, total number of counts, and counts per minute were recorded for each sample.

### MRI pre-processing and cortical thickness

For the T1 images, the brain was extracted in Freesurfer; quality of the pial/white matter surface was manually checked, corrected and registered to MNI152 space using Advanced Normalization Tools (ANTs). Cortical thickness for the Schaefer 100 regions was obtained from the Freesurfer reconstruction statistics for each participant.

### PET image reconstruction and pre-processing

The list-mode PET data for each subject were binned into 344 3D sinogram frames of 16 s intervals. Attenuation was corrected via the pseudo-CT method for hybrid PET-MR scanners^60^. Ordinary Poisson-Ordered Subset Expectation Maximization algorithm (3 iterations, 21 subsets) with point spread function correction was used to reconstruct 3D volumes from the sinogram frames. The reconstructed DICOM slices were converted to NIFTI format with size 344 × 344 × 127 (voxel size: 1.39 × 1.39 × 2.03 mm^3^) for each volume. All 3D volumes were temporally concatenated to form a single 4D NIFTI volume. After concatenation, the PET volumes were motion corrected using FSL MCFLIRT^61^, with the mean PET image used to mask the 4D data.

### Correction for partial volume effects

PET images were corrected for partial volume effects using the modified Müller-Gartner method implemented in PetSurf (https://surfer.nmr.mgh.harvard.edu/fswiki/PetSurfer)^62,63^. A grey matter threshold of 20–30% is recommended in ageing because atrophy can influence results^62^. For our analyses, we chose a 25% grey matter threshold and surface-based spatial smoothing^63^. We used a Gaussian kernel with a full width at half maximum of 12 mm to increase the signal-to-noise ratio. Subcortical structures were partial volume corrected and spatially smoothed in volume space and merged with the cortical data.

### Cerebral metabolic rates of glucose

Calculations of regional CMR_GLC_ were undertaken in PMOD 4.4 (http://www.pmod.com) using the FDG time activity curves for the Schaefer 100 atlas parcellation and AAL subcortical structures. The FDG in the plasma samples was decay-corrected for the time between sampling and counting, and used as the input function to Patlak models. A lumped constant of 0.89 was used^64^, and equilibrium (t) set at 10 mins, the time corresponding to the peak of the bolus and onset of a stable signal^65^. The fractional blood space (vB) was set at 0.05^66^. Participant’s plasma glucose (mmol) was entered in the model from their baseline blood sample.

CMR_GLC_ in the 17 networks was calculated from the regional CMR_GLC_ values for each participant. Because the regions within a network differ in cortical volume, the regional CMR_GLC_ values could not simply be averaged. Rather, each regional CMR_GLC_ value was weighted by the percentage its volume represented within the total network cortical volume. An overall subcortical CMR_GLC_ value was calculated by weighting each structure by the percentage its volume represented from the total volume of the subcortical structures.

### HOMA-IR

A blood sample taken prior to FDG infusion was used to collect 2 ml of plasma for insulin and glucose measurement, which was undertaken by a commercial laboratory. HOMA-IR was calculated as fasting glucose (mmol/L) x fasting insulin (µU/ml) / 22.5^67^. The constant of 22.5 is a normalising factor for normal fasting plasma insulin and glucose (i.e., 4.5 mmol/L x 5 μU/ml = 22.5). Higher HOMA-IR values indicate greater insulin resistance. We also calculated HOMA-IR2 (https://www.rdm.ox.ac.uk/). We compared the relationship between HOMA-IR and HOMA-IR2 with CMR_GLC_ and found minimal to no differences (see Supplementary Tables 5 and S11). This was expected as HOMA-IR2 models increases in the insulin secretion curve for plasma glucose concentrations above 10 mmol/L^68^; a threshold that than none of our participants reached. Hence, the results reported here are based on HOMA-IR.

### Data analysis

The CMR_GLC_ and HOMA-IR data was inspected for and found to be satisfactory for assumptions of normality and potential impact of any outliers (see Supplementary Fig. 1).

#### Age, cortical thickness, HOMA-IR and CMR_GLC_

*Hypothesis 1:* Independent sample T-tests were run to test hypothesis 1 that older people would have greater insulin resistance and lower cortical thickness than younger people.

*Hypothesis 2 and 3:* A series of general linear models (GLMs) was run in which regional CMR_GLC_ was the dependent variable and age group, HOMA-IR and age group x HOMA-IR were the predictors. Cortical thickness in the same region was included as a covariate. For the subcortical structures, whole brain average cortical thickness was used as the covariate. The age group main effect was used to assess hypothesis 2 that older people would have lower regional cerebral metabolic rates of glucose than younger people, even after adjusting for lower cortical thickness in older people. The age group x HOMA-IR effects were used to test hypothesis 3 that greater insulin resistance would be associated with lower cerebral metabolic rate of glucose and that this association would be moderated by age, with the effect being stronger in older adults. For significant age group x HOMA-IR effects, post-hoc GLMs were run separately for younger and older adults. A series of GLMs was also run for CMR_GLC_ at the 17 network level with the same design.

Partial eta squared (η^2^_p_) was used to quantify the effect sizes in the GLMs. Each series of analyses was also FDR-corrected at *p* < 0.05 for the overall GLM. We also calculated the percentage change in regional CMR_GCL_ from a 10% change in HOMA-IR from the slope of the regression lines for younger and older adults separately.

#### CMR_GLC_ and cognition

*Hypothesis 4:* We applied data reduction techniques to reduce the dimensions in the cognitive test data. The cognitive scores were converted to Z-scores and entered in a Principal Component Analysis (PCA). Principal components (PCs) with eigenvalues greater than one were retained and subject to varimax rotation to optimally reduce dimensionality^69^. Participant component scores were saved for further analyses.

A series of GLMs was run to test hypothesis 4 that greater cerebral metabolic rates of glucose and lower insulin resistance would be associated with better cognitive test performance. The five cognition PCs were entered as the dependent variable, with CMR_GLC_ in the 17 networks entered as independent variables, together with age group, whole brain cortical thickness and HOMA-IR. To test for a moderating effect of HOMA-IR, where a network CMR_GLC_ predicted a principal component of cognition, a product term was created between CMR_GLC_ and HOMA-IR. Stepwise regression was used with the significant CMR_GLC_ network(s) entered in block 1, and the product term(s) with HOMA-IR in block 2. A significant increase in variance explained from block 1 to block 2 was indicative of moderation.

## Supplementary Information

Supplementary Information

## Data Availability

The datasets used and/or analysed in the current study are available from the corresponding author on reasonable request.
