# Supplementary material for: Peripheral insulin resistance attenuates cerebral glucose metabolism and impairs working memory in healthy adults
Source: NPJ Metab Health Dis. 2024 Aug 2;2:17. doi: 10.1038/s44324-024-00019-0 (PMC12118730; doi:10.1038/s44324-024-00019-0)
Supplement: Supplementary file 1 — Supplementary Information [file 44324_2024_19_MOESM1_ESM.pdf]

Peripheral insulin resistance attenuates cerebral glucose metabolism and impairs working memory in healthy adults

Deery, Liang, Di Paolo, Voigt, Murray, Siddiqui, Egan, Moran, Jamadar

~ Supplementary Information ~

TABLE OF CONTENTS

Supplementary Results 2

1. Additional Demographic Details: Cultural Background and Medication Use 2
2. Regional CMR_{GLC} for younger and older adults 4
3. Normality and Outlier Considerations 5
4. Cortical Thickness, Age, HOMA-IR and CMR_{GLC} 7
5. Principal Component and GLM Analyses for Cognitive Variables 12
6. Effects of demographic variables and HOMA-IR2 on CMR_{GLU}, Cortical Thickness and Cognition . 14
7. Association of Fasting Blood Glucose with regional CMR_{GLU}, and Cognition 19

Supplementary Results

1. *Additional Demographic Details: Cultural Background and Medication Use*

Participants self-reported their cultural background (Supplementary Table 1). For the whole sample, 56% of participants identified as Australian, 13% as South-East Asian and 10% as Southern and Central Asian. The percentage of participants identifying as Australian was higher for the older (72%) than the younger (36%) participants (p = .001). In contrast, a higher percentage of younger adults identified as South-East Asian and Sothern and Central Asian (25% and 22%) than older adults (2% and 0%) (p = .003 and p = .001).

For medication use (Supplementary Table 2), statins were the most frequently reported medication among the whole sample (10%), followed by proton pump inhibitors and paracetamol (both 6%). For those medications, the usage was significantly higher among older than younger adults (all p< .05). The next most common medications were ACE inhibitors, angiotensin receptor blockers, beta-blockers and anti-inflammatory medications (all 5%). Although these were also used more frequently among older adults, the differences were not statistically significant.

Supplementary Table 1. Number and percent of participants identifying with major cultural backgrounds in the whole sample and for younger and older adults. Chi-squared test of differences between younger and older groups.

	Whole Sample		Younger		Older		Younger Vs older	
	Number	Percent	Number	Percent	Number	Percent	Chisquare	p
Australian	44	56%	13	36%	31	72%	10.3	0.001
New Zealander	1	1%	0	0%	1	2%	0.8	0.357
Southern and Eastern European (Southern European, South-Eastern European)	2	3%	1	3%	1	2%	0.0	0.899
North African and Middle Eastern (Arab, Jewish, Sudan, Other North African and Middle Eastern)	3	4%	2	6%	1	2%	0.6	0.454
South-East Asian (Mainland South-East Asian, Maritime South-East Asian)	10	13%	9	25%	1	2%	9.1	0.003
North-East Asian (Chinese Asian, Other)	5	6%	5	14%	0	0%	6.4	0.012
Southern and Central Asian (Southern Asian, Central Asian)	8	10%	8	22%	0	0%	10.6	0.001
Americas (North American, South American, Central American, Caribbean Islander)	2	3%	2	6%	0	0%	2.5	0.117
North-West European (British, Irish, Western European, Northern European)	4	5%	0	0%	4	9%	3.5	0.060

Note: participants could report identifying with more than one cultural background.

Supplementary Table 2. Number and percent of medication use in the whole sample and for younger and older adults. Chi-squared test of differences between younger and older groups.

	Whole Sample		Younger		Older		Younger Vs older	
	Number	Percent	Number	Percent	Number	Percent	Chisqure	p
ACE inhibitor	4	5%	0	0%	4	9%	3.5	0.060
Angiotensen receptor blocker	4	5%	0	0%	4	9%	3.5	0.060
Anti-inflammatory	4	5%	2	6%	2	5%	0.0	0.855
Antiarrhythmic	1	1%	0	0%	1	2%	0.8	0.357
Antibiotic	2	3%	1	3%	1	2%	0.0	0.899
Anticoagulant	1	1%	0	0%	1	2%	0.8	0.357
Antidepressant	2	3%	0	0%	2	5%	1.7	0.190
Antifungal	1	1%	0	0%	1	2%	0.8	0.357
Antihistamine	1	1%	1	3%	0	0%	1.2	0.271
Antimalarial	1	1%	1	3%	0	0%	1.2	0.271
Antimetabolite	1	1%	1	3%	0	0%	1.2	0.271
Antiplatelet	2	3%	0	0%	2	5%	1.7	0.190
Antiresorptive	1	1%	0	0%	1	2%	0.8	0.357
Beta blocker	4	5%	0	0%	4	9%	3.5	0.060
Blood pressure other - class not specified	1	1%	0	0%	1	2%	0.8	0.357
Calcium channel blocker	3	4%	0	0%	3	7%	2.6	0.106
Cold and flu - class not specificed	1	1%	1	3%	0	0%	1.2	0.271
Corticosteroid	2	3%	0	0%	2	5%	1.7	0.190
GLP1-agonist	1	1%	1	3%	0	0%	1.2	0.271
HRT	2	3%	0	0%	2	5%	1.7	0.190
NSAID	2	3%	0	0%	2	5%	1.7	0.190
Opiod receptor agonist	2	3%	1	3%	1	2%	0.0	0.899
Oral contraceptive	1	1%	1	3%	0	0%	1.2	0.271
Pancreatic enzymes	1	1%	0	0%	1	2%	0.8	0.357
Paracetamol	5	6%	0	0%	5	12%	4.5	0.035
Proton pumb inhibitor	5	6%	0	0%	5	12%	4.5	0.035
Sleep - unspecififed	1	1%	0	0%	1	2%	0.8	0.357
Statin	8	10%	0	0%	8	19%	7.5	0.006
Thyroxine (thyroid)	1	1%	0	0%	1	2%	0.8	0.357

Note: participants could report use of more than one medication.

2. *Regional CMR_{GLC} for younger and older adults*

Supplementary Table 3. Mean and standard deviation (SD) of CMR_{GLC} (mg/100ml/min) and cortical thickness (mm) in Schaefer 100 regions for younger and older adults.

Left Hemisphere								
	CMRglc				Cortial Thickness			
	Younger		Older		Younger		Older	
Schaefer 100 Region and Suncrotical Structures	Mean	SD	Mean	SD	Mean	SD	Mean	SD
Visual Central: Extra Striate Cortex 1	3.8	1.1	3.5	0.6	2.1	0.2	2.0	0.2
Visual Central: Extra Striate Cortex 2	3.7	1.1	3.4	0.7	2.1	0.1	2.1	0.1
Visual Central: Striate Cortex 1	4.2	1.3	3.7	0.8	1.7	0.1	1.7	0.1
Visual Central: Extra Striate Cortex 3	3.3	0.9	2.9	0.5	2.2	0.1	2.1	0.1
Visual Peripheral: Extra Striate Inferior 1	4.0	1.0	3.5	0.6	2.1	0.2	1.9	0.1
Visual Peripheral: Striate Cortex Calcarine 1	3.8	1.1	3.2	0.6	1.7	0.1	1.6	0.1
Visual Peripheral: Extra Striate CortexSup 1	3.7	1.0	3.1	0.6	2.0	0.1	1.9	0.1
Somatomotor A: 1	3.5	0.9	2.8	0.5	2.3	0.1	2.1	0.2
Somatomotor A: 2	3.3	0.8	2.7	0.6	2.4	0.1	2.1	0.2
Somatomotor B: Auditory 1	3.8	1.0	3.2	0.5	2.6	0.2	2.3	0.2
Somatomotor B: S2 1	4.2	1.0	3.5	0.6	2.6	0.2	2.4	0.2
Somatomotor B: S2 2	3.9	0.9	2.9	0.6	2.8	0.1	2.6	0.2
Somatomotor B: Central 1	3.6	0.9	3.0	0.5	2.3	0.2	2.2	0.2
Dorsal Attention A: Temporal Occipital 1	3.6	0.9	3.1	0.5	2.7	0.1	2.6	0.1
Dorsal Attention A: Parietal Occipital 1	3.3	0.9	2.8	0.4	2.5	0.1	2.3	0.1
Dorsal Attention A: Superior Parietal Lobule 1	3.5	1.0	2.9	0.6	2.3	0.1	2.2	0.1
Dorsal Attention B: Post Central 1	3.3	0.9	2.5	0.5	2.5	0.2	2.4	0.2
Dorsal Attention B: Post Central 2	3.1	0.9	2.4	0.5	2.2	0.1	2.0	0.1
Dorsal Attention B: Post Central 3	3.0	0.8	2.4	0.5	2.3	0.1	2.1	0.2
Dorsal Attention B: Frontal Eye Fields 1	3.6	0.9	2.7	0.5	2.7	0.1	2.5	0.2
Salience Ventral Attention A: Parietal Operculum 1	3.8	1.0	3.0	0.5	2.7	0.2	2.5	0.2
Salience Ventral Attention A: Insula 1	3.1	0.7	2.3	0.4	3.1	0.2	2.9	0.2
Salience Ventral Attention A: Insula 2	4.7	1.1	3.5	0.7	2.8	0.1	2.6	0.1
Salience Ventral Attention A: Parietal Medial 1	3.7	0.9	3.0	0.6	2.3	0.1	2.2	0.1
Salience Ventral Attention A: Frontal Medial 1	4.1	1.0	3.1	0.7	3.0	0.2	2.7	0.2
Salience Ventral Attention B: Lateral Prefrontal Cortex 1	4.4	1.1	3.2	0.7	2.5	0.1	2.3	0.2
Salience Ventral Attention B: Medial Posterior Prefrontal 1	3.5	0.9	2.6	0.6	2.7	0.1	2.5	0.1
LimbiC: B: Orbital Frontal Cortex 1	4.3	1.2	3.4	0.6	2.6	0.1	2.5	0.1
LimbiC: A: Temporal Pole 1	2.8	0.7	2.3	0.4	3.1	0.1	3.0	0.2
LimbiC: A: Temporal Pole 2	3.9	1.0	3.2	0.6	2.9	0.2	2.7	0.2
Control A: Intraparietal Sulcus 1	3.7	1.0	2.9	0.6	2.4	0.1	2.2	0.1
Control A: Lateral Prefrontal Cortex 1	4.1	1.1	3.1	0.6	2.4	0.1	2.3	0.1
Control A: Lateral Prefrontal Cortex 2	4.0	1.1	3.0	0.6	2.7	0.1	2.5	0.1
Control B: Lateral Prefrontal Cortexv 1	4.7	1.2	3.7	0.7	2.5	0.1	2.4	0.2
Control C: Precuneus 1	4.6	1.2	4.0	0.7	2.3	0.1	2.1	0.2
Control C: Precuneus 2	3.8	0.9	3.2	0.7	2.5	0.2	2.3	0.2
Control C: Cingulate Posterior 1	3.5	0.9	3.0	0.6	2.2	0.1	2.0	0.1
Default A: Dorsal Prefrontal Cortex 1	4.4	1.2	3.1	0.7	2.7	0.1	2.5	0.2
Default A: Precuneus Posterior Cingulate Cortex1	4.6	1.2	3.9	0.8	2.6	0.1	2.4	0.1
Default A: Medial Prefrontal Cortex 1	4.0	1.0	3.0	0.6	2.6	0.1	2.5	0.1
Default B: Temp 1	3.4	0.9	2.7	0.5	3.0	0.1	2.7	0.1
Default B: Temp 2	3.8	0.9	3.1	0.6	2.6	0.1	2.4	0.1
Default B: inferior parietal lobule 1	3.7	1.0	3.0	0.6	2.7	0.1	2.4	0.1
Default B: Dorsal Prefrontal Cortex 1	4.3	1.1	3.1	0.7	2.9	0.1	2.6	0.2
Default B: Lateral Prefrontal Cortex 1	4.3	1.1	3.3	0.7	2.8	0.2	2.5	0.2
Default B: Ventral Prefrontal Cortex 1	4.2	1.1	3.2	0.6	2.9	0.1	2.8	0.1
Default B: Ventral Prefrontal Cortex 2	4.5	1.2	3.5	0.7	2.6	0.1	2.4	0.1
Default C: RetroSuperior Parietal Lobuleenial 1	4.8	1.2	4.0	0.8	2.6	0.1	2.5	0.2
Default C: Parahippocampal Cortex 1	3.0	0.7	2.5	0.5	2.6	0.2	2.5	0.2
Temporal Parietal 1	3.8	0.9	3.2	0.6	2.7	0.2	2.5	0.2
Caudate	1.8	0.5	1.5	0.3				
Putamen	1.5	0.4	1.2	0.3				
Pallidum	2.2	0.6	1.8	0.4				
Thalamus	3.8	1.0	3.1	0.7				

Right Hemisphere								
	CMRglc				Cortical Thickness			
	Younger		Older		Younger		Older	
Schaefer 100 Region and Suncrotical Structures	Mean	SD	Mean	SD	Mean	SD	Mean	SD
Visual Central: Extra Striate Cortex 1	3.8	1.0	3.5	0.6	2.3	0.2	2.2	0.1
Visual Central: Extra Striate Cortex 2	3.9	1.1	3.6	0.9	2.0	0.1	1.9	0.1
Visual Central: Extra Striate Cortex 3	3.4	0.9	3.0	0.5	2.3	0.1	2.2	0.1
Visual Peripheral: Striate Cortex Calcarine 1	4.3	1.2	3.8	0.8	1.6	0.1	1.6	0.1
Visual Peripheral: Extra Striate Inferior 1	3.5	1.0	2.9	0.5	2.0	0.1	1.8	0.1
Visual Peripheral: Extra Striate Superior 1	3.4	1.0	2.9	0.6	2.0	0.1	1.9	0.1
Somatomotor A: 1	4.1	1.0	3.3	0.7	2.3	0.2	2.1	0.2
Somatomotor A: 2	3.4	0.9	2.8	0.6	2.2	0.2	2.1	0.2
Somatomotor A: 3	2.9	0.7	2.4	0.6	2.1	0.2	1.9	0.2
Somatomotor A: 4	3.3	0.8	2.7	0.6	2.4	0.1	2.1	0.2
Somatomotor B: Auditory 1	3.6	0.9	3.0	0.5	2.6	0.2	2.4	0.2
Somatomotor B: S2 1	4.0	1.0	3.2	0.7	2.7	0.2	2.5	0.2
Somatomotor B: S2 2	3.8	1.0	2.9	0.7	2.8	0.2	2.6	0.2
Somatomotor B: Central 1	3.4	0.9	2.9	0.6	2.2	0.1	2.1	0.1
Dorsal Attention A: Temporal Occipital 1	3.4	0.9	3.0	0.5	2.6	0.1	2.5	0.1
Dorsal Attention A: Parietal Occipital 1	3.5	0.9	3.0	0.6	2.5	0.1	2.4	0.1
Dorsal Attention A: Superior Parietal Lobule 1	3.1	0.8	2.5	0.5	2.2	0.1	2.1	0.2
Dorsal Attention B: Post Central 1	3.3	1.0	2.7	0.6	2.3	0.1	2.1	0.1
Dorsal Attention B: Post Central 2	3.1	0.8	2.6	0.6	2.4	0.1	2.2	0.2
Dorsal Attention B: Frontal Eye Fields 1	3.5	0.9	2.6	0.5	2.6	0.2	2.4	0.2
Salience Ventral Attention A: Parietal Operculum 1	3.7	1.0	2.8	0.6	2.6	0.1	2.5	0.2
Salience Ventral Attention A: InsulA: 1	4.0	0.9	3.1	0.6	3.0	0.1	2.8	0.1
Salience Ventral Attention A: Parietal Medial 1	3.9	0.9	3.2	0.7	2.4	0.1	2.2	0.2
Salience Ventral Attention A: Frontal Medial 1	4.0	1.0	3.0	0.7	2.9	0.1	2.6	0.1
Salience Ventral Attention B: Inferior Parietal Lobule 1	3.5	1.0	2.7	0.6	2.7	0.2	2.5	0.2
Salience Ventral Attention B: Lateral Prefrontal Cortex 1	4.4	1.1	3.3	0.7	2.4	0.1	2.3	0.1
Salience Ventral Attention B: Medial Posterior Prefrontal 1	3.7	0.9	2.6	0.7	2.6	0.1	2.4	0.1
Limbic B: Orbital Frontal Cortex 1	4.5	1.2	3.6	0.7	2.6	0.1	2.6	0.2
Limbic A: Temporal Pole 1	2.9	0.7	2.4	0.4	3.0	0.1	2.9	0.2
Control A: Intraparietal Sulcus 1	3.1	0.9	2.5	0.6	2.3	0.1	2.1	0.1
Control A: Lateral Prefrontal Cortex 1	4.2	1.2	3.2	0.6	2.3	0.1	2.2	0.1
Control A: Lateral Prefrontal Cortex 2	3.8	1.0	2.9	0.6	2.6	0.2	2.4	0.1
Control B: Temporal 1	3.9	1.0	3.1	0.6	2.9	0.1	2.7	0.2
Control B: inferior parietal lobule 1	4.0	1.0	3.2	0.8	2.5	0.2	2.3	0.2
Control B: Lateral Prefrontal Cortexd 1	4.5	1.2	3.4	0.8	2.6	0.2	2.4	0.1
Control B: Lateral Prefrontal Cortexv 1	4.5	1.2	3.6	0.7	2.5	0.1	2.4	0.1
Control C Cingulate Posterior 1	3.9	1.0	3.2	0.7	2.2	0.1	2.0	0.2
Control C Precuneus 1	4.4	1.2	3.8	0.8	2.3	0.1	2.2	0.2
Default A: Inferior Parietal Lobule 1	3.5	0.9	2.9	0.7	2.6	0.2	2.4	0.1
Default A: Dorsal Prefrontal Cortex 1	4.1	1.1	2.9	0.6	2.7	0.1	2.4	0.2
Default A: Precuneus Posterior Cingulate Cortex 1	4.8	1.2	4.2	0.8	2.6	0.1	2.5	0.1
Default A: Medial Prefrontal Cortex 1	4.1	1.0	3.1	0.6	2.6	0.1	2.5	0.1
Default B: Dorsal Prefrontal Cortex 1	4.3	1.1	3.1	0.7	2.9	0.1	2.6	0.2
Default B: Ventral Prefrontal Cortex 1	4.2	1.1	3.3	0.6	2.8	0.2	2.6	0.2
Default B: Ventral Prefrontal Cortex 2	4.6	1.2	3.4	0.8	2.7	0.2	2.4	0.2
Default C retroSuperior Parietal Lobuleenial 1	4.7	1.2	4.0	0.8	2.7	0.1	2.5	0.2
Default C Parahippocampal Cortex 1	3.1	0.7	2.7	0.5	2.7	0.1	2.6	0.2
Temporal Parietal 1	2.8	0.6	2.3	0.4	2.9	0.2	2.7	0.2
Temporal Parietal 2	3.7	0.9	2.9	0.5	2.7	0.1	2.5	0.1
Temporal Parietal 3	3.4	0.9	2.8	0.6	2.7	0.1	2.5	0.1
Caudate	2.2	0.6	1.9	0.4				
Putamen	1.3	0.4	1.1	0.2				
Pallidum	2.1	0.5	1.7	0.4				
Thalamus	3.7	1.0	3.1	0.7				

3. Normality and Outlier Considerations

A.

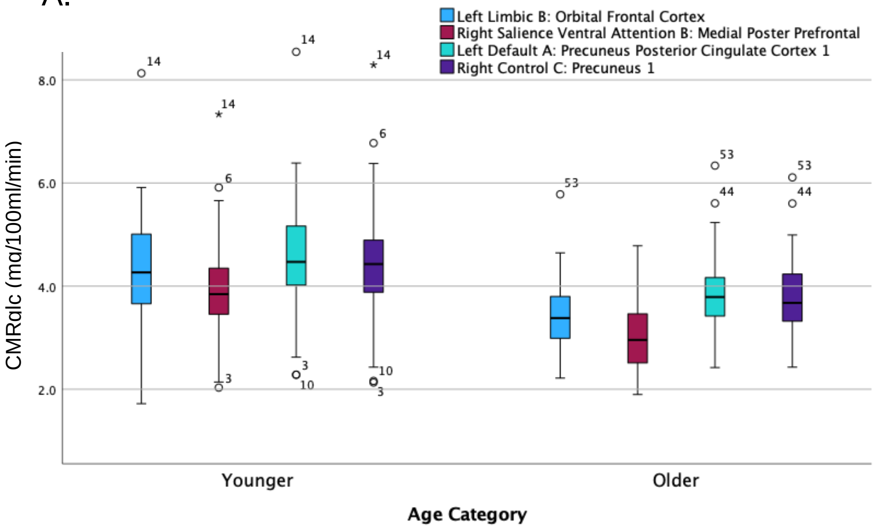

B.

Partial Correlation: Age Group and CRMglc, Controlling for Cortical Thickness						
	All Participants		2 Outliers Excluded		Log Transformed	
	r	p	r	p	r	p
Left LimbiC: B: Orbital Frontal Cortex 1	-0.43	0.001	-0.46	<.001	-0.40	<.001
Right Salience Ventral Attention B: Medial Posterior Prefrontal 1	-0.44	0.001	-0.47	<.001	-0.45	<.001
Partial Correlation: HOMA-IR and CRMglc, Controlling for Age Group						
	All Participants		2 Outliers Excluded		Log Transformed	
	r	p	r	p	r	p
Left Default A: Precuneus Posterior Cingulate Cortex 1	-0.34	0.032	-0.36	0.011	-0.37	<.001
Right Control C: Precuneus 1	-0.36	0.050	-0.38	0.011	-0.38	<.001

Supplementary Figure 1. A. Boxplot of younger and older adult for regions shown in the Figures in main manuscript and Supplementary Table 3. We found the data to have slight non-normality based on the Kolmogorov-Smirnov Test. Cases were also identified where regional CMR_{GLC} values were high or low for some participants relative to the sample distribution, although they are within a physiological plausible range (A). Analyses were run log transforming and excluding outliers in those regions and compared to the results for all participants without transformation (B). Results are similar in all cases, suggesting that the analyses are robust to the outliers and any non-normality. Hence, all participants and untransformed CRM_{GLC} data was used for the analyses presented in the manuscript.

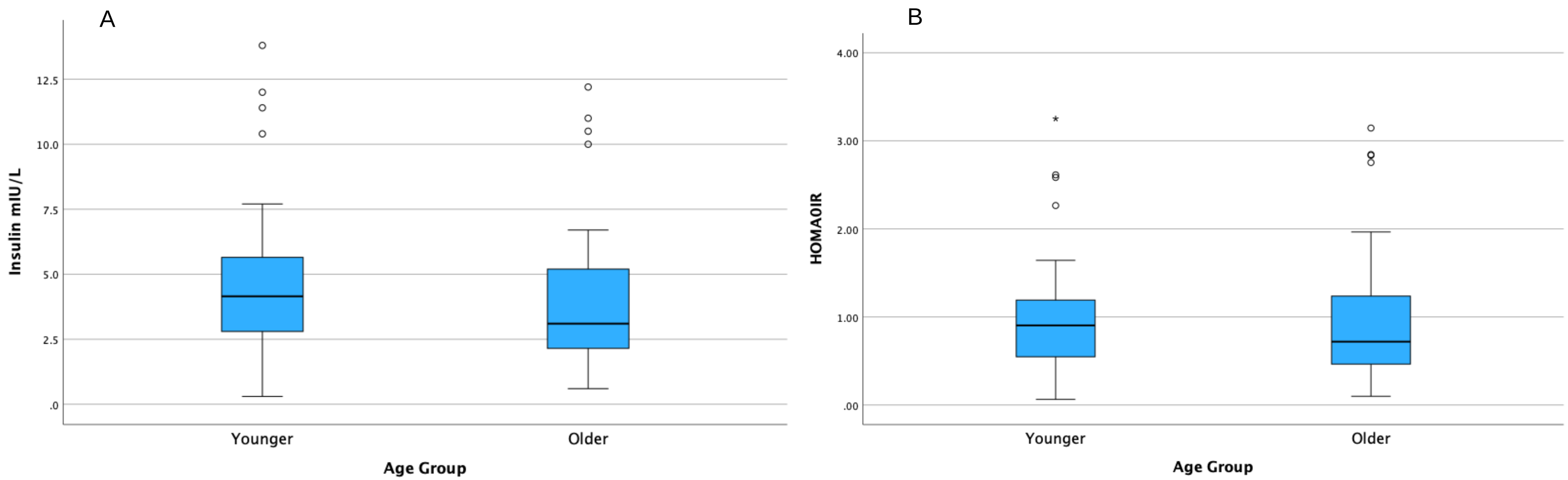

Supplementary Figure 2. Boxplot of younger and older adult: (A) insulin, and (B) HOMA-IR

4. Cortical Thickness, Age, HOMA-IR and CMR_{GLC}

Supplementary Tables 4 to 7 present the results from the main analyses in the manuscript.

Supplementary Table 4. General linear models of regional CMR_{GLC} and cortical thickness in the whole sample. The η^2_p values are plotted on the brain surface in Figure 1 of the main manuscript.

Left Hemisphere				Right Hemisphere			
	F	p-FDR	η^2_p		F	p-FDR	η^2_p
Visual Central: Extra Striate Cortex 1	2.5	0.133	0.029	Visual Central: Extra Striate Cortex 1	3.2	0.088	0.038
Visual Central: Extra Striate Cortex 2	0.5	0.515	0.006	Visual Central: Extra Striate Cortex 2	0.1	0.789	0.001
Visual Central: Striate Cortex 1	0.5	0.500	0.006	Visual Central: Extra Striate Cortex 3	4.1	0.054	0.048
Visual Central: Extra Striate Cortex 3	4.7	0.039	0.055	Visual Peripheral: Striate Cortex Calcarine 1	0.4	0.540	0.005
Visual Peripheral: Extra Striate Inferior 1	3.3	0.083	0.039	Visual Peripheral: Extra Striate Inferior 1	11.7	0.002	0.126
Visual Peripheral: Striate Cortex Calcarine 1	10.8	0.003	0.117	Visual Peripheral: Extra Striate Superior 1	6.2	0.019	0.071
Visual Peripheral: Extra Striate Cortex Sup 1	5.6	0.026	0.065				
Somatomotor A: 1	15.9	0.000	0.164	Somatomotor A: 1	7.0	0.013	0.080
Somatomotor A: 2	15.8	0.000	0.163	Somatomotor A: 2	7.2	0.013	0.081
Somatomotor B: Auditory 1	15.4	0.001	0.160	Somatomotor A: 3	14.8	0.001	0.154
Somatomotor B: S2 1	6.8	0.015	0.077	Somatomotor A: 4	13.7	0.001	0.145
Somatomotor B: S2 2	19.0	0.000	0.190	Somatomotor B: Auditory 1	18.6	0.000	0.187
Somatomotor B: Central 1	7.5	0.011	0.084	Somatomotor B: S2 1	6.6	0.016	0.075
				Somatomotor B: S2 2	14.8	0.001	0.154
				Somatomotor B: Central 1	8.3	0.008	0.093
Dorsal Attention A: Temporal Occipital 1	14.9	0.001	0.156	Dorsal Attention A: Temporal Occipital 1	2.4	0.138	0.029
Dorsal Attention A: Parietal Occipital 1	7.1	0.013	0.081	Dorsal Attention A: Parietal Occipital 1	8.6	0.007	0.096
Dorsal Attention A: Superior Parietal Lobule 1	14.6	0.001	0.153	Dorsal Attention A: Superior Parietal Lobule 1	9.6	0.005	0.106
Dorsal Attention B: Post Central 1	4.6	0.042	0.054	Dorsal Attention B: Post Central 1	15.2	0.001	0.158
Dorsal Attention B: Post Central 2	33.4	0.000	0.292	Dorsal Attention B: Post Central 2	24.2	0.000	0.230
Dorsal Attention B: Post Central 3	16.4	0.000	0.168	Dorsal Attention B: Frontal Eye Fields 1	39.1	0.000	0.325
Dorsal Attention B: Frontal Eye Fields 1	19.8	0.000	0.196				
Salience Ventral Attention A: Parietal Operculum 1	8.8	0.006	0.098	Salience Ventral Attention A: Parietal Operculum 1	10.2	0.003	0.112
Salience Ventral Attention A: Insula: 1	11.7	0.002	0.126	Salience Ventral Attention A: Insula: 1	10.3	0.003	0.113
Salience Ventral Attention A: Insula: 2	26.7	0.000	0.248	Salience Ventral Attention A: Parietal Medial 1	28.6	0.000	0.261
Salience Ventral Attention A: Parietal Medial 1	25.6	0.000	0.240	Salience Ventral Attention A: Frontal Medial 1	29.3	0.000	0.265
Salience Ventral Attention A: Frontal Medial 1	26.9	0.000	0.250	Salience Ventral Attention B: Inferior Parietal Lobule 1	10.2	0.003	0.112
Salience Ventral Attention B: Lateral Prefrontal Cortex 1	32.1	0.000	0.284	Salience Ventral Attention B: Lateral Prefrontal Cortex 1	19.7	0.000	0.196
Salience Ventral Attention B: Medial Posterior Prefrontal 1	16.6	0.000	0.170	Salience Ventral Attention B: Medial Posterior Prefrontal 1	16.7	0.000	0.171
Limbic A: Temporal Pole 1	5.5	0.026	0.064	Limbic A: Temporal Pole 1	7.0	0.013	0.080
Limbic A: Temporal Pole 2	1.4	0.250	0.017	Limbic B: Orbital Frontal Cortex 1	0.0	0.900	0.000
Limbic B: Orbital Frontal Cortex 1	0.7	0.427	0.009				
Control A: Intraparietal Sulcus 1	20.4	0.000	0.201	Control A: Intraparietal Sulcus 1	15.5	0.001	0.161
Control A: Lateral Prefrontal Cortex 1	18.4	0.000	0.185	Control A: Lateral Prefrontal Cortex 1	6.3	0.019	0.072
Control A: Lateral Prefrontal Cortex 2	30.4	0.000	0.273	Control A: Lateral Prefrontal Cortex 2	13.9	0.001	0.147
Control B: Lateral Prefrontal Cortex 1	14.3	0.001	0.150	Control B: Temporal 1	2.4	0.137	0.028
Control C: Precuneus 1	5.2	0.031	0.060	Control B: inferior parietal lobule 1	15.1	0.001	0.157
Control C: Precuneus 2	9.1	0.005	0.101	Control B: Lateral Prefrontal Cortexd 1	24.8	0.000	0.234
Control C: Cingulate Posterior 1	6.2	0.019	0.072	Control B: Lateral Prefrontal Cortexv 1	13.1	0.001	0.139
				Control C: Cingulate Posterior 1	7.9	0.009	0.089
				Control C: Precuneus 1	5.6	0.026	0.064
Default A: Dorsal Prefrontal Cortex 1	33.3	0.000	0.291	Default A: Inferior Parietal Lobule 1	21.3	0.000	0.208
Default A: Precuneus Posterior Cingulate Cortex1	8.4	0.007	0.094	Default A: Dorsal Prefrontal Cortex 1	23.4	0.000	0.224
Default A: Medial Prefrontal Cortex 1	11.6	0.002	0.125	Default A: Precuneus Posterior Cingulate Cortex 1	4.1	0.053	0.049
Default B: Temp 1	13.0	0.001	0.138	Default A: Medial Prefrontal Cortex 1	13.3	0.001	0.141
Default B: Temp 2	17.2	0.000	0.175	Default B: Dorsal Prefrontal Cortex 1	30.2	0.000	0.272
Default B: Inferior Parietal Lobule 1	14.6	0.001	0.153	Default B: Ventral Prefrontal Cortex 1	9.5	0.005	0.105
Default B: Dorsal Prefrontal Cortex 1	31.1	0.000	0.277	Default B: Ventral Prefrontal Cortex 2	16.1	0.000	0.166
Default B: Lateral Prefrontal Cortex 1	20.6	0.000	0.202	Default C: Retro Superior Parietal Lobuleenial 1	7.5	0.011	0.085
Default B: Ventral Prefrontal Cortex 1	2.8	0.106	0.034	Default C: Parahippocampal Cortex 1	8.7	0.007	0.097
Default B: Ventral Prefrontal Cortex 2	14.8	0.001	0.154				
Default C: Retro Superior Parietal Lobuleenial 1	2.9	0.102	0.035				
Default C: Parahippocampal Cortex 1	9.4	0.005	0.104				
Temporal Parietal 1	12.3	0.002	0.132	Temporal Parietal 1	15.5	0.001	0.160
				Temporal Parietal 2	16.5	0.000	0.169
				Temporal Parietal 3	10.6	0.003	0.116

Supplementary Table 5. General linear models of age group, HOMA-IR and age group x HOMA-IR effect on regional CMR_{GLC}, including cortical thickness as a covariate. The η^2_p values are plotted on the brain surface in Figure 2 of the main manuscript.

Left Hemisphere																Right Hemisphere															
	Overall Model			Cortical Thickness			Age Group			HOMA-IR			Age Group x HOMA-IR				Overall Model			Cortical Thickness			Age Group			HOMA-IR			Age Group x HOMA-IR		
	F	p-FDR	η^2_p	F	p	η^2_p	F	p	η^2_p	F	p	η^2_p	F	p	η^2_p		F	p-FDR	η^2_p	F	p	η^2_p	F	p	η^2_p	F	p	η^2_p	F	p	η^2_p
Visual Central: Extra Striate Cortex 1	2.5	0.053	0.117	2.8	0.101	0.036	4.6	0.036	0.058	4.1	0.048	0.052	1.1	0.298	0.015	Visual Central: Extra Striate Cortex 1	4.1	0.005	0.183	4.6	0.035	0.059	5.0	0.028	0.064	8.6	0.005	0.104	3.5	0.064	0.045
Visual Central: Extra Striate Cortex 2	3.1	0.022	0.142	4.3	0.042	0.055	6.0	0.017	0.074	7.5	0.008	0.092	0.2	0.635	0.003	Visual Central: Extra Striate Cortex 2	2.9	0.030	0.134	2.8	0.096	0.037	4.6	0.035	0.058	8.3	0.005	0.101	0.0	0.896	0.000
Visual Central: Striate Cortex 1	3.0	0.023	0.141	3.0	0.089	0.039	6.8	0.011	0.084	5.9	0.018	0.073	0.3	0.603	0.004	Visual Central: Extra Striate Cortex 3	5.0	0.001	0.214	6.8	0.011	0.084	8.3	0.005	0.100	9.6	0.003	0.115	1.0	0.310	0.014
Visual Central: Extra Striate Cortex 3	4.9	0.001	0.211	7.0	0.010	0.086	10.8	0.002	0.127	6.4	0.014	0.079	1.2	0.274	0.016	Visual Peripheral: Striate Cortex Calcarine 1	3.1	0.020	0.145	3.6	0.061	0.047	8.1	0.006	0.099	5.2	0.025	0.066	0.4	0.540	0.005
Visual Peripheral: Extra Striate Inferior 1	4.5	0.003	0.196	3.8	0.055	0.049	10.0	0.002	0.119	4.8	0.032	0.060	0.5	0.491	0.006	Visual Peripheral: Extra Striate Inferior 1	7.0	0.000	0.274	4.9	0.029	0.062	9.1	0.004	0.109	8.3	0.005	0.100	4.1	0.045	0.053
Visual Peripheral: Striate Cortex Calcarine 1	7.3	0.000	0.284	6.5	0.013	0.080	9.9	0.002	0.118	8.9	0.004	0.107	9.4	0.003	0.113	Visual Peripheral: Extra Striate Superior 1	6.2	0.000	0.251	7.6	0.007	0.093	11.6	0.001	0.135	9.1	0.004	0.109	2.9	0.092	0.038
Visual Peripheral: Extra Striate CortexSup 1	7.2	0.000	0.281	7.2	0.009	0.088	15.3	0.000	0.172	8.9	0.004	0.108	2.0	0.162	0.026																
Somatomotor A: 1	10.0	0.000	0.351	5.0	0.029	0.063	13.9	0.000	0.158	7.8	0.007	0.095	7.3	0.009	0.090	Somatomotor A: 1	8.2	0.000	0.306	3.3	0.071	0.043	13.4	0.000	0.153	10.2	0.002	0.121	1.9	0.171	0.025
Somatomotor A: 2	8.7	0.000	0.319	3.9	0.051	0.050	8.1	0.006	0.099	9.3	0.003	0.112	5.1	0.027	0.065	Somatomotor A: 2	8.1	0.000	0.305	5.9	0.017	0.074	15.0	0.000	0.169	9.8	0.002	0.117	5.9	0.018	0.073
Somatomotor B: Auditory 1	9.6	0.000	0.342	7.1	0.010	0.087	11.7	0.001	0.136	11.7	0.001	0.136	5.4	0.022	0.069	Somatomotor A: 3	8.5	0.000	0.315	3.7	0.058	0.048	9.9	0.002	0.118	9.0	0.004	0.109	7.0	0.010	0.086
Somatomotor B: S2 1	7.2	0.000	0.280	4.8	0.032	0.061	13.7	0.000	0.157	8.4	0.005	0.102	1.4	0.244	0.018	Somatomotor A: 4	8.8	0.000	0.322	3.8	0.055	0.049	9.3	0.003	0.111	10.6	0.002	0.125	3.8	0.056	0.049
Somatomotor B: S2 2	13.6	0.000	0.424	7.4	0.008	0.090	23.4	0.000	0.240	10.0	0.002	0.119	3.5	0.064	0.046	Somatomotor B: Auditory 1	11.8	0.000	0.389	10.0	0.002	0.119	11.5	0.001	0.135	14.1	0.000	0.160	9.9	0.002	0.118
Somatomotor B: Central 1	6.8	0.000	0.269	5.2	0.026	0.065	12.6	0.001	0.145	8.1	0.006	0.099	2.7	0.107	0.035	Somatomotor B: S2 1	7.8	0.000	0.297	3.5	0.064	0.045	14.8	0.000	0.166	8.8	0.004	0.106	0.8	0.381	0.010
																Somatomotor B: S2 2	12.0	0.000	0.393	6.9	0.011	0.085	20.5	0.000	0.217	12.2	0.001	0.141	3.3	0.071	0.043
																Somatomotor B: Central 1	6.0	0.000	0.246	4.5	0.037	0.058	8.1	0.006	0.099	8.7	0.004	0.105	2.7	0.103	0.036
Dorsal Attention A: Temporal Occipital 1	9.7	0.000	0.344	9.9	0.002	0.118	14.4	0.000	0.163	6.1	0.016	0.076	13.5	0.000	0.154	Dorsal Attention A: Temporal Occipital 1	5.2	0.001	0.220	6.8	0.011	0.084	12.8	0.001	0.147	8.1	0.006	0.099	0.5	0.481	0.007
Dorsal Attention A: Parietal Occipital 1	5.9	0.000	0.241	4.9	0.029	0.063	10.0	0.002	0.119	7.8	0.007	0.096	0.9	0.343	0.012	Dorsal Attention A: Parietal Occipital 1	7.4	0.000	0.284	8.5	0.005	0.103	13.8	0.000	0.157	8.9	0.004	0.107	2.3	0.130	0.031
Dorsal Attention A: Superior Parietal Lobule 1	8.0	0.000	0.303	5.7	0.020	0.071	10.7	0.002	0.126	7.6	0.007	0.093	4.6	0.035	0.059	Dorsal Attention A: Superior Parietal Lobule 1	9.2	0.000	0.331	6.8	0.011	0.085	14.3	0.000	0.162	12.5	0.001	0.144	4.6	0.035	0.059
Dorsal Attention B: Post Central 1	7.8	0.000	0.296	3.1	0.081	0.041	15.2	0.000	0.170	7.6	0.007	0.093	0.1	0.710	0.002	Dorsal Attention B: Post Central 1	7.9	0.000	0.300	3.4	0.070	0.044	7.1	0.010	0.087	9.1	0.003	0.110	2.3	0.134	0.030
Dorsal Attention B: Post Central 2	12.6	0.000	0.406	3.3	0.075	0.042	6.2	0.015	0.078	8.3	0.005	0.101	16.0	0.000	0.178	Dorsal Attention B: Post Central 2	11.2	0.000	0.377	7.1	0.010	0.087	9.7	0.003	0.115	9.4	0.003	0.113	15.8	0.000	0.176
Dorsal Attention B: Post Central 3	11.6	0.000	0.386	8.1	0.006	0.099	16.1	0.000	0.179	8.7	0.004	0.105	12.0	0.001	0.140	Dorsal Attention B: Frontal Eye Fields 1	15.3	0.000	0.453	2.3	0.137	0.030	9.6	0.003	0.115	7.3	0.009	0.090	13.2	0.001	0.152
Dorsal Attention B: Frontal Eye Fields 1	11.5	0.000	0.383	4.3	0.041	0.055	16.2	0.000	0.179	7.5	0.008	0.092	3.2	0.076	0.042																
Salience Ventral Attention A: Parietal Operculum 1	10.1	0.000	0.353	5.3	0.024	0.067	17.6	0.000	0.192	10.7	0.002	0.126	1.3	0.256	0.017	Salience Ventral Attention A: Parietal Operculum 1	11.6	0.000	0.386	6.0	0.016	0.076	22.8	0.000	0.236	12.3	0.001	0.142	1.6	0.211	0.021
Salience Ventral Attention A: Insula: 1	13.0	0.000	0.413	5.4	0.023	0.068	23.3	0.000	0.239	9.6	0.003	0.114	1.2	0.274	0.016	Salience Ventral Attention A: Insula: 1	10.1	0.000	0.353	4.6	0.035	0.059	18.4	0.000	0.200	9.9	0.002	0.118	0.2	0.637	0.003
Salience Ventral Attention A: Insula: 2	12.3	0.000	0.400	4.2	0.043	0.054	13.4	0.000	0.153	7.3	0.008	0.090	5.3	0.024	0.067	Salience Ventral Attention A: Parietal Medial 1	11.7	0.000	0.388	2.9	0.094	0.038	6.1	0.016	0.076	10.2	0.002	0.121	13.8	0.000	0.157
Salience Ventral Attention A: Parietal Medial 1	12.8	0.000	0.409	4.6	0.035	0.059	7.7	0.007	0.094	14.2	0.000	0.161	13.2	0.001	0.152	Salience Ventral Attention A: Frontal Medial 1	13.0	0.000	0.413	5.3	0.024	0.067	9.6	0.003	0.115	9.4	0.003	0.113	9.9	0.002	0.118
Salience Ventral Attention A: Frontal Medial 1	12.0	0.000	0.393	4.8	0.031	0.061	10.0	0.002	0.119	7.6	0.007	0.093	7.6	0.007	0.093	Salience Ventral Attention B: Inferior Parietal Lobul	9.6	0.000	0.340	5.7	0.020	0.072	18.4	0.000	0.199	8.7	0.004	0.106	1.2	0.278	0.016
Salience Ventral Attention B: Lateral Prefrontal Corte	16.6	0.000	0.473	4.6	0.036	0.058	14.7	0.000	0.166	11.7	0.001	0.136	14.3	0.000	0.162	Salience Ventral Attention B: Lateral Prefrontal Cor	12.1	0.000	0.396	5.4	0.023	0.068	18.7	0.000	0.201	7.6	0.007	0.093	5.5	0.022	0.069
Salience Ventral Attention B: Medial Posterior Prefror	11.1	0.000	0.374	2.1	0.154	0.027	12.3	0.001	0.142	9.7	0.003	0.116	1.8	0.188	0.023	Salience Ventral Attention B: Medial Posterior Prefr	12.7	0.000	0.408	1.6	0.217	0.021	15.6	0.000	0.174	8.9	0.004	0.107	0.9	0.345	0.012
Limbic A Temporal Pole 1	7.8	0.000	0.295	4.4	0.040	0.056	16.1	0.000	0.178	7.3	0.009	0.090	3.6	0.063	0.046	Limbic A: Temporal Pole 1	8.1	0.000	0.305	4.0	0.049	0.051	15.7	0.000	0.175	8.4	0.005	0.102	3.3	0.075	0.042
Limbic A: Temporal Pole 2	6.2	0.000	0.250	4.0	0.050	0.051	13.7	0.000	0.156	8.8	0.004	0.106	0.4	0.529	0.005	Limbic B: Orbital Frontal Cortex 1	7.7	0.000	0.293	3.2	0.079	0.041	16.9	0.000	0.186	8.7	0.004	0.105	0.1	0.794	0.001
Limbic B: Orbital Frontal Cortex 1	8.2	0.000	0.308	3.0	0.090	0.038	16.4	0.000	0.182	10.3	0.002	0.122	0.2	0.686	0.002																
Control A: Intraparietal Sulcus 1	10.3	0.000	0.357	5.1	0.027	0.064	12.7	0.001	0.147	7.4	0.008	0.091	5.9	0.017	0.074	Control A: Intraparietal Sulcus 1	7.0	0.000	0.275	4.9	0.030	0.062	8.5	0.005	0.103	5.4	0.023	0.068	5.7	0.020	0.071
Control A: Lateral Prefrontal Cortex 1	9.7	0.000	0.345	3.4	0.067	0.045	12.0	0.001	0.139	6.9	0.010	0.086	3.3	0.073	0.043	Control A: Lateral Prefrontal Cortex 1	9.2	0.000	0.333	4.5	0.036	0.058	20.1	0.000	0.214	9.0	0.004	0.109	0.0	0.993	0.000
Control A: Lateral Prefrontal Cortex 2	10.5	0.000	0.361	1.7	0.191	0.023	5.0	0.028	0.064	5.6	0.021	0.070	6.8	0.011	0.084	Control A: Lateral Prefrontal Cortex 2	10.1	0.000	0.353	5.2	0.025	0.066	15.9	0.000	0.177	8.9	0.004	0.108	3.1	0.082	0.040
Control B: Lateral Prefrontal Cortexv 1	10.3	0.000	0.358	4.4	0.039	0.056	16.0	0.000	0.177	8.6	0.005	0.104	6.0	0.017	0.075	Control B: Temporal 1	7.9	0.000	0.298	4.0	0.048	0.052	17.2	0.000	0.188	10.7	0.002	0.127	0.1	0.751	0.001
Control C: Precuneus 1	6.3	0.000	0.253	2.8	0.101	0.036	7.5	0.008	0.092	12.3	0.001	0.143	0.9	0.357	0.011	Control B: inferior parietal lobule 1	10.9	0.000	0.370	5.2	0.026	0.065	14.6	0.000	0.165	11.3	0.001	0.132	6.2	0.015	0.078
Control C: Precuneus 2	8.1	0.000	0.305	5.4	0.023	0.067	11.4	0.001	0.133	12.9	0.001	0.148	3.7	0.059	0.047	Control B: Lateral Prefrontal Cortexd 1	12.9	0.000	0.411	5.5	0.022	0.069	13.3	0.000	0.153	10.3	0.002	0.122	9.0	0.004	0.109
Control C: Cingulate Posterior 1	6.2	0.000	0.250	4.2	0.044	0.054	8.9	0.004	0.107	9.5	0.003	0.114	1.9	0.167	0.026	Control B: Lateral Prefrontal Cortexv 1	11.2	0.000	0.378	6.3	0.014	0.079	19.3	0.000	0.207	11.3	0.001	0.133	5.1	0.026	0.065
																Control C: Cingulate Posterior 1	7.8	0.000	0.296	3.5	0.064	0.046	11.2	0.001	0.132	11.4	0.001	0.134	1.8	0.181	0.024
																Control C: Precuneus 1	7.3	0.000	0.284	6.2	0.015	0.078	12.5	0.001	0.144	12.9	0.001	0.149	2.1	0.152	0.027
Default A: Dorsal Prefrontal Cortex 1	16.5	0.000	0.471	3.2	0.077	0.042	15.4	0.000	0.173	8.5	0.005	0.103	10.6	0.002	0.125	Default A: Inferior Parietal Lobule 1	10.1	0.000	0.352	5.8	0.019	0.072	7.0	0.010	0.087	10.7	0.002	0.127	9.0	0.004	0.108
Default A: Precuneus Posterior Cingulate Cortex1	6.7	0.000	0.266	3.8	0.054	0.049	8.3	0.005	0.101	11.8	0.001	0.138	2.8	0.100	0.036	Default A: Dorsal Prefrontal Cortex 1	13.6	0.000	0.424	4.9	0.030	0.062	19.3	0.000	0.207	6.1	0.016	0.076	6.7	0.012	0.082
Default A: Medial Prefrontal Cortex 1	12.5	0.000	0.403	3.3	0.074	0.042	21.0	0.000	0.221	8.9	0.004	0.107	1.3	0.264	0.017	Default A: Precuneus Posterior Cingulate Cortex 1	6.3	0.000	0.255	5.4	0.023	0.068	12.5	0.001	0.144	11.6	0.001	0.136	0.6	0.432	0.008
Default B: Temp 1	9.5	0.000	0.338	5.8	0.018	0.073	15.9	0.000	0.176	8.2	0.005	0.100	3.1	0.083	0.040	Default A: Medial Prefrontal Cortex 1	13.2	0.000	0.417	3.2	0.079	0.041	21.3	0.000	0.223	10.5	0.002	0.125	1.1	0.304	0.014
Default B: Temp 2	9.5	0.000	0.340	4.9	0.029	0.063	10.4	0.002	0.123	7.2	0.009	0.089	7.8	0.007	0.096	Default B: Dorsal Prefrontal Cortex 1	15.8	0.000	0.460	4.7	0.033	0.060	17.2	0.000	0.189	10.1	0.002	0.120	7.0	0.010	0.087
Default B: Inferior Parietal Lobule 1	9.2	0.000	0.333	6.1	0.016	0.076	8.8	0.004	0.107	11.4	0.001	0.133	4.8	0.031	0.061	Default B: Ventral Prefrontal Cortex 1	10.5	0.000	0.363	3.9	0.053	0.049	17.3	0.000	0.189	11.9	0.001	0.138	1.5	0.219	0.020
Default B: Dorsal Prefrontal Cortex 1	15.3	0.000	0.452	3.6	0.060	0.047	15.3	0.000	0.171	8.8	0.004	0.106	3.9	0.051	0.050	Default B: Ventral Prefrontal Cortex 2	11.1	0.000	0.376	3.5	0.066	0.045	16.4	0.000	0.181	9.6	0.003	0.115	1.8	0.178	0.024
Default B: Lateral Prefrontal Cortex 1	10.8	0.000	0.369	3.8	0.055	0.049	12.9	0.001	0.149	7.1	0.009	0.088	5.9	0.017	0.074	Default C: RetroSuperior Parietal Lobuleenial 1	6.6	0.000	0.262	4.4	0.039	0.056	9.8	0.003	0.116	9.6	0.003	0.115	4.1	0.047	0.052
Default B: Ventral Prefrontal Cortex 1	11.5	0.000	0.383	2.8	0.100	0.036	20.9	0.000	0.220	10.8	0.002	0.128	0.0	0.971	0.000	Default C: Parahippocampal Cortex 1	7.4	0.000	0.287	7.5	0.008	0.092	14.0	0.000	0.159	9.2	0.003	0.110	5.2	0.025	0.066
Default B: Ventral Prefrontal Cortex 2	10.3	0.000	0.357	4.8	0.031	0.061	15.1	0.000	0.170	10.4	0.002	0.123	2.5	0.119	0.033																
Default C: RetroSuperior Parietal Lobuleenial 1	7.4	0.000	0.286	2.7	0.108	0.035	11.1	0.001	0.130	13.5	0.000	0.155	0.7	0.422	0.009																
Default C: Parahippocampal Cortex 1	7.4	0.000	0.285	3.3	0.074	0.043	12.9	0.001	0.149	6.7	0.012	0.083	2.1	0.150	0.028																
Temporal Parietal 1	10.0	0.000	0.350	7.7	0.007	0.095	15.5	0.000	0.174	11.5	0.001	0.134	5.2	0.026	0.066	Temporal Parietal 1	11.4	0.000	0.381	7.5	0.008	0.092	18.6	0.000	0.201	8.0	0.006	0.097	6.0	0.016	0.075
																Temporal Parietal 2	11.4	0.000	0.382	7.7	0.007	0.094	13.6	0.000	0.155	13.5	0.000	0.155	5.6	0.020	0.071
																Temporal Parietal 3	7.2	0.000	0.281	5.9	0.018	0.074	11.3	0.001	0.132	7.9	0.006	0.096	3.1	0.083	0.040
Caudate	5.9	0.000	0.241	3.3	0.074	0.043	6.8	0.011	0.085	6.5	0.013	0.081	2.8	0.098	0.037	Caudate	4.7	0.002	0.204	3.0	0.086	0.039	5.3	0.025	0.067	7.2	0.009	0.088	2.1	0.149	0.030
Putamen	5.8	0.000	0.239	1.8	0.184	0.024	7.6	0.007	0.093	4.4	0.040	0.056	1.4	0.237	0.019	Putamen	5.5	0.001	0.229	2.4	0.125	0.032	7.1	0.010	0.087	5.6	0.021	0.070	1.7	0.193	0.020
Pallidum	5.7	0.001	0.236	0.7	0.422	0.009	6.0	0.017	0.075	5.8	0.019	0.072	0.8	0.373	0.011	Pallidum	5.8	0.001	0.237	1.3	0.263	0.017	6.7	0.012	0.083	5.8	0.019	0.073	1.1	0.288	0.020
Thalamus	5.6	0.001	0.232	0.9	0.353	0.012	4.3	0.041	0.055	9.6	0.003	0.115	1.4	0.245	0.018	Thalamus	5.5	0.001	0.228	0.9	0.334	0.013	4.1	0.045	0.053	10.4	0.002	0.123	1.2	0.273	0.020

Supplementary Table 6. Post hoc analyses of significant age group x HOMA-IR interactions (separate general linear models of CMR_{GLC} for younger and older adults). The models include regional cortical thickness as a covariate. Δ% is the percentage change in CMR_{GLC} from a 10% change in HOMA-IR, calculated from the slope of the regression lines.

Left Hemisphere

	Age Group x HOMA-IR			Post-Hoc Younger			Post-Hoc Older		
	F	p	η^2_p	F	p	Δ%	F	p	Δ%
Visual Central: Extra Striate Cortex 1	1.1	0.298	0.015						
Visual Central: Extra Striate Cortex 2	0.2	0.635	0.003						
Visual Central: Striate Cortex 1	0.3	0.603	0.004						
Visual Central: Extra Striate Cortex 3	1.2	0.274	0.016						
Visual Peripheral: Extra Striate Inferior 1	0.5	0.491	0.006						
Visual Peripheral: Striate Cortex Calcarine 1	9.4	0.003	0.113	9.5	0.004	-6.1	0.1	0.799	0.0
Visual Peripheral: Extra Striate CortexSup 1	2.0	0.162	0.026						
Somatomotor A: 1	7.3	0.009	0.090	7.1	0.012	-5.1	0.3	0.574	-0.5
Somatomotor A: 2	5.1	0.027	0.065	7.9	0.008	-5.1	1.0	0.315	-1.0
Somatomotor B: Auditory 1	5.4	0.022	0.069	10.8	0.002	-6.3	0.8	0.366	-1.3
Somatomotor B: S2 1	1.4	0.244	0.018						
Somatomotor B: S2 2	3.5	0.064	0.046						
Somatomotor B: Central 1	2.7	0.107	0.035						
Dorsal Attention A: Temporal Occipital 1	13.5	0.000	0.154	8.5	0.006	-5.2	0.4	0.509	-0.5
Dorsal Attention A: Parietal Occipital 1	0.9	0.343	0.012						
Dorsal Attention A: Superior Parietal Lobule 1	4.6	0.035	0.059	7.6	0.009	-6.0	0.1	0.819	-0.9
Dorsal Attention B: Post Central 1	0.1	0.710	0.002						
Dorsal Attention B: Post Central 2	16.0	0.000	0.178	6.1	0.019	-5.1	1.0	0.315	-0.9
Dorsal Attention B: Post Central 3	12.0	0.001	0.140	8.6	0.006	-5.1	0.0	0.925	-0.6
Dorsal Attention B: Frontal Eye Fields 1	3.2	0.076	0.042						
Salience Ventral Attention A: Parietal Operculum 1	1.3	0.256	0.017						
Salience Ventral Attention A: Insula: 1	1.2	0.274	0.016						
Salience Ventral Attention A: Insula: 2	5.3	0.024	0.067	7.4	0.010	-6.4	0.6	0.425	-1.4
Salience Ventral Attention A: Parietal Medial 1	13.2	0.001	0.152	11.1	0.002	-6.0	2.1	0.155	-1.7
Salience Ventral Attention A: Frontal Medial 1	7.6	0.007	0.093	6.3	0.017	-5.8	0.0	0.915	-2.2
Salience Ventral Attention B: Lateral Prefrontal Cortex 1	14.3	0.000	0.162	7.7	0.009	-6.1	2.0	0.164	-2.0
Salience Ventral Attention B: Medial Posterior Prefrontal	1.8	0.188	0.023						
Limbic A Temporal Pole 1	3.6	0.063	0.046						
Limbic A: Temporal Pole 2	0.4	0.529	0.005						
Limbic B: Orbital Frontal Cortex 1	0.2	0.686	0.002						
Control A: Intraparietal Sulcus 1	5.9	0.017	0.074	6.6	0.015	-5.7	0.2	0.655	-0.8
Control A: Lateral Prefrontal Cortex 1	3.3	0.073	0.043						
Control A: Lateral Prefrontal Cortex 2	6.8	0.011	0.084	3.6	0.065	-5.5	1.2	0.288	-1.6
Control B: Lateral Prefrontal Cortexv 1	6.0	0.017	0.075	7.3	0.011	-7.0	0.8	0.368	-1.5
Control C: Precuneus 1	0.9	0.357	0.011						
Control C: Precuneus 2	3.7	0.059	0.047						
Control C: Cingulate Posterior 1	1.9	0.167	0.026	6.5	0.015	-5.5	0.7	0.423	-1.3
Default A: Dorsal Prefrontal Cortex 1	10.6	0.002	0.125	6.3	0.017	-6.2	1.5	0.226	-2.2
Default A: Precuneus Posterior Cingulate Cortex1	2.8	0.100	0.036						
Default A: Medial Prefrontal Cortex 1	1.3	0.264	0.017						
Default B: Temp 1	3.1	0.083	0.040						
Default B: Temp 2	7.8	0.007	0.096	6.5	0.016	-4.9	0.2	0.673	-1.0
Default B: Inferior Parietal Lobule 1	4.8	0.031	0.061	8.9	0.005	-6.1	0.7	0.424	-1.3
Default B: Dorsal Prefrontal Cortex 1	3.9	0.051	0.050						
Default B: Lateral Prefrontal Cortex 1	5.9	0.017	0.074	5.5	0.025	-5.5	0.4	0.509	-1.5
Default B: Ventral Prefrontal Cortex 1	0.0	0.971	0.000						
Default B: Ventral Prefrontal Cortex 2	2.5	0.119	0.033						
Default C: RetroSuperior Parietal Lobuleenial 1	0.7	0.422	0.009						
Default C: Parahippocampal Cortex 1	2.1	0.150	0.028						
Temporal Parietal 1	5.2	0.026	0.066	10.8	0.002	-6.1	0.3	0.585	-0.8
Caudate	2.8	0.098	0.037						
Putamen	1.4	0.237	0.019						
Pallidum	0.8	0.373	0.011						
Thalamus	1.4	0.245	0.018						

Right Hemisphere

	Age Group x HOMA-IR			Post-Hoc Younger			Post-Hoc Older		
	F	p	η^2_p	F	p	Δ%	F	p	Δ%
Visual Central: Extra Striate Cortex 1	3.5	0.064	0.045						
Visual Central: Extra Striate Cortex 2	0.0	0.896	0.000						
Visual Central: Extra Striate Cortex 3	1.0	0.310	0.014						
Visual Peripheral: Striate Cortex Calcarine 1	0.4	0.540	0.005						
Visual Peripheral: Extra Striate Inferior 1	4.1	0.045	0.053	8.6	0.006	-5.9	0.3	0.562	-1.3
Visual Peripheral: Extra Striate Superior 1	2.9	0.092	0.038						
Somatomotor A: 1	1.9	0.171	0.025						
Somatomotor A: 2	5.9	0.018	0.073	6.7	0.014	-5.2	0.5	0.485	-1.3
Somatomotor A: 3	7.0	0.010	0.086	7.5	0.010	-5.2	0.7	0.400	-1.1
Somatomotor A: 4	3.8	0.056	0.049						
Somatomotor B: Auditory 1	9.9	0.002	0.118	15.4	0.000	-5.7	0.4	0.535	-0.3
Somatomotor B: S2 1	0.8	0.381	0.010						
Somatomotor B: S2 2	3.3	0.071	0.043						
Somatomotor B: Central 1	2.7	0.103	0.036						
Dorsal Attention A: Temporal Occipital 1	0.5	0.481	0.007						
Dorsal Attention A: Parietal Occipital 1	2.3	0.130	0.031						
Dorsal Attention A: Superior Parietal Lobule 1	4.6	0.035	0.059	10.4	0.003	-5.4	0.9	0.359	-1.3
Dorsal Attention B: Post Central 1	2.3	0.134	0.030						
Dorsal Attention B: Post Central 2	15.8	0.000	0.176	9.3	0.004	-4.7	0.1	0.784	-1.7
Dorsal Attention B: Frontal Eye Fields 1	13.2	0.001	0.152	4.9	0.034	-5.2	0.9	0.341	-1.6
Salience Ventral Attention A: Parietal Operculum 1	1.6	0.211	0.021						
Salience Ventral Attention A: Insula: 1	0.2	0.637	0.003						
Salience Ventral Attention A: Parietal Medial 1	13.8	0.000	0.157	7.6	0.009	-4.9	1.9	0.177	-2.0
Salience Ventral Attention A: Frontal Medial 1	9.9	0.002	0.118	8.6	0.006	-5.6	0.3	0.562	-1.8
Salience Ventral Attention B: Inferior Parietal Lobule 1	1.2	0.278	0.016						
Salience Ventral Attention B: Lateral Prefrontal Cortex 1	5.5	0.022	0.069	7.3	0.011	-6.3	0.0	0.976	-0.9
Salience Ventral Attention B: Medial Posterior Prefrontal 1	0.9	0.345	0.012						
Limbic A: Temporal Pole 1	3.3	0.075	0.042						
Limbic B: Orbital Frontal Cortex 1									
	0.1	0.794	0.001						
Control A: Intraparietal Sulcus 1	5.7	0.020	0.071	6.4	0.017	-6.3	0.0	0.950	-1.5
Control A: Lateral Prefrontal Cortex 1	0.0	0.993	0.000						
Control A: Lateral Prefrontal Cortex 2	3.1	0.082	0.040						
Control B: Temporal 1	0.1	0.751	0.001						
Control B: inferior parietal lobule 1	6.2	0.015	0.078	11.1	0.002	-7.3	0.7	0.417	-2.0
Control B: Lateral Prefrontal Cortexd 1	9.0	0.004	0.109	7.4	0.010	-7.7	0.0	0.832	-2.0
Control B: Lateral Prefrontal Cortexv 1	5.1	0.026	0.065	11.2	0.002	-5.9	1.2	0.279	-1.6
Control C: Cingulate Posterior 1	1.8	0.181	0.024						
Control C: Precuneus 1	2.1	0.152	0.027						
Default A: Inferior Parietal Lobule 1	9.0	0.004	0.108	9.3	0.005	-5.5	0.2	0.660	-2.0
Default A: Dorsal Prefrontal Cortex 1	6.7	0.012	0.082	5.8	0.022	-7.0	0.1	0.722	-1.8
Default A: Precuneus Posterior Cingulate Cortex 1	0.6	0.432	0.008						
Default A: Medial Prefrontal Cortex 1	1.1	0.304	0.014						
Default B: Dorsal Prefrontal Cortex 1	7.0	0.010	0.087	7.0	0.012	-6.6	0.4	0.506	-0.9
Default B: Ventral Prefrontal Cortex 1	1.5	0.219	0.020						
Default B: Ventral Prefrontal Cortex 2	1.8	0.178	0.024						
Default C: RetroSuperior Parietal Lobuleenial 1	4.1	0.047	0.052	9.2	0.005	-7.0	0.7	0.393	-1.1
Default C: Parahippocampal Cortex 1	5.2	0.025	0.066	9.9	0.004	-3.9	0.1	0.796	-0.8
Temporal Parietal 1	6.0	0.016	0.075	8.5	0.006	-3.3	0.0	0.958	-0.9
Temporal Parietal 2	5.6	0.020	0.071	12.8	0.001	-4.0	0.9	0.359	-0.9
Temporal Parietal 3	3.1	0.083	0.040						
Caudate	2.1	0.149	0.028						
Putamen	1.7	0.193	0.023						
Pallidum	1.1	0.288	0.015						
Thalamus	1.2	0.273	0.016						

Supplementary Table 7. General linear models of age group, HOMA-IR and age group x HOMA-IR effects on network CMR_{GLC}, including cortical thickness as a covariate. Includes post-hoc analyses of significant interactions as GLM for younger and older adults separately.

	Overall Model			Cortical Thickness			Age Cat			HOMA-IR			Age Group x HOMA-IR			Post hoc - Younger			Post hoc Older		
	F	p-FDR	η^2_p	F	p	η^2_p	F	p	η^2_p	F	p	η^2_p	F	p	η^2_p	F	p	η^2_p	F	p	η^2_p
Visual Central	3.8	0.007	0.171	1.2	0.283	0.016	6.5	0.013	0.081	6.5	0.013	0.081	7.7	0.007	0.095	7.7	0.009	0.188	0.4	0.523	0.010
Visual Peripheral	6.3	0.000	0.254	4.0	0.048	0.052	10.2	0.002	0.121	10.2	0.002	0.121	8.4	0.005	0.101	9.0	0.005	0.215	0.1	0.749	0.003
Somatomotor A	10.0	0.000	0.352	6.8	0.011	0.084	10.5	0.002	0.124	10.5	0.002	0.124	10.8	0.002	0.128	8.8	0.006	0.210	0.9	0.351	0.022
Somatomotor B	11.4	0.000	0.381	6.3	0.014	0.079	14.5	0.000	0.163	14.5	0.000	0.163	11.6	0.001	0.136	14.2	0.001	0.300	0.5	0.488	0.012
Dors Attention A	8.6	0.000	0.318	6.0	0.017	0.075	10.6	0.002	0.125	10.6	0.002	0.125	9.1	0.004	0.109	8.8	0.006	0.210	0.0	0.858	0.001
Dors Attention B	13.8	0.000	0.427	13.0	0.001	0.149	7.8	0.007	0.095	7.8	0.007	0.095	8.1	0.006	0.099	7.1	0.012	0.177	0.3	0.606	0.007
Salience Ventral Attention A	13.0	0.000	0.412	6.5	0.013	0.080	10.3	0.002	0.122	10.3	0.002	0.122	10.5	0.002	0.124	9.7	0.004	0.227	0.5	0.490	0.012
Salience Ventral Attention B	13.8	0.000	0.427	5.8	0.018	0.073	12.0	0.001	0.139	12.0	0.001	0.139	10.6	0.002	0.125	7.5	0.010	0.185	1.4	0.249	0.033
Limbic A	8.1	0.000	0.304	3.8	0.054	0.049	16.2	0.000	0.180	16.2	0.000	0.180	8.5	0.005	0.103	6.8	0.014	0.170	1.9	0.175	0.046
Limbic B	8.0	0.000	0.301	0.1	0.707	0.002	16.7	0.000	0.184	16.7	0.000	0.184	9.5	0.003	0.114	7.2	0.011	0.179	0.3	0.586	0.007
Control A	11.0	0.000	0.372	6.4	0.014	0.079	9.1	0.003	0.110	9.1	0.003	0.110	7.3	0.008	0.090	6.8	0.013	0.172	0.1	0.708	0.004
Control B	11.5	0.000	0.384	6.8	0.011	0.084	14.5	0.000	0.164	14.5	0.000	0.164	10.5	0.002	0.124	8.6	0.006	0.207	0.3	0.576	0.008
Control C	9.9	0.000	0.349	6.6	0.012	0.082	9.8	0.002	0.117	9.8	0.002	0.117	12.5	0.001	0.145	12.2	0.001	0.270	0.9	0.362	0.021
Default A	12.4	0.000	0.401	8.5	0.005	0.103	11.4	0.001	0.133	11.4	0.001	0.133	10.3	0.002	0.122	8.9	0.005	0.212	0.4	0.522	0.010
Default B	13.7	0.000	0.425	6.6	0.012	0.082	11.8	0.001	0.137	11.8	0.001	0.137	10.8	0.002	0.128	8.0	0.008	0.195	0.5	0.481	0.012
Default C	8.1	0.000	0.303	5.1	0.027	0.064	11.4	0.001	0.133	11.4	0.001	0.133	9.7	0.003	0.116	9.0	0.005	0.214	0.5	0.464	0.013
Temporal Parietal	11.4	0.000	0.382	7.6	0.007	0.093	12.5	0.001	0.144	12.5	0.001	0.144	11.9	0.001	0.139	12.8	0.001	0.279	0.2	0.644	0.005
Subcortical	5.8	0.000	0.237	1.7	0.202	0.022	5.7	0.020	0.071	5.7	0.020	0.071	8.4	0.005	0.102	6.0	0.020	0.154	2.5	0.119	0.060

5. Principal Component and GLM Analyses for Cognitive Variables

Supplementary Tables 8 and 9 present the results from the main analyses in the manuscript using PCA to reduce the data dimensions in the cognitive measures, and GLMs predicting cognition from network CMR_{GLC}.

Supplementary Table 8. Principal component analysis (loadings) of cognitive variables identifying five components explaining 81% of the variance.

	Component				
	1	2	3	4	5
Variance Explained (%)	18.3	17.8	17.0	14.1	13.8
HVLT: Delayed recall (total)	-0.209	0.064	0.058	0.266	0.779
HVLT: Recognition discrimination index	-0.005	-0.256	-0.130	-0.029	0.748
Digit Span: Forward (longest)	0.124	0.052	0.052	0.888	-0.010
Digit Span: Backwards (longest)	-0.160	-0.144	-0.094	0.817	0.212
Category Switch: Reaction time in switch trials	0.848	0.339	0.191	-0.087	-0.039
Category Switch: SSRT	0.938	0.105	0.075	0.042	-0.084
Digit Substitution: Correct count	-0.260	-0.915	-0.165	0.057	0.047
Digit Substitution: Seconds per correct count	0.156	0.916	0.017	-0.024	-0.040
Stop Signal: Mean stop signal delays	0.118	0.045	0.944	0.038	0.034
Stop Signal: Stop signal reaction time	0.114	0.120	0.930	-0.069	-0.063
WASI FSIQ2 T-score	0.456	0.206	0.097	0.016	0.534

Supplementary Table 9. General Linear Models predicting each of the 5 principal components from network CMR_{GLC} 17 networks, age group, HOMA-IR and cortical thickness.

	Principal Component 1			Principal Component 2			Principal Component 3			Principal Component 4			Principal Component 5		
	F	p	η^2_p	F	p	η^2_p	F	p	η^2_p	F	p	η^2_p	F	p	η^2_p
Visual Central	1.6	0.206	0.031	0.9	0.358	0.016	6.3	0.015	0.108	0.5	0.498	0.009	1.3	0.256	0.025
Visual Peripheral	0.0	0.917	0.000	0.2	0.665	0.004	4.7	0.034	0.084	0.2	0.682	0.003	0.6	0.457	0.011
Somatomotor A	0.0	0.988	0.000	0.0	0.839	0.001	1.6	0.209	0.030	0.7	0.394	0.014	0.3	0.582	0.006
Somatomotor B	0.1	0.781	0.001	0.3	0.599	0.005	0.1	0.735	0.002	0.0	0.851	0.001	0.3	0.587	0.006
Dors Attention A	0.0	0.934	0.000	0.2	0.637	0.004	5.2	0.027	0.090	0.2	0.691	0.003	0.9	0.359	0.016
Dors Attention B	0.7	0.393	0.014	1.6	0.209	0.030	1.7	0.196	0.032	0.3	0.582	0.006	0.3	0.588	0.006
Salience Ventral Attention A	0.8	0.362	0.016	1.4	0.237	0.027	0.1	0.782	0.001	3.6	0.065	0.064	0.0	0.996	0.000
Salience Ventral Attention B	0.3	0.579	0.006	0.6	0.451	0.011	0.4	0.553	0.007	1.5	0.224	0.028	0.3	0.607	0.005
Limbic A	4.4	0.042	0.077	0.3	0.600	0.005	0.2	0.684	0.003	0.7	0.413	0.013	2.0	0.164	0.037
Limbic B	0.1	0.721	0.002	0.5	0.471	0.010	1.2	0.276	0.023	0.1	0.749	0.002	0.1	0.780	0.002
Control A	0.6	0.447	0.011	4.2	0.046	0.074	5.3	0.026	0.092	1.1	0.303	0.020	1.5	0.223	0.028
Control B	0.6	0.459	0.011	1.0	0.330	0.018	1.9	0.175	0.035	0.5	0.467	0.010	0.0	0.934	0.000
Control C	0.1	0.751	0.002	0.6	0.431	0.012	0.3	0.604	0.005	0.0	0.846	0.001	0.1	0.798	0.001
Default A	0.1	0.751	0.002	0.1	0.811	0.001	3.2	0.079	0.058	0.0	0.903	0.000	0.2	0.669	0.004
Default B	0.0	0.837	0.001	4.7	0.035	0.083	1.1	0.293	0.021	0.2	0.682	0.003	0.6	0.425	0.012
Default C	5.9	0.018	0.102	0.3	0.564	0.006	1.6	0.206	0.031	0.0	0.846	0.001	0.8	0.390	0.014
Temporal Parietal	1.0	0.313	0.020	0.5	0.493	0.009	0.7	0.398	0.014	2.8	0.101	0.051	0.0	0.912	0.000
Subcortical	4.7	0.035	0.083	0.9	0.358	0.016	0.6	0.427	0.012	0.1	0.776	0.002	0.1	0.774	0.002
Cortical Thickness	2.4	0.129	0.044	1.2	0.278	0.023	1.6	0.213	0.030	0.1	0.788	0.001	0.8	0.383	0.015
Age Category	9.5	0.003	0.154	5.5	0.023	0.095	0.3	0.601	0.005	1.3	0.264	0.024	0.7	0.421	0.012
HOMA-IR	0.2	0.664	0.004	0.0	0.944	0.000	0.5	0.470	0.010	4.9	0.031	0.086	0.0	0.942	0.000

6. Effects of demographic variables and HOMA-IR2 on CMR_{GLU}, Cortical Thickness and Cognition

Effect of Other Demographics on Regional CMR_{GLC}

We undertook GLMs of regional CMR_{GLC} with the demographic variables included as covariates (Supplementary Table 10). Age group, cortical thickness and HOMA-IR remained the predominant predictors of CRM_{GLC} across most regions when the other demographics were added to the GLMs, although the relationships were somewhat attenuated or mediated by the other factors (cf. $\eta^2_{p,}$ Supplementary Table 10 vs 5). We note sex and HOMA-IR were moderately correlated ($r = -.289$, $p = .010$), suggesting that sex shared variance with the HOMA-IR and CMR_{GLC} relationships in the GLMs. We do not rule out the possibility that sex or the other demographic variables (e.g., education) independently predicts regional CMR_{GLC}, something that could be tested in future papers.

HOMA-IR vs HOMA-IR2

We compared the relationship between HOMA-IR and HOMA-IR2 with CMR_{GLC} and found minimal to no differences (cf. supplementary Tables 5 and 11). This was expected as HOMA-IR2 models increases in the insulin secretion curve for plasma glucose concentrations above 10 mmol/L (1); a threshold that than none of our participants reached. Hence, the results reported here are based on HOMA-IR.

Effect of Sex, Years of Education, Medication Use and Country of Birth on Cognition

We repeated the GLMs from the main manuscript examining the effect of network CMR_{GLC} on cognition, adding years of education, sex, medication use and country of birth as covariates. Years of education, sex and medication user were not significant predictors of cognition when the other covariates were also in the model, i.e., network CMR_{GLC}, age group, HOMA-IR and cortical thickness (Supplementary Table 12). The effect of country of birth was significant for PC1 (cognitive control), with faster reaction time among non-Australian born participants. Although the reason for these differences is unclear, previous studies have reported cross-cultural differences in processing speed, particularly in favour of Chinese versus US groups (2). We also note that the age group effect on PC1 was no longer significant with the additional demographic variables in the analyses (cf. Supplementary Tables 12 and 9), suggesting that the higher proportion of non-Australian born participants in the younger group shared variance with the age group in predicting PC1 (note: $r = .36$, $p < .001$ between age group and country of birth and $r = .34$, $p < .001$ between age group and number of medications). The number of significant covariates ($p < .05$) was also reduced relative to the models in Supplementary Table 9, suggesting that years of education, sex, medication use and cultural background share common variance with the other primary study variables of age and HOMA-IR. We do not rule out the possibility that these other demographic variables independently predicts cognition, something that could be tested in future papers.

Supplementary Table 10. General linear models testing the association of regional CMR_{GLC} with cortical thickness and other demographic variables.

	Overall Model			Cortical Thickness			Age Group			HOMA-IR			Systolic BP		
	F	p-FDR	η^2_p	F	p	η^2_p	F	p	η^2_p	F	p	η^2_p	F	p	η^2_p
Visual Central: Extra Striate Cortex 1	1.0	0.515	0.163	1.6	0.21	0.03	3.3	0.075	0.055	1.5	0.223	0.026	1.5	0.229	0.026
Visual Central: Extra Striate Cortex 2	1.0	0.512	0.162	0.4	0.53	0.01	3.1	0.083	0.053	2.9	0.094	0.049	0.7	0.394	0.013
Visual Central: Striate Cortex 1	1.1	0.434	0.176	0.4	0.55	0.01	3.7	0.058	0.063	2.4	0.129	0.041	0.9	0.336	0.017
Visual Central: Extra Striate Cortex 3	1.5	0.187	0.227	2.8	0.10	0.05	6.4	0.014	0.102	3.0	0.087	0.052	1.7	0.197	0.030
Visual Peripheral: Extra Striate Inferior 1	1.5	0.186	0.227	0.8	0.37	0.01	5.8	0.020	0.094	1.8	0.181	0.032	1.8	0.190	0.030
Visual Peripheral: Striate Cortex Calcarine 1	2.0	0.064	0.278	5.9	0.02	0.10	2.9	0.093	0.050	3.5	0.067	0.059	1.3	0.256	0.023
Visual Peripheral: Extra Striate CortexSup 1	2.0	0.059	0.283	1.0	0.32	0.02	4.9	0.030	0.081	4.2	0.046	0.070	0.6	0.441	0.011
Somatomotor A: 1	3.7	0.003	0.423	11.5	0.00	0.17	8.3	0.006	0.129	5.7	0.020	0.092	3.4	0.072	0.057
Somatomotor A: 2	3.2	0.005	0.384	8.0	0.01	0.13	5.0	0.029	0.082	4.8	0.033	0.079	3.9	0.053	0.065
Somatomotor B: Auditory 1	2.8	0.010	0.359	4.6	0.04	0.08	3.5	0.065	0.060	4.3	0.043	0.071	1.3	0.266	0.022
Somatomotor B: S2 1	2.4	0.026	0.317	0.3	0.57	0.01	6.0	0.017	0.097	3.0	0.089	0.051	1.2	0.287	0.020
Somatomotor B: S2 2	3.9	0.002	0.433	2.8	0.10	0.05	6.8	0.012	0.108	2.1	0.154	0.036	0.9	0.346	0.016
Somatomotor B: Central 1	2.2	0.039	0.301	3.9	0.05	0.07	6.8	0.011	0.109	3.9	0.054	0.065	1.6	0.205	0.028
Dorsal Attention A: Temporal Occipital 1	2.8	0.011	0.355	10.7	0.00	0.16	1.6	0.216	0.027	2.5	0.121	0.042	0.0	0.831	0.001
Dorsal Attention A: Parietal Occipital 1	2.2	0.038	0.302	3.6	0.06	0.06	6.2	0.016	0.099	2.2	0.142	0.038	2.1	0.158	0.035
Dorsal Attention A: Superior Parietal Lobule 1	2.9	0.008	0.364	6.0	0.02	0.10	5.3	0.025	0.086	2.9	0.093	0.050	1.8	0.189	0.031
Dorsal Attention B: Post Central 1	2.6	0.016	0.339	0.2	0.62	0.00	10.2	0.002	0.154	2.9	0.093	0.050	0.5	0.477	0.009
Dorsal Attention B: Post Central 2	4.9	0.001	0.490	19.6	0.00	0.26	4.2	0.045	0.070	8.0	0.006	0.125	0.5	0.505	0.008
Dorsal Attention B: Post Central 3	3.2	0.005	0.387	9.3	0.00	0.14	8.5	0.005	0.131	3.8	0.058	0.063	2.7	0.108	0.045
Dorsal Attention B: Frontal Eye Fields 1	3.9	0.002	0.431	4.7	0.03	0.08	9.7	0.003	0.148	3.9	0.055	0.064	3.0	0.090	0.050
Salience Ventral Attention A: Parietal Operculum 1	3.3	0.005	0.391	2.2	0.14	0.04	8.8	0.004	0.136	3.8	0.055	0.064	1.0	0.318	0.018
Salience Ventral Attention A: Insula: 1	3.7	0.003	0.420	0.5	0.47	0.01	12.2	0.001	0.178	3.7	0.060	0.062	0.9	0.348	0.016
Salience Ventral Attention A: Insula: 2	4.3	0.001	0.460	7.0	0.01	0.11	7.0	0.010	0.112	1.2	0.284	0.020	2.9	0.094	0.049
Salience Ventral Attention A: Parietal Medial 1	4.3	0.001	0.460	15.2	0.00	0.21	1.5	0.231	0.026	4.5	0.038	0.074	1.8	0.191	0.030
Salience Ventral Attention A: Frontal Medial 1	4.0	0.002	0.439	3.9	0.05	0.07	6.1	0.017	0.098	2.6	0.110	0.045	2.5	0.120	0.043
Salience Ventral Attention B: Lateral Prefrontal Cor	5.1	0.000	0.502	10.5	0.00	0.16	10.5	0.002	0.158	5.3	0.025	0.087	3.1	0.086	0.052
Salience Ventral Attention B: Medial Posterior Prefr	3.5	0.003	0.409	0.3	0.60	0.00	7.3	0.009	0.115	3.4	0.069	0.058	1.2	0.278	0.021
Limbic A Temporal Pole 1	2.5	0.021	0.327	0.2	0.66	0.00	9.8	0.003	0.149	3.2	0.078	0.054	1.2	0.274	0.021
Limbic A: Temporal Pole 2	2.3	0.028	0.315	2.0	0.17	0.03	6.1	0.017	0.098	2.3	0.131	0.040	0.6	0.429	0.011
Limbic B: Orbital Frontal Cortex 1	2.1	0.048	0.292	0.7	0.42	0.01	8.8	0.005	0.135	3.0	0.088	0.051	1.8	0.184	0.031
Control A: Intraparietal Sulcus 1	4.0	0.002	0.439	7.0	0.01	0.11	7.4	0.009	0.116	3.5	0.065	0.060	2.1	0.151	0.037
Control A: Lateral Prefrontal Cortex 1	3.3	0.004	0.396	3.1	0.09	0.05	8.0	0.006	0.125	1.8	0.183	0.031	2.2	0.141	0.038
Control A: Lateral Prefrontal Cortex 2	3.8	0.002	0.429	9.3	0.00	0.14	3.5	0.067	0.059	0.9	0.345	0.016	2.6	0.113	0.044
Control B: Lateral Prefrontal Cortexv 1	3.3	0.005	0.392	4.7	0.03	0.08	10.0	0.002	0.152	1.8	0.183	0.031	2.6	0.112	0.045
Control C: Precuneus 1	2.7	0.012	0.350	2.1	0.15	0.04	7.7	0.007	0.121	7.3	0.009	0.116	3.3	0.073	0.056
Control C: Precuneus 2	2.9	0.008	0.366	4.8	0.03	0.08	5.5	0.023	0.089	5.7	0.020	0.092	1.4	0.246	0.024
Control C: Cingulate Posterior 1	1.8	0.092	0.261	1.2	0.28	0.02	3.9	0.053	0.065	2.7	0.103	0.047	1.4	0.246	0.024
Default A: Dorsal Prefrontal Cortex 1	6.0	0.000	0.542	11.6	0.00	0.17	15.2	0.000	0.213	2.5	0.123	0.042	3.7	0.061	0.061
Default A: Precuneus Posterior Cingulate Cortex1	2.0	0.059	0.283	1.8	0.18	0.03	3.2	0.080	0.054	4.5	0.038	0.075	1.1	0.308	0.019
Default A: Medial Prefrontal Cortex 1	3.9	0.002	0.433	0.7	0.40	0.01	12.1	0.001	0.178	2.7	0.109	0.045	1.0	0.322	0.018
Default B: Temp 1	2.7	0.014	0.346	1.1	0.30	0.02	6.3	0.015	0.101	2.5	0.123	0.042	1.0	0.333	0.017
Default B: Temp 2	3.5	0.003	0.409	11.4	0.00	0.17	4.1	0.046	0.069	2.7	0.104	0.047	1.0	0.321	0.018
Default B: Inferior Parietal Lobule 1	3.4	0.004	0.398	7.0	0.01	0.11	3.7	0.060	0.062	4.2	0.045	0.070	2.3	0.132	0.040
Default B: Dorsal Prefrontal Cortex 1	5.0	0.001	0.494	1.9	0.17	0.03	12.2	0.001	0.179	3.2	0.078	0.054	2.1	0.154	0.036
Default B: Lateral Prefrontal Cortex 1	3.5	0.003	0.407	5.0	0.03	0.08	8.5	0.005	0.132	3.1	0.082	0.053	1.8	0.180	0.032
Default B: Ventral Prefrontal Cortex 1	3.4	0.004	0.399	0.1	0.74	0.00	13.5	0.001	0.194	4.7	0.034	0.078	1.0	0.312	0.018
Default B: Ventral Prefrontal Cortex 2	3.3	0.005	0.390	0.6	0.44	0.01	8.9	0.004	0.137	3.5	0.068	0.058	2.1	0.151	0.036
Default C: RetroSuperior Parietal Lobuleenial 1	2.4	0.024	0.320	0.6	0.45	0.01	6.6	0.013	0.106	5.6	0.021	0.091	1.3	0.266	0.022
Default C: Parahippocampal Cortex 1	2.7	0.015	0.343	2.3	0.14	0.04	6.3	0.015	0.101	1.7	0.198	0.029	1.5	0.226	0.026
Temporal Parietal 1	3.1	0.006	0.381	7.2	0.01	0.11	5.1	0.027	0.084	4.1	0.048	0.068	1.1	0.298	0.019

	Diastolic BP			Resting Heart Rate			BMI			Sex			Years of Education			Medications			Country of Birth		
	F	p	η^2_p	F	p	η^2_p	F	p	η^2_p	F	p	η^2_p	F	p	η^2_p	F	p	η^2_p	F	p	η^2_p
Visual Central: Extra Striate Cortex 1	1.4	0.250	0.024	1.3	0.263	0.022	0.1	0.778	0.001	0.1	0.823	0.001	1.9	0.168	0.034	0.5	0.469	0.01	0.1	0.796	0.001
Visual Central: Extra Striate Cortex 2	1.0	0.328	0.017	1.2	0.281	0.021	0.1	0.775	0.001	0.1	0.746	0.002	1.3	0.259	0.023	1.1	0.301	0.02	0.2	0.628	0.004
Visual Central: Striate Cortex 1	1.2	0.286	0.020	2.6	0.112	0.044	0.0	0.981	0.000	0.0	0.832	0.001	0.7	0.423	0.011	0.3	0.578	0.01	0.3	0.617	0.005
Visual Central: Extra Striate Cortex 3	1.7	0.192	0.030	1.1	0.290	0.020	0.1	0.747	0.002	0.1	0.754	0.002	1.0	0.325	0.017	1.2	0.277	0.02	0.7	0.423	0.012
Visual Peripheral: Extra Striate Inferior 1	2.1	0.154	0.036	1.7	0.199	0.029	0.0	0.962	0.000	0.2	0.691	0.003	0.5	0.504	0.008	0.6	0.429	0.01	0.3	0.602	0.005
Visual Peripheral: Striate Cortex Calcarine 1	1.7	0.202	0.029	1.7	0.197	0.030	0.0	0.929	0.000	0.1	0.749	0.002	0.9	0.349	0.016	0.2	0.654	0.00	0.2	0.649	0.004
Visual Peripheral: Extra Striate CortexSup 1	1.8	0.180	0.032	1.6	0.218	0.027	0.1	0.810	0.001	0.3	0.608	0.005	0.1	0.705	0.003	0.7	0.419	0.01	0.1	0.727	0.002
Somatomotor A: 1	5.3	0.025	0.086	2.9	0.097	0.049	0.2	0.695	0.003	0.0	0.839	0.001	0.8	0.361	0.015	0.4	0.511	0.01	1.5	0.229	0.026
Somatomotor A: 2	5.3	0.025	0.087	2.0	0.159	0.035	0.2	0.676	0.003	0.4	0.511	0.008	0.7	0.415	0.012	0.1	0.721	0.00	1.5	0.228	0.026
Somatomotor B: Auditory 1	2.0	0.168	0.034	0.3	0.571	0.006	0.4	0.551	0.006	0.0	0.918	0.000	1.6	0.209	0.028	0.9	0.349	0.02	0.0	0.885	0.000
Somatomotor B: S2 1	2.6	0.115	0.044	1.1	0.301	0.019	0.2	0.676	0.003	0.3	0.587	0.005	0.8	0.386	0.013	0.6	0.454	0.01	0.2	0.682	0.003
Somatomotor B: S2 2	1.8	0.191	0.030	0.0	0.836	0.001	0.8	0.369	0.014	1.0	0.328	0.017	0.9	0.336	0.017	0.8	0.384	0.01	0.0	0.883	0.000
Somatomotor B: Central 1	1.5	0.222	0.027	0.5	0.471	0.009	0.1	0.773	0.002	0.0	0.992	0.000	0.7	0.418	0.012	0.9	0.337	0.02	0.9	0.359	0.015
Dorsal Attention A: Temporal Occipital 1	1.3	0.253	0.023	1.9	0.177	0.032	0.0	0.987	0.000	0.0	0.938	0.000	1.4	0.246	0.024	2.2	0.142	0.04	0.0	0.919	0.000
Dorsal Attention A: Parietal Occipital 1	1.3	0.263	0.022	0.1	0.755	0.002	0.9	0.347	0.016	0.2	0.679	0.003	1.9	0.169	0.034	1.3	0.254	0.02	0.0	0.924	0.000
Dorsal Attention A: Superior Parietal Lobule 1	3.3	0.073	0.056	1.4	0.247	0.024	0.0	0.899	0.000	0.0	0.992	0.000	0.7	0.398	0.013	1.0	0.323	0.02	0.6	0.429	0.011
Dorsal Attention B: Post Central 1	0.8	0.366	0.015	0.6	0.453	0.010	0.5	0.493	0.008	0.1	0.704	0.003	1.1	0.304	0.019	0.6	0.432	0.01	0.7	0.408	0.012
Dorsal Attention B: Post Central 2	1.0	0.312	0.018	1.3	0.265	0.022	1.7	0.203	0.029	0.0	0.892	0.000	1.6	0.217	0.027	2.4	0.128	0.04	1.2	0.287	0.020
Dorsal Attention B: Post Central 3	3.6	0.065	0.060	0.4	0.507	0.008	0.0	0.889	0.000	0.0	0.985	0.000	0.9	0.360	0.015	0.9	0.359	0.02	1.1	0.307	0.019
Dorsal Attention B: Frontal Eye Fields 1	3.7	0.059	0.062	1.5	0.229	0.026	0.0	0.919	0.000	0.0	0.921	0.000	0.4	0.517	0.008	0.1	0.719	0.00	2.1	0.150	0.037
Salience Ventral Attention A: Parietal Operculum 1	1.6	0.211	0.028	0.5	0.492	0.008	0.6	0.428	0.011	0.0	0.914	0.000	1.7	0.201	0.029	1.2	0.279	0.02	0.0	0.889	0.000
Salience Ventral Attention A: Insula: 1	1.6	0.212	0.028	0.5	0.489	0.009	0.0	0.961	0.000	0.3	0.597	0.005	1.0	0.313	0.018	0.4	0.524	0.01	0.4	0.547	0.007
Salience Ventral Attention A: Insula: 2	1.7	0.197	0.030	0.4	0.552	0.006	0.2	0.673	0.003	1.4	0.235	0.025	3.0	0.086	0.052	0.2	0.632	0.00	0.2	0.675	0.003
Salience Ventral Attention A: Parietal Medial 1	3.7	0.059	0.062	1.2	0.282	0.021	0.7	0.418	0.012	1.3	0.268	0.022	0.3	0.612	0.005	0.1	0.716	0.00	0.8	0.364	0.015
Salience Ventral Attention A: Frontal Medial 1	3.5	0.065	0.059	1.7	0.199	0.029	0.1	0.806	0.001	0.8	0.361	0.015	0.4	0.556	0.006	0.2	0.691	0.00	1.3	0.265	0.022
Salience Ventral Attention B: Lateral Prefrontal Cor	3.1	0.085	0.052	0.8	0.390	0.013	0.0	0.855	0.001	0.0	0.960	0.000	0.6	0.426	0.011	0.6	0.437	0.01	0.6	0.430	0.011
Salience Ventral Attention B: Medial Posterior Prefr	2.1	0.155	0.036	1.5	0.234	0.025	0.0	0.941	0.000	0.4	0.543	0.007	1.1	0.304	0.019	0.3	0.612	0.00	0.6	0.448	0.010
Limbic A Temporal Pole 1	1.1	0.298	0.019	0.6	0.442	0.011	0.2	0.653	0.004	0.3	0.596	0.005	0.6	0.458	0.010	1.0	0.318	0.02	0.0	0.903	0.000
Limbic A: Temporal Pole 2	1.3	0.261	0.023	0.4	0.555	0.006	0.0	0.927	0.000	1.9	0.179	0.032	1.0	0.317	0.018	1.0	0.325	0.02	0.2	0.648	0.004
Limbic B: Orbital Frontal Cortex 1	2.0	0.167	0.034	0.5	0.475	0.009	0.3	0.575	0.006	0.2	0.621	0.004	1.5	0.226	0.026	1.8	0.187	0.03	0.0	0.894	0.000
Control A: Intraparietal Sulcus 1	4.6	0.037	0.075	1.2	0.269	0.022	0.5	0.477	0.009	0.0	0.860	0.001	2.1	0.155	0.036	0.8	0.367	0.01	1.7	0.193	0.030
Control A: Lateral Prefrontal Cortex 1	2.0	0.165	0.034	0.6	0.454	0.010	0.5	0.497	0.008	0.7	0.408	0.012	1.6	0.204	0.029	0.6	0.455	0.01	0.3	0.584	0.005
Control A: Lateral Prefrontal Cortex 2	1.3	0.252	0.023	0.1	0.785	0.001	0.3	0.586	0.005	1.5	0.224	0.026	1.7	0.199	0.029	0.4	0.527	0.01	0.3	0.592	0.005
Control B: Lateral Prefrontal Cortexv 1	2.2	0.148	0.037	0.8	0.370	0.014	0.7	0.393	0.013	0.1	0.760	0.002	0.8	0.372	0.014	0.3	0.581	0.01	0.4	0.538	0.007
Control C: Precuneus 1	3.6	0.064	0.060	2.2	0.144	0.038	0.0	0.997	0.000	0.1	0.733	0.002	0.5	0.473	0.009	0.7	0.416	0.01	0.9	0.336	0.017
Control C: Precuneus 2	1.7	0.192	0.030	0.7	0.401	0.013	0.1	0.715	0.002	1.1	0.298	0.019	1.2	0.276	0.021	1.0	0.315	0.02	0.2	0.674	0.003
Control C: Cingulate Posterior 1	0.7	0.409	0.012	0.3	0.582	0.005	0.2	0.639	0.004	0.6	0.427	0.011	0.5	0.474	0.009	1.0	0.319	0.02	0.0	1.000	0.000
Default A: Dorsal Prefrontal Cortex 1	3.2	0.079	0.054	0.9	0.348	0.016	0.3	0.570	0.006	0.2	0.684	0.003	1.0	0.332	0.017	0.6	0.426	0.01	2.4	0.124	0.042
Default A: Precuneus Posterior Cingulate Cortex1	1.2	0.268	0.022	0.5	0.462	0.010	0.1	0.734	0.002	0.2	0.640	0.004	0.5	0.476	0.009	0.6	0.461	0.01	0.1	0.822	0.001
Default A: Medial Prefrontal Cortex 1	1.5	0.225	0.026	0.8	0.383	0.014	0.2	0.622	0.004	0.3	0.588	0.005	0.9	0.351	0.016	0.2	0.678	0.00	0.2	0.642	0.004
Default B: Temp 1	1.2	0.273	0.021	0.5	0.480	0.009	0.2	0.650	0.004	0.4	0.543	0.007	1.2	0.269	0.022	1.0	0.311	0.02	0.0	0.919	0.000
Default B: Temp 2	2.0	0.165	0.034	0.9	0.348	0.016	0.5	0.498	0.008	0.1	0.743	0.002	2.3	0.134	0.040	2.5	0.120	0.04	0.0	0.899	0.000
Default B: Inferior Parietal Lobule 1	2.5	0.117	0.043	0.5	0.475	0.009	0.5	0.491	0.008	0.0	0.873	0.000	1.2	0.277	0.021	1.3	0.255	0.02	0.7	0.422	0.012
Default B: Dorsal Prefrontal Cortex 1	2.2	0.140	0.038	1.0	0.333	0.017	0.2	0.676	0.003	0.3	0.611	0.005	1.4	0.241	0.024	0.4	0.513	0.01	0.9	0.335	0.017
Default B: Lateral Prefrontal Cortex 1	2.0	0.167	0.034	1.1	0.309	0.019	0.3	0.604	0.005	0.0	0.943	0.000	1.2	0.281	0.021	1.5	0.228	0.03	0.3	0.566	0.006
Default B: Ventral Prefrontal Cortex 1	1.2	0.268	0.022	0.5	0.493	0.008	0.0	0.987	0.000	0.2	0.620	0.004	1.1	0.302	0.019	0.7	0.396	0.01	0.0	0.858	0.001
Default B: Ventral Prefrontal Cortex 2	2.0	0.159	0.035	1.1	0.309	0.018	0.2	0.643	0.004	0.1	0.710	0.002	1.4	0.245	0.024	0.8	0.369	0.01	0.2	0.667	0.003
Default C: RetroSuperior Parietal Lobuleenial 1	1.5	0.230	0.026	0.1	0.736	0.002	0.0	0.857	0.001	0.8	0.382	0.014	1.5	0.232	0.025	0.8	0.386	0.01	0.0	0.875	0.000
Default C: Parahippocampal Cortex 1	2.2	0.141	0.038	0.2	0.697	0.003	0.3	0.609	0.005	0.9	0.341	0.016	2.3	0.139	0.039	0.3	0.591	0.01	0.4	0.520	0.007
Temporal Parietal 1	1.9	0.179	0.032	0.8	0.369	0.014	0.7	0.411	0.012	0.1	0.751	0.002	2.5	0.121	0.042	1.3	0.260	0.02	0.2	0.645	0.004

continued …

… Supplementary Table 10 continued

	Overall Model			Cortical Thickness			Age Group			HOMA-IR			Systolic BP			Diastolic BP			Resting Heart Rate			BMI			Sex			Years of Education			Medications			Country of Birth		
	F	p-FDR	η^2_p	F	p	η^2_p	F	p	η^2_p	F	p	η^2_p	F	p	η^2_p	F	p	η^2_p	F	p	η^2_p	F	p	η^2_p	F	p	η^2_p	F	p	η^2_p	F	p	η^2_p	F	p	η^2_p
Visual Central: Extra Striate Cortex 1	1.3	0.271	0.206	1.6	0.21	0.03	2.7	0.107	0.046	3.7	0.058	0.063	2.4	0.124	0.042	2.9	0.095	0.049	2.3	0.136	0.039	0.0	0.854	0.001	0.0	0.830	0.001	0.8	0.362	0.015	1.0	0.315	0.02	0.0	0.955	0.000
Visual Central: Extra Striate Cortex 2	1.0	0.539	0.158	0.1	0.71	0.00	3.2	0.081	0.053	3.9	0.055	0.064	0.8	0.366	0.015	0.8	0.386	0.013	1.3	0.267	0.022	0.0	0.970	0.000	0.0	0.860	0.001	0.8	0.363	0.015	1.2	0.285	0.02	0.2	0.658	0.004
Visual Central: Extra Striate Cortex 3	1.2	0.327	0.195	2.0	0.16	0.03	2.2	0.144	0.038	3.9	0.053	0.065	0.8	0.366	0.015	0.7	0.421	0.012	0.8	0.368	0.014	0.0	0.884	0.000	0.0	0.998	0.000	0.4	0.519	0.007	1.0	0.311	0.02	0.0	0.870	0.000
Visual Peripheral: Striate Cortex Calcarine 1	1.0	0.514	0.163	0.2	0.65	0.00	3.4	0.071	0.057	2.0	0.165	0.034	0.8	0.363	0.015	1.4	0.249	0.024	2.4	0.129	0.041	0.0	0.874	0.000	0.0	0.962	0.000	0.2	0.655	0.004	0.3	0.568	0.01	0.2	0.633	0.004
Visual Peripheral: Extra Striate Inferior 1	2.5	0.020	0.329	4.8	0.03	0.08	4.4	0.041	0.073	3.7	0.060	0.062	2.6	0.112	0.044	3.6	0.064	0.060	2.0	0.161	0.035	0.0	0.946	0.000	0.1	0.748	0.002	1.0	0.332	0.017	0.1	0.725	0.00	0.6	0.461	0.010
Visual Peripheral: Extra Striate Superior 1	1.9	0.077	0.269	3.4	0.07	0.06	5.0	0.029	0.083	4.9	0.031	0.080	2.2	0.145	0.038	2.8	0.101	0.047	3.1	0.086	0.052	0.1	0.738	0.002	0.1	0.743	0.002	0.1	0.732	0.002	1.1	0.299	0.02	0.6	0.439	0.011
Somatomotor A: 1	2.6	0.018	0.334	2.5	0.12	0.04	7.6	0.008	0.119	4.8	0.032	0.079	1.4	0.246	0.024	1.9	0.176	0.032	0.8	0.366	0.015	0.1	0.810	0.001	0.1	0.755	0.002	1.2	0.281	0.021	0.3	0.569	0.01	0.3	0.563	0.006
Somatomotor A: 2	2.6	0.015	0.341	6.7	0.01	0.11	6.6	0.013	0.105	7.2	0.010	0.113	1.7	0.198	0.029	2.6	0.110	0.045	1.6	0.205	0.029	0.0	0.979	0.000	0.0	0.940	0.000	1.4	0.234	0.025	0.7	0.416	0.01	0.5	0.468	0.009
Somatomotor A: 3	2.5	0.019	0.332	5.2	0.03	0.08	5.0	0.029	0.082	4.7	0.035	0.077	0.9	0.356	0.015	2.0	0.161	0.035	0.4	0.509	0.008	0.0	0.996	0.000	0.0	0.997	0.000	0.7	0.412	0.012	0.2	0.635	0.00	0.9	0.359	0.015
Somatomotor A: 4	3.6	0.003	0.417	8.1	0.01	0.13	6.8	0.012	0.108	5.0	0.029	0.082	3.9	0.054	0.065	5.9	0.018	0.095	1.7	0.193	0.030	0.3	0.589	0.005	0.7	0.401	0.013	1.4	0.248	0.024	0.0	0.872	0.00	2.7	0.103	0.047
Somatomotor B: Auditory 1	3.4	0.004	0.398	9.6	0.00	0.15	2.7	0.106	0.046	4.4	0.041	0.072	1.8	0.186	0.031	2.7	0.107	0.046	0.4	0.538	0.007	0.2	0.657	0.004	0.1	0.731	0.002	1.0	0.316	0.018	0.6	0.428	0.01	1.2	0.277	0.021
Somatomotor B: S2 1	2.6	0.018	0.335	1.0	0.32	0.02	4.9	0.032	0.080	1.9	0.169	0.034	0.8	0.364	0.015	1.3	0.256	0.023	0.5	0.486	0.009	0.9	0.336	0.017	0.8	0.378	0.014	1.0	0.332	0.017	0.6	0.429	0.01	0.0	0.895	0.000
Somatomotor B: S2 2	3.2	0.005	0.389	2.4	0.13	0.04	8.8	0.005	0.135	2.8	0.100	0.048	2.3	0.134	0.040	2.4	0.131	0.040	0.2	0.669	0.003	0.5	0.483	0.009	0.8	0.362	0.015	0.4	0.554	0.006	0.1	0.711	0.00	0.4	0.512	0.008
Somatomotor B: Central 1	2.0	0.057	0.285	6.5	0.01	0.10	3.4	0.069	0.058	3.5	0.068	0.058	2.6	0.110	0.045	2.5	0.121	0.042	0.7	0.419	0.012	0.1	0.708	0.003	0.2	0.637	0.004	0.6	0.432	0.011	0.3	0.557	0.01	1.1	0.290	0.020
Dorsal Attention A: Temporal Occipital 1	1.5	0.186	0.228	0.8	0.38	0.01	6.5	0.014	0.104	2.8	0.097	0.048	2.0	0.162	0.035	2.3	0.137	0.039	0.9	0.355	0.015	0.1	0.744	0.002	0.0	0.939	0.000	1.1	0.305	0.019	1.3	0.268	0.02	0.3	0.598	0.005
Dorsal Attention A: Parietal Occipital 1	1.9	0.067	0.276	2.6	0.12	0.04	4.5	0.039	0.074	2.6	0.113	0.044	2.5	0.117	0.043	2.7	0.106	0.046	1.0	0.326	0.017	0.7	0.404	0.012	0.0	0.883	0.000	0.7	0.416	0.012	1.3	0.254	0.02	0.3	0.584	0.005
Dorsal Attention A: Superior Parietal Lobule 1	3.4	0.004	0.399	8.5	0.01	0.13	8.8	0.004	0.136	6.5	0.014	0.104	2.3	0.135	0.039	3.9	0.053	0.065	1.2	0.281	0.021	0.0	0.987	0.000	0.0	0.937	0.000	1.1	0.294	0.020	2.4	0.127	0.04	0.5	0.462	0.010
Dorsal Attention B: Post Central 1	3.0	0.008	0.369	1.9	0.18	0.03	4.0	0.050	0.067	4.8	0.032	0.079	0.7	0.392	0.013	2.0	0.161	0.035	1.2	0.284	0.020	0.1	0.732	0.002	0.1	0.731	0.002	0.0	0.832	0.001	0.9	0.336	0.02	1.8	0.189	0.031
Dorsal Attention B: Post Central 2	3.4	0.004	0.401	12.4	0.00	0.18	2.5	0.121	0.042	3.7	0.060	0.062	0.7	0.417	0.012	2.2	0.140	0.038	1.2	0.270	0.022	0.2	0.624	0.004	0.0	0.902	0.000	0.2	0.659	0.004	0.7	0.400	0.01	2.9	0.096	0.049
Dorsal Attention B: Frontal Eye Fields 1	6.0	0.000	0.540	19.3	0.00	0.26	12.0	0.001	0.177	4.6	0.035	0.077	6.8	0.011	0.109	6.3	0.015	0.101	3.3	0.074	0.056	0.0	0.826	0.001	0.3	0.595	0.005	0.6	0.427	0.011	0.6	0.430	0.01	2.9	0.096	0.049
Salience Ventral Attention A: Parietal Operculum 1	3.3	0.005	0.392	0.7	0.40	0.01	9.1	0.004	0.140	3.9	0.053	0.065	1.0	0.325	0.017	1.6	0.206	0.028	0.2	0.637	0.004	0.8	0.365	0.015	0.0	0.886	0.000	0.3	0.585	0.005	0.5	0.469	0.01	0.4	0.550	0.006
Salience Ventral Attention A: Insula: 1	3.0	0.007	0.372	0.1	0.80	0.00	8.9	0.004	0.137	3.1	0.083	0.053	1.7	0.201	0.029	2.2	0.141	0.038	0.7	0.417	0.012	0.1	0.714	0.002	0.7	0.399	0.013	0.8	0.379	0.014	0.9	0.353	0.02	0.2	0.689	0.003
Salience Ventral Attention A: Parietal Medial 1	3.7	0.003	0.418	12.7	0.00	0.19	2.4	0.124	0.042	3.2	0.079	0.054	1.5	0.227	0.026	2.1	0.149	0.037	0.1	0.701	0.003	0.1	0.764	0.002	1.0	0.311	0.018	0.7	0.408	0.012	0.6	0.443	0.01	0.1	0.759	0.002
Salience Ventral Attention A: Frontal Medial 1	4.1	0.002	0.447	4.4	0.04	0.07	3.0	0.087	0.051	2.2	0.142	0.038	0.8	0.378	0.014	2.1	0.153	0.036	1.4	0.241	0.024	0.4	0.549	0.006	1.1	0.303	0.019	0.6	0.438	0.011	0.0	0.982	0.00	1.5	0.232	0.025
Salience Ventral Attention B: Inferior Parietal Lobul	2.9	0.008	0.364	1.2	0.28	0.02	9.2	0.004	0.141	2.7	0.108	0.045	0.6	0.435	0.011	0.8	0.366	0.015	0.3	0.600	0.005	0.7	0.408	0.012	0.1	0.775	0.001	0.6	0.448	0.010	1.1	0.289	0.02	0.4	0.515	0.008
Salience Ventral Attention B: Lateral Prefrontal Cor	3.7	0.003	0.418	2.2	0.14	0.04	9.6	0.003	0.146	2.6	0.114	0.044	2.0	0.159	0.035	2.7	0.107	0.046	1.0	0.332	0.017	0.2	0.619	0.004	0.1	0.743	0.002	0.7	0.394	0.013	0.1	0.737	0.00	1.0	0.329	0.017
Salience Ventral Attention B: Medial Posterior Prefr	4.7	0.001	0.481	0.5	0.50	0.01	12.7	0.001	0.185	2.4	0.129	0.041	1.7	0.198	0.029	2.4	0.129	0.041	1.9	0.171	0.033	0.2	0.626	0.004	1.3	0.254	0.023	1.0	0.317	0.018	0.0	0.970	0.00	1.0	0.329	0.017
Limbic A: Temporal Pole 1	2.4	0.022	0.324	0.0	0.90	0.00	11.8	0.001	0.174	2.7	0.106	0.046	1.9	0.176	0.032	1.8	0.182	0.032	0.9	0.358	0.015	0.3	0.561	0.006	0.2	0.672	0.003	0.7	0.403	0.013	1.4	0.248	0.02	0.0	0.978	0.000
Limbic B: Orbital Frontal Cortex 1	2.7	0.015	0.343	1.5	0.22	0.03	7.7	0.008	0.120	2.9	0.093	0.050	1.0	0.321	0.018	1.6	0.214	0.027	0.6	0.453	0.010	0.1	0.704	0.003	1.1	0.306	0.019	0.6	0.438	0.011	1.5	0.222	0.03	0.0	0.829	0.001
Control A: Intraparietal Sulcus 1	2.3	0.033	0.307	3.5	0.07	0.06	4.3	0.042	0.072	2.2	0.140	0.038	1.1	0.299	0.019	1.7	0.191	0.030	0.1	0.701	0.003	0.1	0.815	0.001	0.0	0.875	0.000	1.6	0.210	0.028	0.6	0.438	0.01	0.9	0.351	0.016
Control A: Lateral Prefrontal Cortex 1	2.9	0.009	0.362	0.0	0.95	0.00	13.3	0.001	0.192	2.9	0.092	0.050	1.2	0.276	0.021	1.8	0.183	0.031	0.4	0.541	0.007	0.4	0.508	0.008	0.2	0.673	0.003	0.8	0.375	0.014	0.7	0.414	0.01	0.5	0.479	0.009
Control A: Lateral Prefrontal Cortex 2	3.1	0.006	0.377	2.1	0.15	0.04	10.6	0.002	0.159	2.8	0.101	0.047	2.8	0.100	0.048	2.5	0.121	0.042	0.5	0.474	0.009	0.4	0.544	0.007	0.1	0.770	0.002	0.7	0.392	0.013	0.7	0.423	0.01	1.0	0.330	0.017
Control B: Temporal 1	2.5	0.020	0.328	0.0	0.99	0.00	11.0	0.002	0.165	4.0	0.051	0.066	2.0	0.159	0.035	2.0	0.158	0.035	0.7	0.403	0.013	0.2	0.626	0.004	0.4	0.545	0.007	0.6	0.448	0.010	1.2	0.273	0.02	0.1	0.741	0.002
Control B: inferior parietal lobule 1	3.6	0.003	0.418	7.8	0.01	0.12	6.0	0.018	0.096	3.5	0.068	0.058	2.0	0.162	0.035	2.6	0.111	0.045	2.0	0.158	0.035	0.7	0.400	0.013	0.1	0.818	0.001	0.9	0.351	0.016	1.1	0.307	0.02	0.2	0.692	0.003
Control B: Lateral Prefrontal Cortexd 1	3.8	0.002	0.427	5.7	0.02	0.09	5.8	0.019	0.094	3.8	0.058	0.063	1.3	0.256	0.023	2.1	0.154	0.036	0.7	0.396	0.013	0.4	0.518	0.008	0.0	0.843	0.001	1.4	0.247	0.024	0.4	0.551	0.01	0.6	0.443	0.011
Control B: Lateral Prefrontal Cortexv 1	3.0	0.007	0.372	1.2	0.27	0.02	9.9	0.003	0.150	4.0	0.052	0.066	1.7	0.200	0.029	1.6	0.213	0.028	1.1	0.299	0.019	0.2	0.658	0.004	0.1	0.737	0.002	0.1	0.748	0.002	0.8	0.374	0.01	0.4	0.514	0.008
Control C: Cingulate Posterior 1	2.5	0.020	0.329	1.9	0.18	0.03	5.4	0.024	0.088	3.4	0.071	0.057	1.8	0.184	0.031	2.3	0.136	0.039	0.9	0.334	0.017	0.0	0.833	0.001	0.6	0.434	0.011	0.8	0.379	0.014	0.0	0.996	0.00	0.1	0.775	0.001
Control C: Precuneus 1	2.3	0.030	0.312	2.1	0.15	0.04	6.0	0.018	0.096	4.3	0.043	0.071	2.8	0.101	0.047	3.3	0.074	0.056	1.2	0.273	0.021	0.1	0.807	0.001	0.4	0.510	0.008	0.1	0.753	0.002	0.9	0.353	0.02	0.2	0.640	0.004
Default A: Inferior Parietal Lobule 1	3.4	0.004	0.398	8.6	0.00	0.13	2.7	0.107	0.046	3.9	0.054	0.064	1.9	0.179	0.032	1.9	0.171	0.033	1.7	0.202	0.029	0.8	0.362	0.015	0.1	0.738	0.002	0.3	0.580	0.006	1.2	0.274	0.02	1.4	0.243	0.024
Default A: Dorsal Prefrontal Cortex 1	4.2	0.002	0.452	5.1	0.03	0.08	10.5	0.002	0.158	2.8	0.099	0.048	3.3	0.077	0.055	3.5	0.067	0.059	3.1	0.086	0.052	0.2	0.650	0.004	0.2	0.667	0.003	0.7	0.402	0.013	0.3	0.593	0.01	0.6	0.426	0.011
Default A: Precuneus Posterior Cingulate Cortex 1	1.9	0.080	0.267	0.7	0.42	0.01	5.9	0.019	0.095	4.3	0.043	0.071	1.9	0.179	0.032	2.4	0.127	0.041	0.7	0.405	0.012	0.1	0.797	0.001	0.2	0.656	0.004	0.3	0.567	0.006	1.2	0.278	0.02	0.1	0.766	0.002
Default A: Medial Prefrontal Cortex 1	4.1	0.002	0.446	0.0	0.86	0.00	14.2	0.000	0.202	3.5	0.066	0.059	0.7	0.395	0.013	1.1	0.307	0.019	0.8	0.369	0.014	0.5	0.478	0.009	0.3	0.610	0.005	1.2	0.272	0.021	0.5	0.462	0.01	0.2	0.698	0.003
Default B: Dorsal Prefrontal Cortex 1	5.2	0.001	0.504	4.0	0.05	0.07	11.3	0.001	0.168	3.3	0.075	0.055	2.9	0.094	0.049	3.1	0.083	0.053	0.9	0.352	0.015	0.3	0.563	0.006	0.3	0.609	0.005	1.0	0.322	0.017	0.8	0.386	0.01	0.6	0.442	0.011
Default B: Ventral Prefrontal Cortex 1	3.0	0.007	0.374	1.7	0.20	0.03	9.8	0.003	0.149	4.3	0.043	0.071	2.0	0.161	0.035	2.5	0.122	0.042	0.8	0.387	0.013	0.1	0.744	0.002	0.2	0.661	0.003	0.1	0.821	0.001	0.9	0.351	0.02	0.2	0.657	0.004
Default B: Ventral Prefrontal Cortex 2	3.5	0.003	0.410	1.6	0.22	0.03	13.4	0.001	0.193	3.1	0.083	0.053	2.5	0.119	0.043	1.7	0.200	0.029	0.7	0.409	0.012	0.1	0.781	0.001	0.5	0.488	0.009	0.7	0.413	0.012	1.3	0.268	0.02	0.5	0.501	0.008
Default C: RetroSuperior Parietal Lobuleenial 1	1.9	0.066	0.277	1.7	0.19	0.03	2.8	0.101	0.047	3.2	0.080	0.054	1.6	0.215	0.027	2.2	0.140	0.039	0.9	0.356	0.015	0.0	0.852	0.001	0.9	0.354	0.015	0.2	0.695	0.003	0.4	0.526	0.01	0.1	0.718	0.002
Default C: Parahippocampal Cortex 1	1.9	0.067	0.276	1.7	0.20	0.03	3.3	0.075	0.056	2.6	0.115	0.044	0.9	0.348	0.016	2.0	0.160	0.035	1.1	0.289	0.020	0.1	0.799	0.001	0.6	0.431	0.011	0.6	0.426	0.011	0.6	0.445	0.01	0.0	0.848	0.001
Temporal Parietal 1	3.2	0.005	0.386	4.7	0.03	0.08	9.1	0.004	0.140	2.5	0.117	0.043	1.4	0.235	0.025	1.7	0.192	0.030	0.2	0.685	0.003	0.0	0.887	0.000	0.4	0.532	0.007	0.4	0.546	0.007	1.8	0.185	0.03	0.2	0.629	0.004
Temporal Parietal 2	3.0	0.007	0.371	3.4	0.07	0.06	4.1	0.047	0.068	4.7	0.035	0.077	1.0	0.320	0.018	2.0	0.161	0.035	1.0	0.330	0.017	0.3	0.591	0.005	0.0	0.870	0.000	0.5	0.498	0.008	0.8	0.366	0.01	0.3	0.580	0.006
Temporal Parietal 3	2.1	0.047	0.293	3.5	0.07	0.06	3.7	0.059	0.062	1.8	0.188	0.031	1.2	0.276	0.021	1.2	0.279	0.021	0.4	0.538	0.007	0.5	0.490	0.009	0.1	0.717	0.002	0.7	0.392	0.013	1.5	0.227	0.03	0.1	0.761	0.002

Supplementary Table 11. GLMs of age group, HOMA-IR2 and age group x HOMA-IR2 effect on regional CMR_{GLC}, including cortical thickness as a covariate.

Left Hemisphere

	Overall Model			Cortial Thickness			Age Category			HOMA-IR2			Age Group x HOMA-IR2		
	F	p-FDR	η^2_p	F	p	η^2_p	F	p	η^2_p	F	p	η^2_p	F	p	η^2_p
Visual Central: Extra Striate Cortex 1	2.5	0.053	0.117	1.1	0.299	0.015	4.4	0.039	0.056	3.7	0.057	0.048	2.4	0.127	0.031
Visual Central: Extra Striate Cortex 2	3.0	0.024	0.139	0.2	0.649	0.003	5.7	0.020	0.071	6.8	0.011	0.084	3.6	0.062	0.046
Visual Central: Striate Cortex 1	3.0	0.023	0.141	0.2	0.624	0.003	6.6	0.012	0.082	5.4	0.023	0.068	2.6	0.110	0.034
Visual Central: Extra Striate Cortex 3	4.9	0.002	0.209	1.2	0.283	0.016	10.5	0.002	0.124	5.5	0.022	0.069	6.2	0.015	0.077
Visual Peripheral: Extra Striate Inferior 1	4.5	0.003	0.195	0.5	0.497	0.006	9.7	0.003	0.116	4.1	0.046	0.053	3.5	0.067	0.045
Visual Peripheral: Striate Cortex Calcarine 1	7.3	0.000	0.283	9.2	0.003	0.111	9.6	0.003	0.115	7.9	0.006	0.097	5.6	0.020	0.071
Visual Peripheral: Extra Striate CortexSup 1	7.2	0.000	0.281	2.2	0.147	0.028	14.9	0.000	0.168	8.0	0.006	0.097	6.4	0.013	0.080
Somatomotor A: 1	10.1	0.000	0.353	7.2	0.009	0.089	13.8	0.000	0.158	7.1	0.010	0.087	4.7	0.034	0.059
Somatomotor A: 2	8.7	0.000	0.320	4.9	0.029	0.063	8.4	0.005	0.102	8.4	0.005	0.102	3.6	0.060	0.047
Somatomotor B: Auditory 1	9.7	0.000	0.343	5.6	0.020	0.071	11.5	0.001	0.134	10.8	0.002	0.127	6.2	0.015	0.077
Somatomotor B: S2 1	7.3	0.000	0.283	1.5	0.229	0.020	13.7	0.000	0.156	7.8	0.007	0.096	4.5	0.038	0.057
Somatomotor B: S2 2	13.6	0.000	0.423	3.5	0.064	0.045	22.4	0.000	0.232	8.9	0.004	0.107	6.4	0.014	0.079
Somatomotor B: Central 1	6.8	0.000	0.269	2.6	0.113	0.034	12.3	0.001	0.142	7.3	0.009	0.089	4.6	0.035	0.059
Dorsal Attention A: Temporal Occipital 1	9.7	0.000	0.344	13.6	0.000	0.155	14.2	0.000	0.161	5.3	0.025	0.066	9.0	0.004	0.109
Dorsal Attention A: Parietal Occipital 1	5.9	0.000	0.242	1.0	0.328	0.013	9.8	0.003	0.117	7.1	0.009	0.088	4.4	0.040	0.056
Dorsal Attention A: Superior Parietal Lobule 1	8.1	0.000	0.306	4.8	0.032	0.061	10.6	0.002	0.125	7.1	0.010	0.087	5.1	0.026	0.065
Dorsal Attention B: Post Central 1	7.8	0.000	0.296	0.1	0.732	0.002	14.6	0.000	0.165	7.1	0.009	0.088	2.7	0.104	0.035
Dorsal Attention B: Post Central 2	12.7	0.000	0.407	15.9	0.000	0.177	6.2	0.015	0.077	7.8	0.007	0.095	2.8	0.097	0.037
Dorsal Attention B: Post Central 3	11.7	0.000	0.388	12.0	0.001	0.140	16.0	0.000	0.178	8.0	0.006	0.097	7.5	0.008	0.092
Dorsal Attention B: Frontal Eye Fields 1	11.6	0.000	0.386	3.3	0.074	0.042	16.2	0.000	0.180	6.8	0.011	0.084	4.1	0.046	0.053
Salience Ventral Attention A: Parietal Operculum 1	10.1	0.000	0.354	1.4	0.233	0.019	16.7	0.000	0.184	10.1	0.002	0.120	4.5	0.037	0.057
Salience Ventral Attention A: Insula: 1	12.9	0.000	0.411	1.1	0.307	0.014	22.1	0.000	0.230	8.6	0.005	0.104	4.5	0.036	0.058
Salience Ventral Attention A: Insula: 2	12.2	0.000	0.398	5.3	0.025	0.066	12.8	0.001	0.147	6.4	0.013	0.080	3.6	0.062	0.046
Salience Ventral Attention A: Parietal Medial 1	12.7	0.000	0.408	13.2	0.001	0.152	7.6	0.007	0.093	13.0	0.001	0.149	3.9	0.052	0.050
Salience Ventral Attention A: Frontal Medial 1	12.1	0.000	0.395	7.7	0.007	0.094	10.1	0.002	0.120	6.8	0.011	0.084	4.4	0.038	0.057
Salience Ventral Attention B: Lateral Prefrontal Cortex	16.6	0.000	0.472	14.0	0.000	0.159	14.1	0.000	0.160	10.8	0.002	0.127	3.8	0.056	0.048
Salience Ventral Attention B: Medial Posterior Prefront	11.0	0.000	0.372	1.8	0.188	0.023	11.7	0.001	0.137	8.9	0.004	0.107	1.7	0.197	0.022
Limbic A Temporal Pole 1	8.2	0.000	0.308	0.1	0.703	0.002	15.5	0.000	0.173	9.7	0.003	0.116	2.4	0.125	0.032
Limbic A: Temporal Pole 2	7.8	0.000	0.296	3.6	0.063	0.046	15.4	0.000	0.172	6.6	0.012	0.082	3.9	0.053	0.050
Limbic B: Orbital Frontal Cortex 1	6.2	0.000	0.251	0.4	0.519	0.006	12.9	0.001	0.149	8.3	0.005	0.101	3.3	0.071	0.043
Control A: Intraparietal Sulcus 1	10.5	0.000	0.362	6.1	0.016	0.076	12.7	0.001	0.147	7.1	0.009	0.088	4.7	0.033	0.060
Control A: Lateral Prefrontal Cortex 1	9.8	0.000	0.346	3.2	0.077	0.042	11.6	0.001	0.136	6.5	0.013	0.081	3.0	0.089	0.039
Control A: Lateral Prefrontal Cortex 2	10.4	0.000	0.361	6.6	0.012	0.082	4.9	0.029	0.062	5.1	0.027	0.064	1.5	0.230	0.019
Control B: Lateral Prefrontal Cortexv 1	10.4	0.000	0.359	5.8	0.018	0.073	15.3	0.000	0.171	8.0	0.006	0.098	3.8	0.055	0.049
Control C: Precuneus 1	6.2	0.000	0.250	0.8	0.383	0.010	7.1	0.009	0.088	11.5	0.001	0.135	2.1	0.148	0.028
Control C: Precuneus 2	8.1	0.000	0.305	3.7	0.060	0.047	11.1	0.001	0.130	11.8	0.001	0.138	4.6	0.035	0.058
Control C: Cingulate Posterior 1	6.2	0.000	0.251	1.8	0.178	0.024	8.6	0.004	0.105	8.9	0.004	0.107	3.6	0.063	0.046
Default A: Dorsal Prefrontal Cortex 1	16.6	0.000	0.473	10.5	0.002	0.125	15.0	0.000	0.168	8.0	0.006	0.098	2.8	0.096	0.037
Default A: Precuneus Posterior Cingulate Cortex1	6.7	0.000	0.267	2.8	0.099	0.036	8.1	0.006	0.098	11.1	0.001	0.131	3.2	0.078	0.041
Default A: Medial Prefrontal Cortex 1	12.5	0.000	0.403	1.3	0.253	0.018	19.9	0.000	0.212	8.3	0.005	0.101	2.8	0.100	0.036
Default B: Temp 1	9.4	0.000	0.337	3.1	0.084	0.040	15.3	0.000	0.171	7.3	0.009	0.089	5.0	0.028	0.064
Default B: Temp 2	9.5	0.000	0.338	7.7	0.007	0.094	10.0	0.002	0.119	6.5	0.013	0.081	4.2	0.043	0.054
Default B: Inferior Parietal Lobule 1	9.2	0.000	0.333	4.8	0.032	0.061	8.8	0.004	0.106	10.4	0.002	0.123	5.2	0.025	0.066
Default B: Dorsal Prefrontal Cortex 1	15.3	0.000	0.453	3.9	0.052	0.050	14.9	0.000	0.168	8.3	0.005	0.100	3.1	0.082	0.040
Default B: Lateral Prefrontal Cortex 1	10.8	0.000	0.370	5.8	0.018	0.073	12.5	0.001	0.144	6.6	0.012	0.081	3.3	0.075	0.042
Default B: Ventral Prefrontal Cortex 1	11.4	0.000	0.381	0.0	0.989	0.000	19.5	0.000	0.208	10.1	0.002	0.120	2.2	0.143	0.029
Default B: Ventral Prefrontal Cortex 2	10.3	0.000	0.358	2.5	0.117	0.033	14.6	0.000	0.165	9.7	0.003	0.115	4.1	0.046	0.053
Default C: RetroSuperior Parietal Lobuleenial 1	7.4	0.000	0.286	0.6	0.430	0.008	10.3	0.002	0.122	13.1	0.001	0.150	2.0	0.164	0.026
Default C: Parahippocampal Cortex 1	7.3	0.000	0.284	2.2	0.144	0.029	12.4	0.001	0.143	5.9	0.018	0.074	2.9	0.095	0.037
Temporal Parietal 1	10.1	0.000	0.352	5.1	0.026	0.065	15.4	0.000	0.172	10.6	0.002	0.125	6.9	0.010	0.086
Caudate	5.8	0.000	0.238	2.8	0.098	0.037	6.6	0.012	0.081	5.8	0.019	0.073	2.8	0.100	0.036
Putamen	5.7	0.001	0.237	1.4	0.237	0.019	7.2	0.009	0.089	3.8	0.054	0.049	1.5	0.224	0.020
Pallidum	5.7	0.001	0.235	0.8	0.362	0.011	5.8	0.018	0.073	5.4	0.023	0.068	0.6	0.457	0.007
Thalamus	5.6	0.001	0.231	1.4	0.238	0.019	4.0	0.048	0.052	9.2	0.003	0.111	0.6	0.438	0.008

Right Hemisphere

	Overall Model			Cortical Thickness			Age Category			HOMA-IR2			Age Group x HOMA-IR2		
	F	p-FDR	η^2_p	F	p	η^2_p	F	p	η^2_p	F	p	η^2_p	F	p	η^2_p
Visual Central: Extra Striate Cortex 1	8.2	0.000	0.306	1.9	0.168	0.026	12.9	0.001	0.148	9.5	0.003	0.113	2.9	0.094	0.037
Visual Central: Extra Striate Cortex 2	8.1	0.000	0.305	5.8	0.019	0.072	14.8	0.000	0.167	8.7	0.004	0.105	5.4	0.023	0.068
Visual Central: Extra Striate Cortex 3	8.6	0.000	0.317	7.0	0.010	0.087	10.0	0.002	0.119	8.3	0.005	0.101	3.4	0.068	0.044
Visual Peripheral: Striate Cortex Calcarine 1	8.9	0.000	0.324	3.6	0.061	0.047	9.5	0.003	0.114	9.8	0.002	0.117	3.5	0.065	0.045
Visual Peripheral: Extra Striate Inferior 1	11.8	0.000	0.390	9.9	0.002	0.118	11.4	0.001	0.134	12.9	0.001	0.149	8.8	0.004	0.106
Visual Peripheral: Extra Striate Superior 1	7.9	0.000	0.299	0.8	0.366	0.011	14.3	0.000	0.162	8.2	0.005	0.100	3.1	0.082	0.040
Somatomotor A: 1	11.9	0.000	0.391	3.3	0.071	0.043	19.4	0.000	0.208	11.0	0.001	0.129	5.8	0.018	0.073
Somatomotor A: 2	6.1	0.000	0.247	2.8	0.101	0.036	8.1	0.006	0.099	7.9	0.006	0.097	4.0	0.048	0.052
Somatomotor A: 3	5.2	0.001	0.220	0.5	0.481	0.007	12.3	0.001	0.143	7.4	0.008	0.091	6.0	0.017	0.075
Somatomotor A: 4	7.4	0.000	0.286	2.4	0.129	0.031	13.5	0.000	0.154	8.1	0.006	0.099	7.5	0.008	0.092
Somatomotor B: Auditory 1	9.3	0.000	0.334	4.7	0.034	0.060	13.9	0.000	0.158	11.7	0.001	0.137	6.0	0.017	0.075
Somatomotor B: S2 1	8.0	0.000	0.301	2.2	0.145	0.028	7.2	0.009	0.089	8.5	0.005	0.103	3.0	0.085	0.040
Somatomotor B: S2 2	11.3	0.000	0.379	15.9	0.000	0.177	9.7	0.003	0.116	8.6	0.004	0.105	6.4	0.013	0.080
Somatomotor B: Central 1	15.3	0.000	0.453	13.3	0.000	0.152	9.4	0.003	0.113	6.8	0.011	0.084	2.0	0.160	0.026
Dorsal Attention A: Temporal Occipital 1	11.7	0.000	0.388	1.7	0.199	0.022	22.0	0.000	0.230	11.4	0.001	0.133	5.3	0.024	0.067
Dorsal Attention A: Parietal Occipital 1	10.1	0.000	0.353	0.2	0.646	0.003	17.8	0.000	0.194	9.1	0.003	0.110	3.9	0.052	0.050
Dorsal Attention A: Superior Parietal Lobule 1	11.8	0.000	0.389	14.1	0.000	0.160	6.0	0.016	0.076	9.6	0.003	0.115	2.5	0.117	0.033
Dorsal Attention B: Post Central 1	13.1	0.000	0.414	10.0	0.002	0.119	9.7	0.003	0.116	8.5	0.005	0.103	4.8	0.032	0.061
Dorsal Attention B: Post Central 2	9.6	0.000	0.342	1.1	0.290	0.015	17.6	0.000	0.192	8.2	0.005	0.100	4.9	0.031	0.062
Dorsal Attention B: Frontal Eye Fields 1	12.3	0.000	0.400	5.7	0.020	0.071	18.5	0.000	0.200	7.0	0.010	0.086	5.0	0.028	0.064
Salience Ventral Attention A: Parietal Operculum 1	12.7	0.000	0.408	0.9	0.350	0.012	14.9	0.000	0.167	8.4	0.005	0.102	1.3	0.264	0.017
Salience Ventral Attention A: Insula: 1	7.7	0.000	0.295	0.1	0.779	0.001	16.0	0.000	0.178	8.4	0.005	0.101	2.7	0.105	0.035
Salience Ventral Attention A: Parietal Medial 1	8.2	0.000	0.308	3.4	0.071	0.043	15.1	0.000	0.169	7.9	0.006	0.096	3.5	0.064	0.046
Salience Ventral Attention A: Frontal Medial 1	7.2	0.000	0.280	5.9	0.018	0.073	8.7	0.004	0.105	5.0	0.028	0.063	4.7	0.034	0.060
Salience Ventral Attention B: Inferior Parietal Lobule 1	9.4	0.000	0.336	0.0	0.997	0.000	19.3	0.000	0.207	8.7	0.004	0.105	3.9	0.051	0.051
Salience Ventral Attention B: Lateral Prefrontal Cortex	10.1	0.000	0.353	3.0	0.089	0.039	15.3	0.000	0.171	8.2	0.005	0.100	4.5	0.038	0.057
Salience Ventral Attention B: Medial Posterior Prefront	7.8	0.000	0.298	0.1	0.728	0.002	16.2	0.000	0.180	9.9	0.002	0.118	3.4	0.069	0.044
Limbic A: Temporal Pole 1	11.0	0.000	0.372	6.3	0.014	0.078	14.1	0.000	0.161	10.7	0.002	0.126	4.4	0.039	0.056
Limbic B: Orbital Frontal Cortex 1	13.0	0.000	0.413	9.0	0.004	0.108	13.1	0.001	0.151	9.5	0.003	0.114	4.8	0.031	0.061
Control A: Intraparietal Sulcus 1	11.3	0.000	0.379	5.1	0.028	0.064	18.6	0.000	0.201	10.4	0.002	0.123	5.5	0.022	0.069
Control A: Lateral Prefrontal Cortex 1	7.8	0.000	0.297	1.8	0.186	0.023	11.0	0.001	0.129	10.7	0.002	0.126	3.0	0.086	0.039
Control A: Lateral Prefrontal Cortex 2	7.5	0.000	0.288	2.1	0.150	0.028	12.4	0.001	0.143	12.2	0.001	0.141	5.5	0.021	0.069
Control B: Temporal 1	10.3	0.000	0.357	9.2	0.003	0.111	7.2	0.009	0.089	10.3	0.002	0.122	5.2	0.026	0.066
Control B: inferior parietal lobule 1	13.7	0.000	0.426	6.6	0.012	0.082	18.8	0.000	0.203	5.5	0.021	0.070	4.5	0.038	0.057
Control B: Lateral Prefrontal Cortexd 1	6.5	0.000	0.259	0.6	0.436	0.008	12.3	0.001	0.143	11.0	0.001	0.129	4.8	0.032	0.061
Control B: Lateral Prefrontal Cortexv 1	13.3	0.000	0.419	1.1	0.289	0.015	20.2	0.000	0.214	10.1	0.002	0.120	2.7	0.107	0.035
Control C: Cingulate Posterior 1	15.9	0.000	0.462	7.0	0.010	0.087	16.7	0.000	0.184	9.5	0.003	0.114	4.1	0.046	0.053
Control C: Precuneus 1	10.6	0.000	0.365	1.5	0.218	0.020	16.5	0.000	0.183	11.3	0.001	0.133	3.2	0.076	0.042
Default A: Inferior Parietal Lobule 1	11.1	0.000	0.374	1.8	0.180	0.024	15.3	0.000	0.171	8.9	0.004	0.108	2.7	0.103	0.036
Default A: Dorsal Prefrontal Cortex 1	6.5	0.000	0.260	4.0	0.049	0.051	9.3	0.003	0.112	8.7	0.004	0.105	3.7	0.059	0.047
Default A: Precuneus Posterior Cingulate Cortex 1	7.5	0.000	0.288	5.3	0.024	0.067	13.8	0.000	0.157	8.2	0.005	0.100	6.8	0.011	0.084
Default A: Medial Prefrontal Cortex 1	11.2	0.000	0.378	6.0	0.016	0.075	17.6	0.000	0.192	6.9	0.010	0.086	6.4	0.013	0.080
Default B: Dorsal Prefrontal Cortex 1	11.6	0.000	0.385	5.9	0.018	0.073	13.4	0.000	0.153	12.8	0.001	0.147	6.8	0.011	0.084
Default B: Ventral Prefrontal Cortex 1	7.3	0.000	0.284	3.2	0.077	0.042	11.0	0.001	0.129	7.5	0.008	0.092	5.1	0.026	0.065
Default B: Ventral Prefrontal Cortex 2	4.1	0.005	0.182	3.5	0.067	0.045	5.0	0.029	0.063	7.7	0.007	0.095	3.9	0.051	0.051
Default C: RetroSuperior Parietal Lobuleenial 1	2.8	0.032	0.131	0.0	0.896	0.000	4.4	0.040	0.056	7.6	0.007	0.093	2.3	0.133	0.030
Default C: Parahippocampal Cortex 1	5.1	0.001	0.215	1.0	0.321	0.013	8.3	0.005	0.100	8.8	0.004	0.106	6.1	0.016	0.076
Temporal Parietal 1	3.2	0.020	0.146	0.4	0.546	0.005	8.0	0.006	0.098	4.6	0.035	0.058	3.3	0.072	0.043
Temporal Parietal 2	6.9	0.000	0.271	4.1	0.047	0.052	8.8	0.004	0.106	7.3	0.009	0.089	4.2	0.044	0.054
Temporal Parietal 3	6.2	0.000	0.252	2.9	0.093	0.038	11.4	0.001	0.134	8.1	0.006	0.098	6.9	0.011	0.085
Caudate	4.7	0.002	0.202	2.1	0.149	0.028	5.1	0.027	0.064	6.5	0.013	0.080	2.5	0.115	0.033
Putamen	5.5	0.001	0.228	1.7	0.192	0.023	6.8	0.011	0.084	5.0	0.028	0.064	2.0	0.158	0.027
Pallidum	5.7	0.001	0.237	1.2	0.281	0.016	6.5	0.013	0.081	5.4	0.023	0.068	1.1	0.294	0.015
Thalamus	5.4	0.001	0.227	1.3	0.264	0.017	4.0	0.051	0.051	10.0	0.002	0.119	0.7	0.407	0.009

Supplementary Table 12. General Linear Models predicting each of the 5 principal components of cognition from network CMR_{GLC} 17 networks, age group, HOMA-IR, blood pressure, cortical thickness, as well as other demographics.

	Principal Component 1			Principal Component 2			Principal Component 3			Principal Component 4			Principal Component 5		
	F	p	η^2_p	F	p	η^2_p	F	p	η^2_p	F	p	η^2_p	F	p	η^2_p
Visual Central	2.7	0.110	0.069	0.3	0.608	0.007	5.3	0.027	0.128	0.5	0.500	0.013	0.3	0.560	0.010
Visual Peripheral	0.1	0.823	0.001	0.1	0.739	0.003	4.8	0.034	0.119	0.2	0.700	0.004	0.4	0.557	0.010
Somatomotor A	0.3	0.571	0.009	0.3	0.619	0.007	0.1	0.821	0.001	0.0	0.830	0.001	0.2	0.664	0.005
Somatomotor B	0.0	0.836	0.001	0.5	0.483	0.014	1.1	0.308	0.029	0.0	0.858	0.001	0.2	0.663	0.005
Dors Attention A	0.2	0.686	0.005	0.5	0.479	0.014	4.5	0.041	0.111	0.4	0.532	0.011	0.4	0.541	0.010
Dors Attention B	0.0	0.855	0.001	0.4	0.548	0.010	0.2	0.638	0.006	1.2	0.276	0.033	0.2	0.687	0.005
Salience Ventral Attention A	3.0	0.090	0.078	0.7	0.423	0.018	0.0	0.871	0.001	2.0	0.164	0.053	0.1	0.746	0.003
Salience Ventral Attention B	2.2	0.143	0.059	0.1	0.818	0.001	0.1	0.816	0.002	0.5	0.495	0.013	0.1	0.800	0.002
Limbic A	3.3	0.078	0.084	0.1	0.769	0.002	0.7	0.422	0.018	0.2	0.687	0.005	1.3	0.267	0.034
Limbic B	0.0	0.875	0.001	0.5	0.474	0.014	1.6	0.210	0.043	0.2	0.683	0.005	0.5	0.500	0.013
Control A	0.5	0.504	0.013	0.7	0.403	0.019	3.5	0.069	0.089	0.3	0.602	0.008	1.1	0.298	0.030
Control B	1.2	0.286	0.032	0.4	0.527	0.011	1.2	0.284	0.032	0.2	0.678	0.005	0.0	0.895	0.000
Control C	1.8	0.183	0.049	0.8	0.382	0.021	1.0	0.316	0.028	1.7	0.196	0.046	0.0	0.964	0.000
Default A	0.1	0.804	0.002	0.1	0.790	0.002	1.8	0.184	0.048	0.0	0.887	0.001	0.4	0.510	0.012
Default B	0.0	0.929	0.000	3.0	0.093	0.076	1.5	0.234	0.039	0.2	0.632	0.006	0.6	0.454	0.016
Default C	1.2	0.285	0.032	0.1	0.710	0.004	1.4	0.238	0.038	0.5	0.474	0.014	0.5	0.469	0.015
Temporal Parietal	2.1	0.158	0.054	0.1	0.759	0.003	0.1	0.794	0.002	3.5	0.069	0.089	0.4	0.536	0.011
Subcortical	1.7	0.200	0.045	1.0	0.315	0.028	0.2	0.700	0.004	1.7	0.199	0.045	0.2	0.644	0.006
Age Category	1.8	0.191	0.047	1.2	0.281	0.032	0.2	0.697	0.004	0.2	0.696	0.004	0.4	0.536	0.011
HOMA-IR	0.3	0.572	0.009	0.5	0.497	0.013	1.6	0.220	0.041	7.4	0.010	0.170	0.0	0.897	0.000
Systolic Blood Pressure	0.5	0.494	0.013	1.1	0.301	0.030	0.1	0.769	0.002	0.3	0.579	0.009	0.2	0.680	0.005
Diastolic Blood Pressure	1.3	0.259	0.035	3.4	0.072	0.087	0.7	0.418	0.018	2.8	0.106	0.071	0.5	0.467	0.015
Cortical Thickness	4.9	0.033	0.121	1.0	0.326	0.027	0.9	0.337	0.026	0.4	0.528	0.011	0.8	0.374	0.022
Eduction Years	0.0	0.900	0.000	0.6	0.448	0.016	0.9	0.352	0.024	1.6	0.209	0.043	0.3	0.605	0.008
Sex	1.7	0.202	0.045	0.0	0.992	0.000	0.0	0.859	0.001	0.3	0.577	0.009	0.4	0.515	0.012
Number Medications	2.6	0.118	0.066	1.1	0.305	0.029	0.0	0.937	0.000	1.1	0.310	0.029	0.1	0.718	0.004
Country of Birth	6.2	0.017	0.148	0.2	0.637	0.006	0.3	0.574	0.009	0.4	0.542	0.010	0.2	0.631	0.006

7. Association of Fasting Blood Glucose with regional CMR_{GLU}, and Cognition

We examined the association of fasting blood glucose on regional CMR_{GLC} by repeating the GLM models from the main analyses with the blood glucose replacing HOMA-IR in the analyses. The overall GLMs were significant for 96 of 108 regions (Supplementary Table 13). Interestingly, the effect of age group on regional CMR_{GLC} was not significant in any region. However, the age group x fasting blood glucose interaction was significant in 31 regions (vs 41 for HOMA-IR and age group interactions; Supplementary Table 6). The post-hoc univariate analyses revealed that a higher fasting blood glucose was associated with lower CRM_{GLC} in 12 regions for older but not younger adults (Supplementary Table 14). This pattern was seen in regions across the somatomotor, dorsal attention and salience ventral attention, default, control networks and subcortex (see Supplementary Figure 3 for plots of example regions). Whereas higher HOMA-IR was associated with lower CMR_{GLC} for younger but not older adults, higher fasting blood glucose was associated with lower CMR_{GLC} for older adults only. These results suggest that fasting blood glucose and insulin resistance provide complementary information about CMR_{GLC} changes in normative ageing.

The GLMs predicting the 5 cognitive principal components from the main manuscript were repeated with fasting blood glucose replacing HOMA-IR (see Supplementary Table 15) and the other demographic variables. However, fasting blood glucose was not significantly associated with cognition when network CMR_{GLC}, age, blood pressure cortical thickness and the other demographics were also in the GLMs.

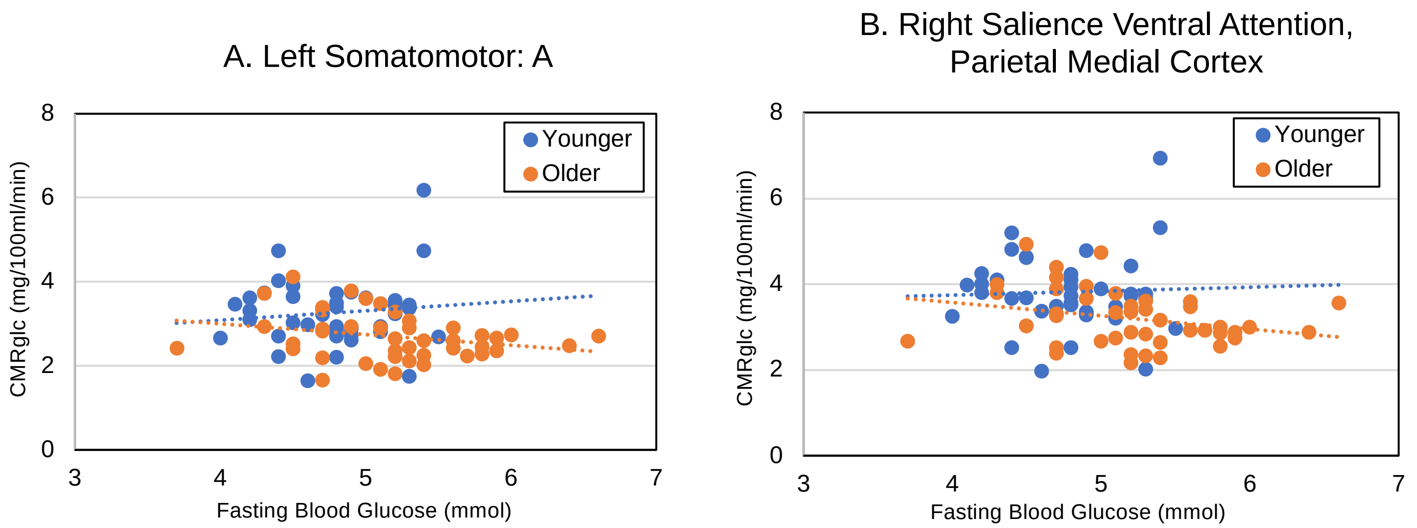

Supplementary Figure 3. Example age group X fasting blood glucose associations with regional CMR_{GLC} for (A) Left somatomotor and (B) right salience ventral attention parietal medial cortex.

Supplementary Table 13. General linear models of age group, fasting blood glucose and age group x fasting blood glucose effect on regional CMR_{GLC}, including cortical thickness in the same region as a covariate. For the significant interactions, post-hoc univariate models for younger and older adults are reported in Supplementary Table 14.

	Overall Model			Cortical Thickness			Age Group			Blood Glucose			Age Group x Blood Glucose		
	F	p-FDR	Eta2	F	p	Eta2	F	p	Eta2	F	p	Eta2	F	p	Eta2
Visual Central: Extra Striate Cortex 1	1.0	0.405	5%	0.5	0.474	1%	0.4	0.555	0%	0.1	0.723	0%	0.8	0.384	1%
Visual Central: Extra Striate Cortex 2	0.5	0.734	3%	0.1	0.701	0%	0.1	0.767	0%	0.1	0.793	0%	0.2	0.676	0%
Visual Central: Striate Cortex 1	1.2	0.325	6%	0.5	0.469	1%	0.3	0.570	0%	0.0	0.871	0%	0.2	0.668	0%
Visual Central: Extra Striate Cortex 3	1.9	0.138	9%	0.2	0.676	0%	0.1	0.795	0%	0.0	0.905	0%	1.6	0.213	2%
Visual Peripheral: Extra Striate Inferior 1	2.6	0.050	12%	0.4	0.513	1%	0.2	0.676	0%	0.0	0.981	0%	0.5	0.477	1%
Visual Peripheral: Striate Cortex Calcarine 1	3.6	0.012	16%	0.7	0.419	1%	0.4	0.520	1%	0.0	0.977	0%	5.7	0.020	7%
Visual Peripheral: Extra Striate CortexSup 1	3.3	0.018	15%	0.4	0.530	1%	0.1	0.702	0%	0.0	0.902	0%	1.5	0.224	2%
Somatomotor A: 1	6.8	0.000	27%	1.6	0.214	2%	0.9	0.343	1%	0.2	0.678	0%	6.1	0.016	8%
Somatomotor A: 2	5.9	0.001	24%	2.1	0.148	3%	1.6	0.206	2%	0.0	0.844	0%	5.1	0.027	6%
Somatomotor B: Auditory 1	4.7	0.003	20%	0.5	0.470	1%	0.3	0.600	0%	0.0	0.948	0%	3.9	0.051	5%
Somatomotor B: S2 1	4.3	0.005	19%	1.8	0.184	2%	1.0	0.311	1%	0.2	0.692	0%	0.2	0.648	0%
Somatomotor B: S2 2	8.2	0.000	31%	0.1	0.791	0%	0.0	0.904	0%	0.0	0.835	0%	2.2	0.141	3%
Somatomotor B: Central 1	3.5	0.014	16%	0.5	0.503	1%	0.2	0.671	0%	0.0	0.972	0%	2.0	0.161	3%
Dorsal Attention A: Temporal Occipital 1	5.3	0.002	22%	0.1	0.788	0%	0.0	0.944	0%	0.4	0.517	1%	10.0	0.002	12%
Dorsal Attention A: Parietal Occipital 1	3.2	0.021	15%	1.7	0.195	2%	1.2	0.273	2%	0.2	0.659	0%	2.5	0.117	3%
Dorsal Attention A: Superior Parietal Lobule 1	4.7	0.003	20%	1.0	0.325	1%	0.6	0.436	1%	0.3	0.580	0%	5.2	0.025	7%
Dorsal Attention B: Post Central 1	5.1	0.002	22%	1.0	0.330	1%	0.4	0.552	0%	0.1	0.794	0%	0.1	0.712	0%
Dorsal Attention B: Post Central 2	9.2	0.000	33%	0.7	0.398	1%	0.4	0.510	1%	0.6	0.444	1%	15.4	0.000	17%
Dorsal Attention B: Post Central 3	7.1	0.000	28%	1.2	0.268	2%	0.7	0.392	1%	0.1	0.714	0%	8.8	0.004	11%
Dorsal Attention B: Frontal Eye Fields 1	8.9	0.000	33%	3.1	0.082	4%	1.9	0.168	3%	0.2	0.664	0%	2.7	0.104	4%
Salience Ventral Attention A: Parietal Operculum 1	5.8	0.001	24%	0.7	0.403	1%	0.2	0.629	0%	0.1	0.718	0%	1.3	0.265	2%
Salience Ventral Attention A: Insula: 1	8.4	0.000	31%	0.2	0.649	0%	0.0	0.994	0%	0.0	0.869	0%	0.9	0.336	1%
Salience Ventral Attention A: Insula: 2	8.8	0.000	32%	0.3	0.606	0%	0.0	0.839	0%	0.1	0.804	0%	4.5	0.038	6%
Salience Ventral Attention A: Parietal Medial 1	8.0	0.000	30%	0.6	0.450	1%	0.4	0.529	1%	1.0	0.327	1%	10.4	0.002	12%
Salience Ventral Attention A: Frontal Medial 1	8.7	0.000	32%	2.1	0.156	3%	1.4	0.237	2%	0.0	0.827	0%	4.8	0.032	6%
Salience Ventral Attention B: Lateral Prefrontal Cortex 1	11.5	0.000	38%	0.5	0.480	1%	0.2	0.685	0%	0.2	0.625	0%	9.9	0.002	12%
Salience Ventral Attention B: Medial Posterior Prefrontal 1	8.7	0.000	32%	1.9	0.177	2%	1.0	0.324	1%	0.4	0.553	0%	0.7	0.394	1%
Limbic A Temporal Pole 1	5.2	0.002	22%	1.3	0.262	2%	0.6	0.447	1%	0.1	0.791	0%	0.0	0.843	0%
Limbic A: Temporal Pole 2	4.8	0.003	21%	0.8	0.372	1%	0.3	0.566	0%	0.0	0.963	0%	2.3	0.132	3%
Limbic B: Orbital Frontal Cortex 1	3.2	0.021	15%	0.7	0.399	1%	0.3	0.591	0%	0.0	0.952	0%	0.0	0.970	0%
Control A: Intraparietal Sulcus 1	7.3	0.000	28%	2.1	0.150	3%	1.3	0.252	2%	0.8	0.388	1%	4.5	0.037	6%
Control A: Lateral Prefrontal Cortex 1	7.0	0.000	27%	1.0	0.325	1%	0.5	0.499	1%	0.0	0.882	0%	2.7	0.104	4%
Control A: Lateral Prefrontal Cortex 2	8.7	0.000	32%	1.5	0.228	2%	1.0	0.310	1%	0.1	0.774	0%	8.5	0.005	10%
Control B: Lateral Prefrontal Cortexv 1	6.7	0.000	27%	0.6	0.424	1%	0.2	0.634	0%	0.0	0.999	0%	5.0	0.029	6%
Control C: Precuneus 1	3.0	0.029	14%	0.7	0.404	1%	0.4	0.511	1%	0.4	0.529	1%	1.0	0.309	1%
Control C: Precuneus 2	4.0	0.008	18%	1.1	0.305	1%	0.7	0.397	1%	0.3	0.564	0%	2.0	0.161	3%
Control C: Cingulate Posterior 1	3.1	0.025	14%	1.1	0.304	1%	0.7	0.418	1%	0.0	0.883	0%	0.8	0.383	1%
Default A: Dorsal Prefrontal Cortex 1	13.2	0.000	42%	2.1	0.148	3%	1.2	0.281	2%	0.0	0.917	0%	9.8	0.002	12%
Default A: Precuneus Posterior Cingulate Cortex1	3.3	0.018	15%	1.3	0.262	2%	0.9	0.344	1%	0.1	0.716	0%	1.2	0.274	2%
Default A: Medial Prefrontal Cortex 1	9.1	0.000	33%	0.8	0.362	1%	0.2	0.646	0%	0.1	0.794	0%	1.0	0.316	1%
Default B: Temp 1	5.5	0.001	23%	0.3	0.612	0%	0.0	0.851	0%	0.0	0.980	0%	1.2	0.275	2%
Default B: Temp 2	6.1	0.001	25%	0.3	0.591	0%	0.1	0.768	0%	0.2	0.699	0%	5.1	0.027	6%
Default B: Inferior Parietal Lobule 1	4.9	0.003	21%	0.8	0.366	1%	0.5	0.484	1%	0.0	0.949	0%	2.4	0.126	3%
Default B: Dorsal Prefrontal Cortex 1	11.7	0.000	39%	1.9	0.176	2%	0.9	0.343	1%	0.0	0.831	0%	2.9	0.095	4%
Default B: Lateral Prefrontal Cortex 1	7.7	0.000	29%	0.7	0.410	1%	0.3	0.619	0%	0.1	0.791	0%	3.5	0.064	5%
Default B: Ventral Prefrontal Cortex 1	7.8	0.000	30%	0.8	0.381	1%	0.2	0.698	0%	0.2	0.673	0%	0.3	0.573	0%
Default B: Ventral Prefrontal Cortex 2	6.2	0.001	25%	0.9	0.335	1%	0.4	0.521	1%	0.0	0.981	0%	1.7	0.201	2%
Default C: RetroSuperior Parietal Lobuleenial 1	3.5	0.014	16%	0.7	0.422	1%	0.3	0.593	0%	0.2	0.698	0%	0.4	0.543	1%
Default C: Parahippocampal Cortex 1	4.9	0.003	21%	0.2	0.681	0%	0.0	0.902	0%	0.2	0.624	0%	2.7	0.106	3%
Temporal Parietal 1	5.1	0.002	22%	1.1	0.307	1%	0.6	0.454	1%	0.1	0.703	0%	2.8	0.100	4%
Caudate	3.6	0.013	16%	0.2	0.623	0%	0.1	0.754	0%	0.2	0.628	0%	1.8	0.181	2%
Putamen	4.5	0.004	20%	0.6	0.456	1%	0.2	0.629	0%	0.2	0.688	0%	0.9	0.347	1%
Pallidum	5.1	0.002	22%	2.9	0.093	4%	2.0	0.159	3%	0.0	0.846	0%	0.5	0.490	1%
Thalamus	3.7	0.011	17%	2.1	0.154	3%	1.6	0.214	2%	0.1	0.712	0%	0.9	0.347	1%

	Overall Model			Cortical Thickness			Age Group			Blood Glucose			Age Group x Blood Glucose		
	F	p-FDR	Eta2	F	p	Eta2	F	p	Eta2	F	p	Eta2	F	p	Eta2
Visual Central: Extra Striate Cortex 1	1.3	0.276	7%	0.5	0.498	1%	0.4	0.545	0%	0.0	0.833	0%	1.7	0.203	2%
Visual Central: Extra Striate Cortex 2	0.7	0.593	4%	0.4	0.546	0%	0.3	0.595	0%	0.4	0.515	1%	0.0	0.993	0%
Visual Central: Extra Striate Cortex 3	1.5	0.216	8%	0.7	0.408	1%	0.5	0.466	1%	0.0	0.831	0%	1.3	0.258	2%
Visual Peripheral: Striate Cortex Calcarine 1	1.4	0.268	7%	0.6	0.436	1%	0.4	0.546	0%	0.0	0.893	0%	0.0	0.901	0%
Visual Peripheral: Extra Striate Inferior 1	3.9	0.009	17%	0.6	0.454	1%	0.3	0.573	0%	0.1	0.773	0%	3.0	0.086	4%
Visual Peripheral: Extra Striate Superior 1	2.4	0.065	11%	0.6	0.444	1%	0.4	0.554	0%	0.0	0.991	0%	1.8	0.187	2%
Somatomotor A: 1	4.8	0.003	21%	0.9	0.349	1%	0.4	0.531	1%	0.0	0.890	0%	1.0	0.313	1%
Somatomotor A: 2	4.4	0.005	19%	1.2	0.277	2%	0.7	0.418	1%	0.0	0.839	0%	2.5	0.120	3%
Somatomotor A: 3	5.4	0.002	23%	1.4	0.237	2%	0.9	0.341	1%	0.1	0.783	0%	5.5	0.022	7%
Somatomotor A: 4	6.1	0.001	25%	3.2	0.080	4%	2.5	0.121	3%	0.0	0.934	0%	4.6	0.035	6%
Somatomotor B: Auditory 1	5.2	0.002	22%	0.4	0.547	0%	0.2	0.662	0%	0.0	0.938	0%	5.7	0.019	7%
Somatomotor B: S2 1	4.9	0.003	21%	0.9	0.357	1%	0.4	0.555	0%	0.1	0.815	0%	0.2	0.634	0%
Somatomotor B: S2 2	6.7	0.000	27%	0.1	0.749	0%	0.0	0.987	0%	0.4	0.506	1%	1.8	0.186	2%
Somatomotor B: Central 1	3.1	0.023	14%	1.1	0.298	1%	0.8	0.379	1%	0.0	0.977	0%	3.1	0.080	4%
Dorsal Attention A: Temporal Occipital 1	1.7	0.159	9%	0.3	0.572	0%	0.1	0.724	0%	0.0	0.860	0%	0.3	0.598	0%
Dorsal Attention A: Parietal Occipital 1	3.1	0.024	14%	0.4	0.546	0%	0.1	0.706	0%	0.0	0.858	0%	1.2	0.271	2%
Dorsal Attention A: Superior Parietal Lobule 1	4.3	0.005	19%	0.6	0.446	1%	0.3	0.597	0%	0.0	0.961	0%	3.3	0.075	4%
Dorsal Attention B: Post Central 1	5.0	0.002	21%	1.1	0.309	1%	0.7	0.405	1%	0.0	0.853	0%	3.4	0.069	4%
Dorsal Attention B: Post Central 2	6.6	0.000	26%	0.8	0.383	1%	0.5	0.462	1%	0.0	0.956	0%	11.4	0.001	13%
Dorsal Attention B: Frontal Eye Fields 1	13.5	0.000	42%	4.0	0.050	5%	2.9	0.091	4%	0.2	0.699	0%	14.6	0.000	17%
Salience Ventral Attention A: Parietal Operculum 1	6.6	0.000	26%	0.9	0.339	1%	0.3	0.564	0%	0.0	0.906	0%	0.9	0.336	1%
Salience Ventral Attention A: Insula: 1	6.1	0.001	25%	0.5	0.472	1%	0.1	0.744	0%	0.1	0.817	0%	0.1	0.726	0%
Salience Ventral Attention A: Parietal Medial 1	8.9	0.000	32%	2.7	0.103	4%	2.3	0.137	3%	0.0	0.883	0%	14.2	0.000	16%
Salience Ventral Attention A: Frontal Medial 1	9.0	0.000	33%	1.6	0.209	2%	1.1	0.294	1%	0.0	0.957	0%	5.9	0.018	7%
Salience Ventral Attention B: Inferior Parietal Lobule 1	5.6	0.001	23%	0.3	0.578	0%	0.0	0.825	0%	0.0	0.951	0%	1.6	0.209	2%
Salience Ventral Attention B: Lateral Prefrontal Cortex 1	8.6	0.000	32%	1.9	0.173	2%	1.0	0.311	1%	0.1	0.804	0%	4.6	0.035	6%
Salience Ventral Attention B: Medial Posterior Prefrontal 1	10.5	0.000	36%	2.0	0.160	3%	0.9	0.337	1%	0.2	0.665	0%	1.0	0.327	1%
Limbic A: Temporal Pole 1	4.9	0.003	21%	1.3	0.252	2%	0.6	0.433	1%	0.0	0.995	0%	0.0	0.911	0%
Limbic B: Orbital Frontal Cortex 1	5.0	0.003	21%	0.8	0.373	1%	0.3	0.571	0%	0.0	0.894	0%	2.5	0.118	3%
Control A: Intraparietal Sulcus 1	4.8	0.003	21%	1.7	0.195	2%	1.3	0.266	2%	0.6	0.436	1%	6.1	0.016	8%
Control A: Lateral Prefrontal Cortex 1	5.8	0.001	24%	1.3	0.267	2%	0.5	0.489	1%	0.0	0.886	0%	0.0	0.976	0%
Control A: Lateral Prefrontal Cortex 2	6.2	0.001	25%	0.6	0.448	1%	0.2	0.657	0%	0.0	0.919	0%	1.8	0.185	2%
Control B: Temporal 1	4.3	0.006	19%	0.5	0.501	1%	0.1	0.740	0%	0.2	0.642	0%	0.0	0.877	0%
Control B: inferior parietal lobule 1	6.6	0.000	26%	1.3	0.266	2%	0.7	0.395	1%	0.0	0.860	0%	5.6	0.021	7%
Control B: Lateral Prefrontal Cortexd 1	8.6	0.000	32%	1.4	0.247	2%	0.8	0.374	1%	0.0	0.941	0%	5.1	0.027	6%
Control B: Lateral Prefrontal Cortexv 1	6.7	0.000	27%	0.8	0.379	1%	0.3	0.556	0%	0.3	0.608	0%	4.8	0.032	6%
Control C: Cingulate Posterior 1	4.8	0.003	21%	1.8	0.187	2%	1.2	0.278	2%	0.3	0.594	0%	1.5	0.231	2%
Control C: Precuneus 1	3.2	0.021	15%	1.6	0.206	2%	1.2	0.280	2%	0.1	0.767	0%	1.4	0.248	2%
Default A: Inferior Parietal Lobule 1	6.0	0.001	24%	1.8	0.180	2%	1.5	0.228	2%	0.3	0.596	0%	7.2	0.009	9%
Default A: Dorsal Prefrontal Cortex 1	10.4	0.000	36%	1.2	0.271	2%	0.5	0.478	1%	0.1	0.766	0%	4.5	0.038	6%
Default A: Precuneus Posterior Cingulate Cortex 1	2.6	0.050	12%	1.4	0.243	2%	0.9	0.344	1%	0.0	0.960	0%	0.1	0.724	0%
Default A: Medial Prefrontal Cortex 1	9.4	0.000	34%	1.5	0.229	2%	0.6	0.450	1%	0.0	0.959	0%	1.6	0.214	2%
Default B: Dorsal Prefrontal Cortex 1	11.2	0.000	38%	1.4	0.238	2%	0.6	0.424	1%	0.1	0.759	0%	4.4	0.039	6%
Default B: Ventral Prefrontal Cortex 1	6.6	0.000	26%	1.2	0.268	2%	0.6	0.456	1%	0.1	0.722	0%	0.8	0.367	1%
Default B: Ventral Prefrontal Cortex 2	7.6	0.000	29%	0.7	0.398	1%	0.2	0.623	0%	0.2	0.690	0%	1.9	0.174	2%
Default C: RetroSuperior Parietal Lobuleenial 1	3.5	0.014	16%	0.3	0.592	0%	0.1	0.713	0%	0.8	0.380	1%	2.4	0.123	3%
Default C: Parahippocampal Cortex 1	3.3	0.018	15%	0.4	0.543	1%	0.2	0.691	0%	0.0	0.935	0%	3.0	0.086	4%
Temporal Parietal 1	6.8	0.000	27%	0.0	0.969	0%	0.1	0.710	0%	0.0	0.871	0%	3.2	0.077	4%
Temporal Parietal 2	5.8	0.001	24%	0.9	0.338	1%	0.5	0.494	1%	0.0	0.924	0%	2.1	0.148	3%
Temporal Parietal 3	3.9	0.009	17%	0.8	0.373	1%	0.4	0.509	1%	0.1	0.801	0%	1.9	0.172	3%
Caudate	2.6	0.050	12%	0.6	0.440	1%	0.4	0.526	1%	0.1	0.712	0%	1.3	0.260	2%
Putamen	3.8	0.010	17%	0.6	0.459	1%	0.3	0.602	0%	0.2	0.696	0%	1.1	0.306	1%
Pallidum	4.6	0.003	20%	1.7	0.196	2%	1.1	0.299	1%	0.1	0.758	0%	0.7	0.408	1%
Thalamus	3.6	0.014	16%	2.6	0.109	3%	2.1	0.153	3%	0.1	0.745	0%	0.8	0.388	1%

Supplementary Table 14. Post hoc analyses of significant age group x fasting blood glucose interactions (from Supplementary Table 13); separate general linear models of CMR_{GLC} for younger and older adults. The models include cortical thickness as a covariate.

	Age Group x Blood Glucose			Post-Hoc Younger		Post-Hoc Older			Age Group x Blood Glucose			Post-Hoc Younger		Post-Hoc Older	
	F	p	Eta2	F	p	F	P		F	p	Eta2	F	p	F	P
Visual Central: Extra Striate Cortex 1	0.8	0.384	1%					Visual Central: Extra Striate Cortex 1	1.7	0.203	2%				
Visual Central: Extra Striate Cortex 2	0.2	0.676	0%					Visual Central: Extra Striate Cortex 2	0.0	0.993	0%				
Visual Central: Striate Cortex 1	0.2	0.668	0%					Visual Central: Extra Striate Cortex 3	1.3	0.258	2%				
Visual Central: Extra Striate Cortex 3	1.6	0.213	2%					Visual Peripheral: Striate Cortex Calcarine 1	0.0	0.901	0%				
Visual Peripheral: Extra Striate Inferior 1	0.5	0.477	1%					Visual Peripheral: Extra Striate Inferior 1	3.0	0.086	4%				
Visual Peripheral: Striate Cortex Calcarine 1	5.7	0.020	7%	0.10	0.748	0.98	0.3279	Visual Peripheral: Extra Striate Superior 1	1.8	0.187	2%				
Visual Peripheral: Extra Striate CortexSup 1	1.5	0.224	2%												
Somatomotor A: 1	6.1	0.016	8%					Somatomotor A: 1	1.0	0.313	1%				
Somatomotor A: 2	5.1	0.027	6%	0.43	0.518	4.60	0.0382	Somatomotor A: 2	2.5	0.120	3%				
Somatomotor B: Auditory 1	3.9	0.051	5%					Somatomotor A: 3	5.5	0.022	7%	0.44	0.513	0.59	0.4464
Somatomotor B: S2 1	0.2	0.648	0%					Somatomotor A: 4	4.6	0.035	6%	0.68	0.415	5.80	0.0207
Somatomotor B: S2 2	2.2	0.141	3%					Somatomotor B: Auditory 1	5.7	0.019	7%	0.11	0.739	0.58	0.4501
Somatomotor B: Central 1	2.0	0.161	3%					Somatomotor B: S2 1	0.2	0.634	0%				
								Somatomotor B: S2 2	1.8	0.186	2%				
								Somatomotor B: Central 1	3.1	0.080	4%				
Dorsal Attention A: Temporal Occipital 1	10.0	0.002	12%	0.17	0.680	0.27	0.6031	Dorsal Attention A: Temporal Occipital 1	0.3	0.598	0%				
Dorsal Attention A: Parietal Occipital 1	2.5	0.117	3%					Dorsal Attention A: Parietal Occipital 1	1.2	0.271	2%				
Dorsal Attention A: Superior Parietal Lobule 1	5.2	0.025	7%	0.29	0.594	0.20	0.6576	Dorsal Attention A: Superior Parietal Lobule 1	3.3	0.075	4%				
Dorsal Attention B: Post Central 1	0.1	0.712	0%					Dorsal Attention B: Post Central 1	3.4	0.069	4%				
Dorsal Attention B: Post Central 2	15.4	0.000	17%	0.58	0.452	0.07	0.7998	Dorsal Attention B: Post Central 2	11.4	0.001	13%	0.21	0.648	0.78	0.3838
Dorsal Attention B: Post Central 3	8.8	0.004	11%	0.42	0.521	1.22	0.2760	Dorsal Attention B: Frontal Eye Fields 1	14.6	0.000	17%	1.07	0.310	4.78	0.0347
Dorsal Attention B: Frontal Eye Fields 1	2.7	0.104	4%												
Salience Ventral Attention A: Parietal Operculum 1	1.3	0.265	2%					Salience Ventral Attention A: Parietal Operculum 1	0.9	0.336	1%				
Salience Ventral Attention A: Insula: 1	0.9	0.336	1%					Salience Ventral Attention A: Insula: 1	0.1	0.726	0%				
Salience Ventral Attention A: Insula: 2	4.5	0.038	6%	0.00	0.991	1.17	0.2866	Salience Ventral Attention A: Parietal Medial 1	14.2	0.000	16%	0.69	0.414	4.78	0.0346
Salience Ventral Attention A: Parietal Medial 1	10.4	0.002	12%	0.01	0.926	4.68	0.0365	Salience Ventral Attention A: Frontal Medial 1	5.9	0.018	7%	0.40	0.533	2.28	0.1387
Salience Ventral Attention A: Frontal Medial 1	4.8	0.032	6%	0.53	0.470	1.11	0.2975	Salience Ventral Attention B: Inferior Parietal Lobule 1	1.6	0.209	2%				
Salience Ventral Attention B: Lateral Prefrontal Cortex 1	9.9	0.002	12%	0.04	0.847	3.50	0.0689	Salience Ventral Attention B: Lateral Prefrontal Cortex 1	4.6	0.035	6%	0.48	0.495	1.87	0.1791
Salience Ventral Attention B: Medial Posterior Prefrontal 1	0.7	0.394	1%					Salience Ventral Attention B: Medial Posterior Prefrontal 1	1.0	0.327	1%				
Limbic A Temporal Pole 1	0.0	0.843	0%					Limbic A: Temporal Pole 1	0.0	0.911	0%				
Limbic A: Temporal Pole 2	2.3	0.132	3%					Limbic B: Orbital Frontal Cortex 1	2.5	0.118	3%				
Limbic B: Orbital Frontal Cortex 1	0.0	0.970	0%												
Control A: Intraparietal Sulcus 1	4.5	0.037	6%	1.12	0.297	0.36	0.5511	Control A: Intraparietal Sulcus 1	6.1	0.016	8%	0.86	0.361	0.31	0.5810
Control A: Lateral Prefrontal Cortex 1	2.7	0.104	4%					Control A: Lateral Prefrontal Cortex 1	0.0	0.976	0%				
Control A: Lateral Prefrontal Cortex 2	8.5	0.005	10%	0.47	0.496	1.49	0.2296	Control A: Lateral Prefrontal Cortex 2	1.8	0.185	2%				
Control B: Lateral Prefrontal Cortexv 1	5.0	0.029	6%	0.02	0.884	1.14	0.2913	Control B: Temporal 1	0.0	0.877	0%				
Control C: Precuneus 1	1.0	0.309	1%					Control B: inferior parietal lobule 1	5.6	0.021	7%	0.13	0.719	2.07	0.1579
Control C: Precuneus 2	2.0	0.161	3%					Control B: Lateral Prefrontal Cortexd 1	5.1	0.027	6%	0.41	0.529	1.62	0.2099
Control C: Cingulate Posterior 1	0.8	0.383	1%					Control B: Lateral Prefrontal Cortexv 1	4.8	0.032	6%	0.00	0.944	3.26	0.0784
								Control C: Cingulate Posterior 1	1.5	0.231	2%				
								Control C: Precuneus 1	1.4	0.248	2%				
Default A: Dorsal Prefrontal Cortex 1	9.8	0.002	12%	0.52	0.477	3.51	0.0681	Default A: Inferior Parietal Lobule 1	7.2	0.009	9%	0.45	0.508	0.44	0.5133
Default A: Precuneus Posterior Cingulate Cortex1	1.2	0.274	2%					Default A: Dorsal Prefrontal Cortex 1	4.5	0.038	6%	0.38	0.541	1.58	0.2158
Default A: Medial Prefrontal Cortex 1	1.0	0.316	1%					Default A: Precuneus Posterior Cingulate Cortex 1	0.1	0.724	0%				
Default B: Temp 1	1.2	0.275	2%					Default A: Medial Prefrontal Cortex 1	1.6	0.214	2%				
Default B: Temp 2	5.1	0.027	6%	0.20	0.655	0.00	0.9489	Default B: Dorsal Prefrontal Cortex 1	4.4	0.039	6%	0.47	0.498	0.68	0.4147
Default B: Inferior Parietal Lobule 1	2.4	0.126	3%					Default B: Ventral Prefrontal Cortex 1	0.8	0.367	1%				
Default B: Dorsal Prefrontal Cortex 1	2.9	0.095	4%					Default B: Ventral Prefrontal Cortex 2	1.9	0.174	2%				
Default B: Lateral Prefrontal Cortex 1	3.5	0.064	5%					Default C: RetroSuperior Parietal Lobuleenial 1	2.4	0.123	3%				
Default B: Ventral Prefrontal Cortex 1	0.3	0.573	0%					Default C: Parahippocampal Cortex 1	3.0	0.086	4%				
Default B: Ventral Prefrontal Cortex 2	1.7	0.201	2%												
Default C: RetroSuperior Parietal Lobuleenial 1	0.4	0.543	1%												
Default C: Parahippocampal Cortex 1	2.7	0.106	3%												
Temporal Parietal 1	2.8	0.100	4%					Temporal Parietal 1	3.2	0.077	4%				
								Temporal Parietal 2	2.1	0.148	3%				
								Temporal Parietal 3	1.9	0.172	3%				
Caudate	1.8	0.181	2%					Caudate	1.3	0.260	2%				
Putamen	0.9	0.347	1%					Putamen	1.1	0.306	1%				
Pallidum	0.5	0.490	1%					Pallidum	0.7	0.408	1%				
Thalamus	0.9	0.347	1%					Thalamus	0.8	0.388	1%				

Supplementary Table 15. General Linear Models predicting each of the 5 principal components of cognition from network CMR_{GLC} 17 networks, age group, fasting blood glucose and other demographic variables.

	Principal Component 1			Principal Component 2			Principal Component 3			Principal Component 4			Principal Component 5		
	F	p	η^2_p	F	p	η^2_p	F	p	η^2_p	F	p	η^2_p	F	p	η^2_p
Visual Central	2.8	0.101	0.073	0.8	0.370	0.022	4.2	0.048	0.104	1.0	0.317	0.028	0.1	0.805	0.002
Visual Peripheral	0.1	0.819	0.001	0.1	0.789	0.002	3.3	0.078	0.084	0.1	0.705	0.004	1.5	0.224	0.041
Somatomotor A	0.3	0.577	0.009	0.1	0.718	0.004	0.1	0.761	0.003	0.0	0.947	0.000	0.4	0.545	0.010
Somatomotor B	0.1	0.808	0.002	0.3	0.598	0.008	0.7	0.404	0.019	0.2	0.622	0.007	0.4	0.538	0.011
Dors Attention A	0.2	0.696	0.004	0.5	0.487	0.013	4.5	0.041	0.111	0.3	0.615	0.007	0.4	0.513	0.012
Dors Attention B	0.1	0.812	0.002	0.2	0.635	0.006	0.2	0.661	0.005	1.0	0.318	0.028	0.1	0.805	0.002
Salience Ventral Attention A	3.3	0.079	0.083	1.1	0.309	0.029	0.0	0.826	0.001	1.6	0.221	0.041	0.4	0.552	0.010
Salience Ventral Attention B	2.1	0.153	0.056	0.2	0.672	0.005	0.2	0.656	0.006	0.1	0.820	0.001	0.0	0.877	0.001
Limbic A	2.5	0.122	0.065	0.3	0.591	0.008	0.7	0.392	0.020	0.0	0.942	0.000	2.5	0.124	0.065
Limbic B	0.0	0.966	0.000	0.2	0.652	0.006	1.4	0.240	0.038	0.1	0.722	0.004	0.1	0.729	0.003
Control A	0.4	0.542	0.010	0.2	0.621	0.007	4.0	0.053	0.100	0.1	0.808	0.002	2.3	0.142	0.059
Control B	1.2	0.285	0.032	0.8	0.391	0.020	0.8	0.369	0.022	0.0	0.920	0.000	0.1	0.743	0.003
Control C	2.2	0.145	0.058	1.6	0.214	0.043	0.5	0.486	0.014	0.4	0.525	0.011	0.0	0.846	0.001
Default A	0.0	0.840	0.001	0.0	0.910	0.000	1.6	0.210	0.043	0.0	0.934	0.000	0.3	0.615	0.007
Default B	0.0	0.958	0.000	3.0	0.094	0.076	1.6	0.215	0.042	0.1	0.772	0.002	0.6	0.430	0.017
Default C	1.6	0.215	0.042	0.1	0.711	0.004	1.9	0.173	0.051	0.0	0.840	0.001	0.4	0.540	0.011
Temporal Parietal	2.3	0.137	0.060	0.0	0.954	0.000	0.2	0.694	0.004	2.4	0.134	0.061	1.2	0.288	0.031
Subcortical	2.0	0.166	0.053	0.6	0.446	0.016	0.0	0.919	0.000	0.5	0.488	0.013	0.1	0.775	0.002
Age Category	2.3	0.139	0.060	0.7	0.403	0.020	0.2	0.642	0.006	0.0	0.959	0.000	1.1	0.298	0.030
Fasting Blood Glucose (mmolL)	0.7	0.402	0.020	1.7	0.205	0.044	0.0	0.836	0.001	0.9	0.338	0.026	3.9	0.055	0.099
Systolic Blood Pressure	0.6	0.431	0.017	1.4	0.248	0.037	0.1	0.820	0.001	0.1	0.737	0.003	0.4	0.528	0.011
Diastolic Blood Pressure	1.3	0.256	0.036	3.1	0.085	0.080	0.5	0.472	0.014	2.0	0.169	0.052	0.4	0.509	0.012
Cortical Thickness	5.1	0.029	0.125	1.2	0.276	0.033	0.9	0.339	0.025	0.3	0.559	0.010	1.1	0.291	0.031
Eduction Years	0.0	0.841	0.001	0.5	0.493	0.013	0.8	0.383	0.021	1.7	0.203	0.045	0.5	0.491	0.013
Sex	1.9	0.174	0.051	0.2	0.665	0.005	0.1	0.718	0.004	0.6	0.443	0.016	0.0	0.856	0.001
Number Medications	2.9	0.098	0.074	0.3	0.571	0.009	0.0	0.921	0.000	1.3	0.256	0.036	0.8	0.363	0.023
Country of Birth	6.5	0.015	0.152	0.1	0.818	0.001	0.5	0.496	0.013	0.6	0.445	0.016	0.6	0.443	0.016

Supplementary References

1. J. C. Levy, D. R. Matthews, M. P. Hermans, Correct homeostasis model assessment (HOMA) evaluation uses the computer program. *Diabetes Care* **21**, 2191-2192 (1998).
2. T. C. Papadopoulos, G. K. Georgiou, C. Deng, J. P. Das, The Structure of Speed of Processing Across Cultures. *Adv Cogn Psychol* **14**, 112-125 (2018).
